# Current and near-term advances in Earth observation for ecological applications

**DOI:** 10.1186/s13717-020-00255-4

**Published:** 2021-01-04

**Authors:** Susan L. Ustin, Elizabeth M. Middleton

**Affiliations:** 1 John Muir Institute of the Environment, University of California, Davis, CA 95616 USA; 2 NASA/Goddard Space Flight Center (Emerita), Greenbelt, MD 20771 USA

**Keywords:** Terrestrial ecosystems, Earth Observing Satellites, Ecosystem processes, Physiological functions, Multispectral, Hyperspectral, Thermal, LiDAR, Radar imagers, Biochemistry, Soil properties, Species mapping, Change detection, 3D canopy structure, Topography

## Abstract

There is an unprecedented array of new satellite technologies with capabilities for advancing our understanding of ecological processes and the changing composition of the Earth’s biosphere at scales from local plots to the whole planet. We identified 48 instruments and 13 platforms with multiple instruments that are of broad interest to the environmental sciences that either collected data in the 2000s, were recently launched, or are planned for launch in this decade. We have restricted our review to instruments that primarily observe terrestrial landscapes or coastal margins and are available under free and open data policies. We focused on imagers that passively measure wavelengths in the reflected solar and emitted thermal spectrum. The suite of instruments we describe measure land surface characteristics, including land cover, but provide a more detailed monitoring of ecosystems, plant communities, and even some species then possible from historic sensors. The newer instruments have potential to greatly improve our understanding of ecosystem functional relationships among plant traits like leaf mass area (LMA), total nitrogen content, and leaf area index (LAI). They provide new information on physiological processes related to photosynthesis, transpiration and respiration, and stress detection, including capabilities to measure key plant and soil biophysical properties. These include canopy and soil temperature and emissivity, chlorophyll fluorescence, and biogeochemical contents like photosynthetic pigments (e.g., chlorophylls, carotenoids, and phycobiliproteins from cyanobacteria), water, cellulose, lignin, and nitrogen in foliar proteins. These data will enable us to quantify and characterize various soil properties such as iron content, several types of soil clays, organic matter, and other components. Most of these satellites are in low Earth orbit (LEO), but we include a few in geostationary orbit (GEO) because of their potential to measure plant physiological traits over diurnal periods, improving estimates of water and carbon budgets. We also include a few spaceborne active LiDAR and radar imagers designed for quantifying surface topography, changes in surface structure, and 3-dimensional canopy properties such as height, area, vertical profiles, and gap structure. We provide a description of each instrument and tables to summarize their characteristics. Lastly, we suggest instrument synergies that are likely to yield improved results when data are combined.

## Background

Many environmental scientists have concluded that the Earth is at or near one or more perilous climate tipping points (Krieger et al. 2009; Lenton, 2011, Lenton and Williams 2013; Brook et al. 2013; Hickman et al., 2019). Climate change interacts with and exacerbates many other environmental and societal problems. These include air and water pollution that compound health issues (Harlan and Ruddell 2011; Kan et al. 2012), especially in poor communities (Schlosberg and Colins 2014; Hallegatte and Rozenberg 2017), widespread and/or frequent droughts linked to extensive fires (Amiro et al. 2001; Littell et al. 2016), diminished resources for drinking water and irrigation (Jackson et al. 2001; Oki and Kanae 2006), and large-scale biodiversity losses (Lindenmayer and Likens 2011; Pires et al. 2018) , including species extinctions (Cahill et al. 2013). Related factors include deforestation (Green and Sussman 1990) and soil erosion (Hill et al., 2009, consequences of over-exploitation of resources (Giri et al. 2007) due to massive global conversion of natural resources for human uses (Seto et al. 2002. Documentation of all of these problems and many others are of interest to the broader ecological community at scales from local to global. This can only realistically be accomplished with satellite observations in combination with process and statistical models to reveal patterns and trends that enlighten understanding about how current conditions have developed from past environmental drivers in order to predict future conditions.

The rapid rate of global change is a defining attribute of the Anthropocene, particularly since the middle of the twentieth century, which has made traditional environmental monitoring methods obsolete for addressing the time and space scales involved. Worldwide, access to information about the ecological states of global ecosystems from field-based studies is limited, even to experts. Governments are notoriously reluctant to share data and information that exposes their own resource use and policy priorities. When data are shared, it is often incomplete, inconsistently measured, outdated, and overall inadequate for assembling a comprehensive perspective for regional to global understanding of the underlying processes at work.

### Our goal

This paper is focused on describing the primary Earth observing satellites in orbit today, with some background on their heritage, and those planned for this decade and into the 2030s. We placed emphasis on instruments and missions acquiring passive measurements (i.e., those that use the sun as their energy source) for mapping land cover, vegetation types, and ecophysiological functioning of vegetation. We include a few active systems that provide important information about biomass based on canopy metrics such as height, canopy area and volume, and other 3-D structure information that are used in ecosystem models, and models like carbon sequestration and wildfire risk. Table 1 lists the many types of ecosystem relevant satellite instruments that collect data in different regions of the electromagnetic spectrum, which we have grouped by type of measurement and pixel size. In this paper, we describe these instruments and provide examples of how they can be used to address ecological and environmental questions. We provide examples of their capabilities for monitoring ecological processes at varying temporal and spatial scales, for identifying species and species groups as well as biomes, and for quantifying local to regional biogeochemical processes relevant for natural and managed systems associated with different scales of spatial/temporal aggregation. In addition to describing new satellite technologies available today, we describe those planned for launch between 2020 and 2030 and beyond, showing how heritage programs like Landsat have led these developments. Some of these new satellite technologies are being tested by new prototype instruments currently hosted on the International Space Station (ISS), along with other demonstration flights planned for the 2020–2025 timeframe that pave the way for future operational space-based sensors and platforms. The instruments included in this review are a small subset of sensors under development by international space agencies, which we selected because of their potential interest to the ecological community and have (or proposed to have) a free and open data policy for the user communities, a policy first implemented by NASA in the Earth Observation Satellite (EOS) Program in 2000 and the Landsat program in 2011 that is now adopted by all US civilian satellites, the European Space Agency in their Copernicus suite of satellites, and a growing list of satellites from other countries.

**Table 1:** Satellites organized by management type, beginning with higher spatial resolution pixels

Spatial Resolution	Panchromatic	Multispectral	Fluorescence	Hyperspectral	Multiband TIR	LiDAR	RaDAR	GEO- Stationary
High Spatial Resolution	Landsat 7-9	VENμS		CHRIS PROBA	ASTER	GEDI	S-1	
	PRISMA	S-2		EO-1 Hyperion	Landsat 8, 9	Icesat-2		
		Landsat 4-7		PRISMA	ECOSTRESS			
		EO-1 OLI		DESIS	S-8 LSTM			
		ASTER		EnMAP				
		Landsat 8, 9		HiSUI				
				EMIT				
				SBG				
				S-10 CHIME				
Moderate Spatial Resolution	VIIRS	ENVISat	FLEX FLORIS		ENVISat		Sentinel 1	GeoCARB
		MODIS	OCO3		MODIS		Biomass	
		VIIRS	GeoCARB		VIIRS		NISAR	
		S-3 OLCI, SLSTR			S-3 OLCI, SLSTR			
					WildfireSat			
					S- 5P TROPOMI			
Coarse Spatial Resolution		AVHRR-3			AVHRR-3			S-4 UVVN, IRS
		METImage, 3MI			METImage, 3MI			S-5 UCNS
		NOAA-20 Series			NOAA-20 Series			S-6
					Sentinel-7			GOES 16 series ABI
								Himawari 8 AHI
								METEOSat MTG

### Satellites can contribute to ecological understanding

Given the size of the Earth’s land mass and the large number of nations involved in reaching consensus, it is unlikely that a consistent methodology based on ground-based measurements and/or aircraft/drone observations will achieve any short-term solution for access to accurate and repeatable and timely globally acquired and/or modeled data. We recognize and celebrate the recent efforts of several large programs recently implemented to systematically acquire consistent and repeatable ground-based observations, such as the National Ecological Observatory Network (NEON) sponsored by the US National Science Foundation and the European Cooperation in Science and Technology (COST) program. Data from these and similar programs can contribute to calibration and validation of satellite data, and they have potential to inform about local changes and their causes and begin to provide data describing regional processes. Only by employment of satellite measurements can repeatable global coverage be attained, but to date, no coalition among international space agencies (with exception of geostationary meteorological satellite programs) has been specifically charged to work together to acquire the necessary spectral and energy measurements of the Earth’s surface from space at relevant spatial and temporal scales that allow us to address the Earth as a system.

A number of existing and new satellites, and many more planned for launch over the next decade, will provide observations that enable building a critical multi-decadal time series of ecological conditions related to vegetation/biologic properties, thus providing a powerful database for scientists and decision makers, especially when combined with ground observations collected with best practices. The capabilities of these next-generation technologies have the potential to bridge the existing data gaps and revolutionize our understanding of the magnitude and speed of change across global ecosystems, but significantly more research is needed on the rates of ecosystem structure and compositional change before this vision can be realized.

To achieve the goal of monitoring the changing global environment and understanding Earth’s dynamic biogeochemical processes will require new analytical methods (e.g., machine learning algorithms) and ways to synthesize and integrate data from multiple orbital sensors to obtain accurate and timely maps of the composition and state of ecosystems, by drawing upon multiple satellites that form a “virtual constellation.” Much greater effort is needed to develop reliable models that can be independent of ground data or at least do not require it to the extent of current models. Potentially, a strategy could be developed to address the complexities of global surveys by integrating physically based models that are more precise at small scales (but lack input data at larger scales) with predictive statistical models that are accurate within the range of data viewed at coarser spatial scales from orbit. Developing synthetic models that take advantage of self-learning methods can lead the way to further utilizing the “free and open” global satellite databases that are already accessed by many expert users, thus leading to greater acceptance of satellite findings and integration into predictive global models.

Since the first polar orbiting satellites for land observations were launched in the early 1970s, sun-synchronous polar orbiting satellites have been key to observing surface properties because they always pass a given latitude at the same time of day, thus making their data more interpretable by reducing the impact of diurnally variable illumination conditions although seasonal differences still remain. Because the orbits are actually 8–10° off north (90°), the satellite makes an oblique descending or ascending overpass track and, thus, crosses ~ 1–3 local time zones on both the descending and ascending paths, consequently, the time reference for LEO orbits is the equatorial overpass time. Most LEO satellites measuring reflected solar energy have a mid-morning equatorial crossing time because studies (e.g., Harrison et al. 1980, 1983, 1990; Whitcraft et al. 2015) have shown that while the timing of maximum cloud cover varies globally, seasonally, and latitudinally, the midmorning to midday periods are less cloudy in the mid-latitudes that contain the largest land area.

Multidate information, especially when consistently acquired with sun-synchronous satellite data, captures phenological and seasonal patterns that greatly improve mapping vegetation functionality (Wilson and Sader 2002). This is necessary for accurate prediction of crop yields in agriculture (Lobell et al. 2015) and for detecting disturbances such as forest logging (Franklin et al. 2000), among many other applications. The probability of capturing specific events or trends is improved when data from several satellites can be combined to build a time series, such as with actual or virtual constellations.

There are many attributes of space-based instrument performance and platform characteristics that together determine the value of their environmental observations including orbit type, swath width, pixel size, overpass repeat interval, and spectral information (wavelength range, number of bands, band width, sensitivity/noise, etc.). All of these are relevant to the interpretation and utility of information that can be retrieved. Additional performance characteristics include the viewing mode of the instrument (whiskbroom or push broom), orbit type (e.g., polar or geostationary), orbit inclination angle (degrees off true north/south), orbit altitude, and factors such as mass and size, fuel load, power, downlink capabilities, and onboard processors.

There are important differences in the energy sources underlying passive and active measurements from space. Passive optical observations include reflected shortwave solar radiation (i.e., radiance, and the more useful derived measure, reflectance), solar-induced emitted radiation (e.g., fluorescence), and longwave radiation emanating from the surface or atmosphere due to their thermal properties. Active sensors (radar and LiDAR) emit radiation at specific wavelengths that are transmitted to the Earth’s surface and measure the energy scattered back to a detector. Processing of satellite data requires a basic understanding of the physical, chemical, and structural properties underlying the measurements. We provide examples of these characteristics in discussions of the satellite instruments and measurements and how they relate to information derived from the data. We primarily focus on vegetation in this review because plants serve as the dominant regulators of energy, chemical, and mass transfer within the Earth's land system.

**Fig. 1:**
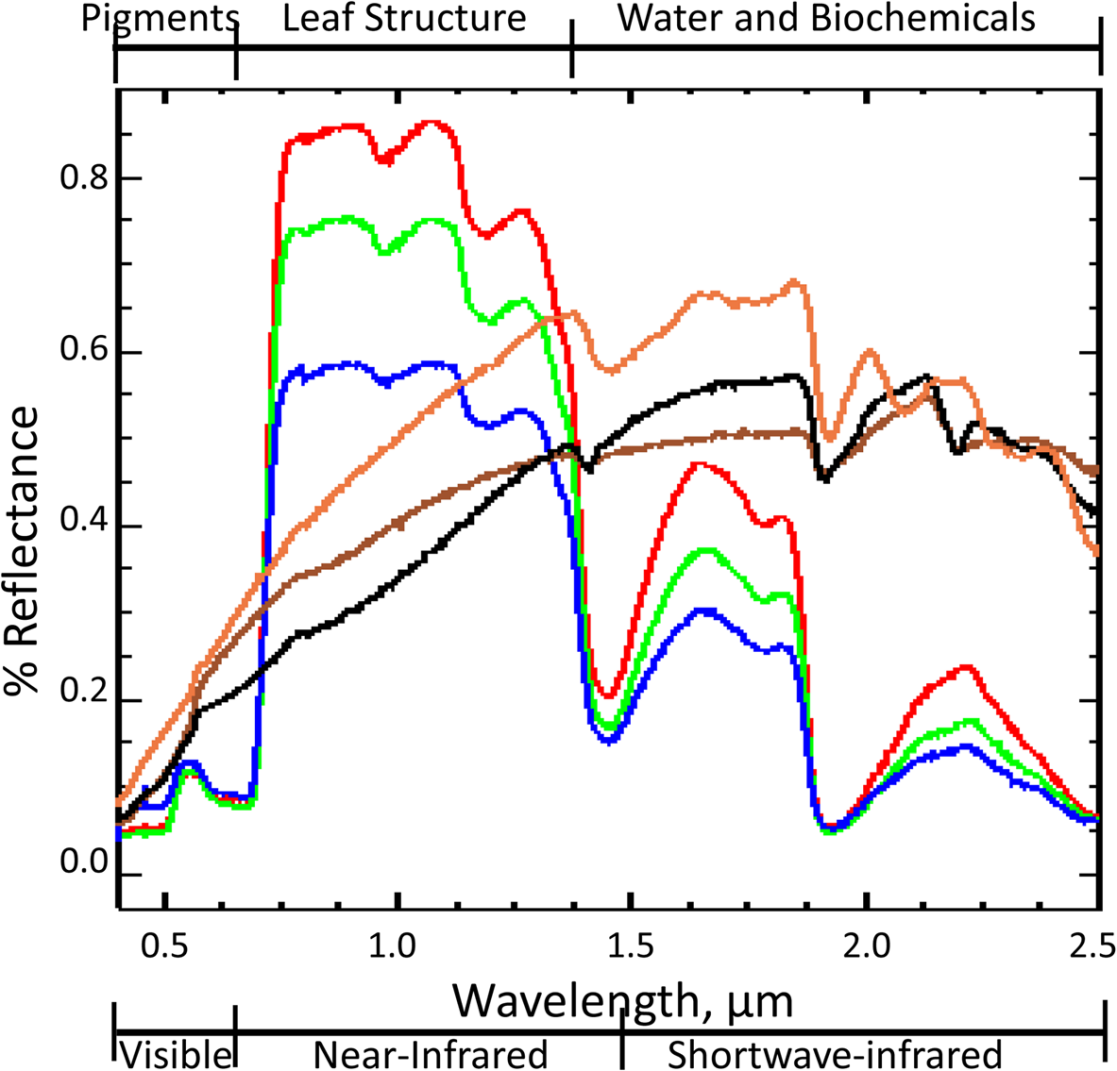
Spectra of typical green plant and soil materials from the central California Coast Range: red willow (*Salix laevigata* leaf, red); valley oak (*Quercus lobata* leaf, green); blue oak (*Q. douglasii* leaf, blue); dry annual grass leaf, orange; Franciscan formation complex non-serpentine greenstone soil, brown; and Butano sandstone, black. Common leaf biochemicals are shown along the upper edge of the figure indicating regions where absorptions occur. The commonly used names for these regions of the solar spectrum are shown below the wavelength axis. Spectra are from the Environment for Visualization of Images (ENVI) spectral library ver 4.8 and were donated to ENVI and published by Dr. Christopher D. Elvidge 1990. Soil names follow Coleman 2010

The shape of the reflectance spectrum of healthy leaves in the visible (VIS; 0.4–0.7 microns, μm) to near-infrared (NIR, 0.7–1.5 μm) wavelengths (Fig. 1, red, green, and black lines) is determined by photosynthetic pigments, especially chlorophylls absorbing solar radiation primarily in the blue and red wavelengths and by carotenoids that broaden the spectrum across the ultraviolet and into the green wavelengths. Consequently, because most energy is absorbed in the VIS wavelengths, reflectance there is low, around 10% or less, but it sharply increases in the transition to the NIR where reflectance is high because absorption is low. In healthy vegetation, this transition region from red to NIR wavelengths (~ 0.70 μm wide) is termed the “red edge” and is physically the long wavelength edge of chlorophyll absorption. Thus, the presence of low reflectance in red wavelengths and high reflectance in NIR wavelengths is a unique pattern that identifies live green foliage.

The shape of a spectrum is determined by the chemicals that absorb at specific wavelengths and the scattering processes that scatter photons either reflecting them from the surface or transmitting them through a semitransparent object, such as a leaf. It is the subtle differences in the shape of representative spectra as shown in Fig. 1 that provide the information to discriminate different plant types, and even species, as well as phenological changes that occur as foliage ages. For example, the dry leaf spectrum (orange line) no longer retains evidence of photosynthetic pigments and is more similar in shape to the soil spectrum (brown line). These changes are part of a continuum; when leaves dry out, their reflectance increases across the infrared (IR) spectrum, often becoming 50% or more of the received solar irradiance. In the dry leaf, you see many wiggles in the longer wavelengths of the spectrum (between 1500 and 2500 nm), due to absorptions by cellular biochemicals like cellulose, lignin, sugars, starches, and other carbon-rich compounds to proteins, oils, and other substances. In some cases, other chemical elements and compounds can be specifically identified (e.g., Asner and Martin 2016) but typically these are lumped together and termed “dry plant matter” (Féret et al. 2008) or “dry biomass” (e.g., Qi et al. 2014) As dead leaves weather into soil and become humus, their spectra gradually change from plant-like to soil-like.

In green leaves, the two narrow absorption features seen between 0.95 and 1.05 μm and between 1.15 and 1.25 μm are due to liquid water in plant tissues, which is present in large amounts as generally water mass ≈ leaf dry weight. Other strong water absorption bands are centered at 1.44 μm and 1.92 μm, the wavelengths where atmospheric water vapor strongly absorbs energy. Since atmospheric water vapor generally saturates measurements at these wavelengths, spectral bands in these regions are not generally included in Earth observing satellites. Green leaves exhibit a pattern of decreasing reflectance across the spectrum between 1.0 and 2.5 μm, due to absorption by the high water content of green leaves. These water absorptions are due to secondary absorptions caused by overtones and vibrational combinations. There are few other absorption features observed in fresh leaves, except for a small one around 1.75 μm related to cellulose and lignin properties of cell walls. The dry leaf has strong absorptions at 2.06 μm, a shallow but broad feature from 1.68 to 1.88 μm, and a doublet (overlap of two absorption features) from 2.2 to 2.37 μm. These features are thought to relate to several biochemical constituents, the most abundant of which are cellulose, lignin, and nitrogen in proteins (Kokaly et al. 2009).

The shortwave infrared (SWIR) region is most valuable for geologic studies of minerals due to the presence of many absorption features across the spectrum. Soils have their own biochemical constituents such as different clays, mineral oxides, and organic matter that contribute to the shapes of their spectra. However, interpretations remain challenging because of the complexity of multiple absorbing compounds and their interactions with light at the leaf or soil sample level but become more complex in imagery due to averaging of multiple materials within a pixel. In Fig. 1, the soil spectrum has a slight convex curvature between 0.75 and 1.4 μm indicative of little or no soil organic matter. In highly organic soils (e.g., peat), this shape would be strongly concave. Soils can also have strong absorption features in the iron oxide NIR bands around 0.9 μm and higher reflectance in red wavelengths and the 2.0–2.5-μm region associated with clay minerals, organic carbon-rich materials, and other minerals, such as carbonates and gypsum. Both the soil and rock spectra in Fig. 1 have an absorption feature at 2.2 μm that is absent in dry leaves. Obviously, the retrieval of biochemical information and processes from spectral data, especially from space, is a complex but compelling task, which is still an active area of research.

Unlike laboratory spectra as shown in Fig. 1, most satellite instruments are multispectral imagers, with between four to twenty or so spectral bands. We describe the most important ones useful for landscape and ecology research that typically have their several bands placed to observe reflectance in the major spectral features shown in Fig. 1, such as bands in the green, red, near-infrared for VIS-NIR (VNIR) sensors, and for some there are additional bands in the SWIR or thermal regions.

In this review, we present a summary of the few hyperspectral imagers (also called imaging spectrometers) in orbit or planned for orbit. Hyperspectral (or spectroscopy-based) imagery allows identification of detailed chemical composition because the large number of bands, especially when they are narrow and contiguous or overlapping, can directly describe relevant absorption or reflectance features. This type of data provides more information for mapping landscapes and image classification than traditional multispectral data, and by linking changes in reflectance in different bands or wavelengths to the health of plants and soils that are linked to various environmental factors that might impact them. These include weather, for example, droughts and floods, insects, pollution, changes in species composition from invasive species, and changes in agricultural management. Other factors include variation or changes in ecosystem properties (Asner 1998; Ustin et al. 2004; Schimel et al. 2015), canopy structure, and biochemistry (Asner et al. 2008, 2015; Kokaly et al. 2009; Ustin et al. 2009). Physiological processes include photosynthesis, evapotranspiration, absorption of photosynthetically active radiation, photosynthetic light use efficiency, phenology (Ulsig et al. 2017), and stress responses (Glen et al. 2007; Middleton et al. 2009, 2016, 2017, 2018; Hilker et al. 2010; Ustin and Gamon 2010; Serbin et al. 2011; Clevers and Kooistra 2012; Mohammed et al. 2019; Zhang et al. 2014a, 2014b).

Hyperspectral data from space can provide spectrally dense information that is sufficient to retrieve many key biochemical signals such as plant constituents (Kokaly et al. 2009; Ustin et al. 2009) and canopy structure information (Huesca et al. 2019). For vegetation, these include chlorophyll (Gitelson et al. 2003; Féret et al. 2017), carotenoid and anthocyanin contents (Gitelson et al. 2007, 2009; Gitelson and Solovchenko 2018), water content (Ustin et al. 1998; Datt 1999; Ceccato et al. 2001; Colombo et al. 2008; Yerba et al. 2013), nitrogen content (Kokaly 2001; Asner and Martin 2008), cellulo-lignin (Serrano et al. 2002; Kokaly et al. 2009; Asner et al. 2015), and dry leaf mass (Jacquemoud et al. 1996; Qi et al. 2014) and other parameters like leaf area and leaf mass area (Jacquemoud et al. 1996; Serbin et al. 2014; Féret et al. 2017). Similarly, discernible soil chemical properties include clay minerals, organic matter, iron oxides, and geologic minerals (Ben-Dor et al. 2009; Ge et al. 2011; Palacios-Orueta and Ustin 1998; Palacios-Orueta et al. 1999; Stevens et al. 2010). The U.S. Geologic Survey Spectral Library (version 7) is available online as a reference to relate chemical composition and spectral properties of many common Earth materials, at https://www.usgs.gov/labs/spec-lab (Glen et al. 2007; Kokaly et al. 2017). Other online databases are the ECOSTRESS spectral library that incorporates the earlier ASTER library (https://speclib.jpl.nasa.gov/), and the EcoSIS leaf spectral library (https://ecosis.org/).

## The Landsat Heritage

**Fig. 2:**
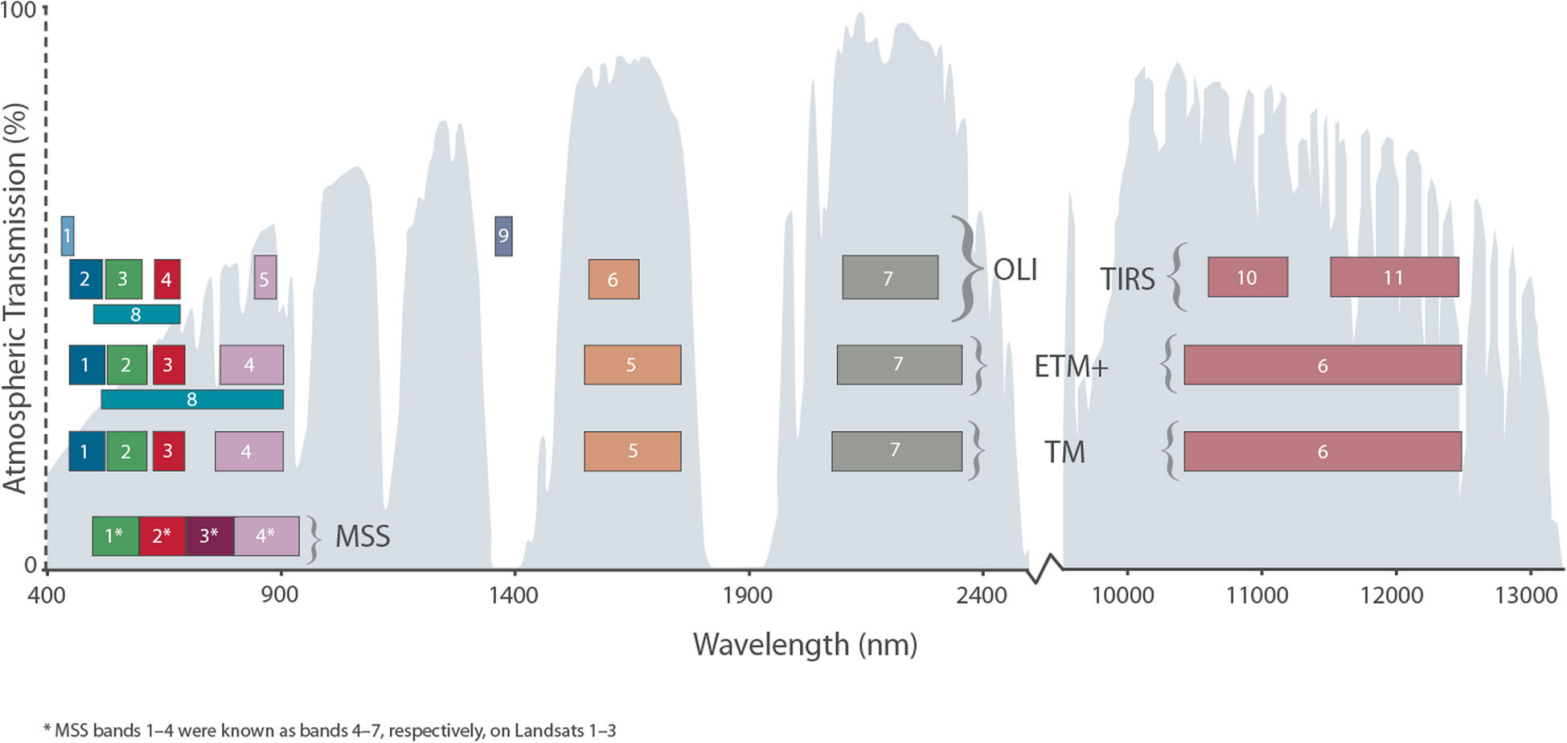
Landsat bandpass locations, band width, and band number for optical and thermal imaging sensors for the Landsat satellites: The MultiSpectral Scanner (MSS) on Landsats-1–3 *(Bottom row).* L-3 had 80 m pixels and a thermal band (not shown) at 240 m pixel resolution; L-3 was terminated March 1979. The Thematic Mapper (TM) *(Row 2)* on Landsat-4 and Landsat-5, introduced 30 m pixels for optical bands, adding three new bands (blue [1] and two SWIR [5, 7]) and a 120-m thermal band [6]. The Enhanced Thematic Mapper (ETM+) *(Row 3)* on Landsat-7 is similar to L-4 and L-5 but with a 15 m panchromatic Visible-Near-Infrared (VNIR [8]) band and a TIR band with 60 m pixels [6]. The Operational Land Imager (OLI) *(Row 4)* on Landsat-8 and Landsat-9 narrowed some bands and added two new bands [1, 9]; the 15-m pan band was limited to VIS [8]; and the TIR became two thermal bands TIRS1 and TIRS2 [10, 11] with 100 m pixels. The gray background shows regions of light (energy) transmission through the atmosphere to the Earth’s surface; in white areas, no solar energy gets to the land surface and back to the satellite, or for the thermal range, emitted from the surface. The common names for different wavelength regions are shown across the top of the figure. The numbering of the bands represents the order that the band was selected for inclusion on each Landsat. The atmospheric transmission values for this graphic were calculated in MODTRAN (MODerate resolution atmospheric TRANsmission computer code, MODTRAN 6, 2014) for a summertime mid-latitude hazy atmosphere (circa 5 km visibility). Graphic created by L. Rocchio & J. Barsi. Figure from https://landsat.gsfc.nasa.gov/about/technical-information/

The first imaging satellite to demonstrate the potential for Earth observations based on the Earth’s reflectance properties was the Earth Resources Technology Satellite or ERTS, later referred to as Landsat-1 (1972–1978). The Multispectral Scanner (MSS) on L-1, L-2, and L-3 revealed the general benefits of spatial, spectral, and temporal properties of landscapes (Table 2, Fig. 2). These and the Landsat satellites that followed, established the morning descending overpass around 10:00 as a standard for land observation. Starting with the Thematic Mapper instrument on L-4, the Landsats have added new narrower spectral bands so that it has become easier to tell general categories of land cover apart based on their spectral information, as illustrated in Fig. 1.

**Table 2:**
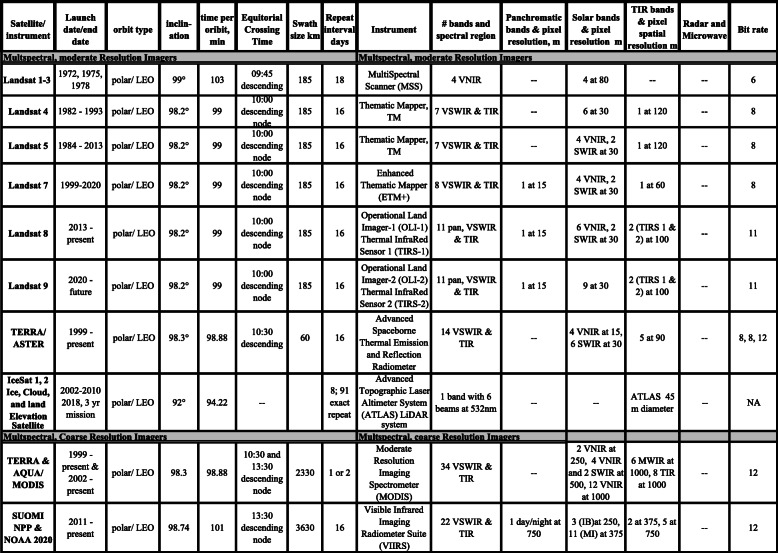
Multispectral satellites of moderate spatial resolution

The differences between whiskbroom and push broom sensors may seem arcane for many ecologists, but the acquisition mode impacts the data quality and should be noted. All of the Landsat satellites prior to L-8 (L-1 through L-7) were whiskbroom (cross-track) scanning instruments, meaning that they used mirrors that sweep back and forth perpendicular to the flight track and reflect light onto a single or a few detector(s) that scan pixel by pixel from the leading edge of the 185-km wide swath to the opposite edge and then return to start a new collection row. The moving mirrors of whiskbroom scanners tend to be large and complex to build, create spatial distortions in the data that must be corrected, and provide little time to collect light for the detectors. Thus, this limited how small pixels could be. An advantage, however, is that whiskbroom scanners have only a few detectors to keep calibrated. Push broom (along track) scanners are utilized by the most recent Landsats (L-8 and L-9), and almost all new satellite instruments, using a line (or an array, i.e., several lines) of detectors arranged perpendicular to the flight direction of the spacecraft. As the spacecraft flies forward, the image is collected one line (or array) at a time, with all pixels in a line measured simultaneously. A push broom scanner receives a stronger signal than a whiskbroom scanner because its longer dwell time enables higher sensitivity, which thus allows narrower bandwidths, better radiometric and spatial resolution, and higher geometric accuracies. Push broom scanners are lighter, smaller, and less complex because of fewer moving parts than whiskbroom scanners. However, their major disadvantage is the calibration effort required for the large number of detectors. If the detectors are not perfectly cross-calibrated, stripes can result in the data. Today’s more advanced, stable, and well-calibrated instruments minimize these problems.

### Landsats 4 through 9

The longest continuous record of satellite data collection is from the joint Landsat satellite program of the National Aeronautics and Space Administration (NASA) and the U.S. Geological Survey (USGS). While the Landsat Program technically goes back to 1972, it is only the period since the development of the Thematic Mappers on Landsat-4 (L-4) in 1982 and Landsat-5 (L-5) in 1984 that environmental applications using satellites became a reality. Landsat-5 flew a remarkable 29 years before it was decommissioned in 2013. L-4 and L-5 routinely observed the Earth with 30 m pixels every 16 days, and greatly expanded the types of land cover and environmental studies that could be done, due to the technological advances in the Thematic Mapper (TM) (Fig. 2). The TM added bands in the solar spectrum in the VIS to SWIR regions and included one thermal band in the 10-μm region of peak Earth emissions. These data provided opportunities to study land surface conditions and temperature patterns over space and time (Table 2, Fig. 2). Pixels were now 30 m (i.e., 30 m × 30 m or 900 m^2^ ) in the solar bands instead of 80 m (6400 m^2^) and the data could resolve 128 gray layers (2^8^ bits) compared to MSS’ 64 (2^6^ bits), which greatly improved the range of objects that could be distinguished.

One year after the 1984 launch of L-5, when interest in using satellite data for environmental and ecological studies was rising, a US policy to incentivize commercialization of space resulted in transferring the Landsat program to the Earth Observation Satellite Company (EOSAT) (Unninayar and Olsen 2008). Under commercialization, Landsat data cost ~ $4400 per standard scene, an area ~ 185 km × 185 km that reduced the number of users to a small group. By 1992, Landsat data demand had declined to near zero, because of high data costs. This prompted Congress to return the Landsat program to NASA and USGS, and instructed the Landsat Program Management to build L-7 (L-6, under EOSAT management failed to achieve orbit in 1990). Landsat-7 was launched in April 1999 and carried a new instrument, the Enhanced Thematic Mapper (ETM+) that included a 15 m panchromatic (“pan”) band, a broad VIS band with images that mimicked a black and white photograph, and a TIR band with 60 m pixels, which for L-5 was 120 m pixels (Table 2, Fig. 2). For the first time, L-7 included an absolute radiometric calibration standard of 5% along with high geometric accuracy. In 2001, after nearly 2 years of successful new ETM+ image collections worldwide, the private-sector Space Imaging Company (formerly EOSAT) returned operations and rights to Landsat data to the US Government, allowing USGS to sell Landsat data at a much-reduced cost.

Landsat-7’s ETM+ and Landsat-8’s Operational Land Imager (OLI) which launched in 2013 maintained the original 6 multispectral bands from the earlier TMs (although adjusting band centers and band widths, and adding additional bands). For L-7, the spectral bands included four VNIR bands and two bands in the SWIR region (Table 2, Fig. 2) and one 10 μm thermal infrared (TIR) band with 60 m pixels. Technical advances allowed more Landsat-7 data collections over land areas around the world. However, the L-7’s ETM+ suffered a Scan Line Corrector (SLC) failure in May 2003, which caused it to image the Earth in a “zig-zag” fashion along each orbit, resulting in some areas being imaged twice while others were not imaged at all. This failure affected ~ 22% of each imaged scene, for which a Gap-Fill product was developed that used recent archived data to fill in missing data. L-7 continued to acquire data in its original orbit until early 2017 and is long past its normative life and the satellite is now drifting in orbit. To extend the life of future satellites, NASA announced plans in 2016 for the Restore-L Mission (now called OSAM-1, an acronym for On-Orbit Servicing, Assembly, and Manufacturing 1), now slated for launch in 2022. It is designed for refueling and servicing with in-space robotic precision assembly.

NASA’s Earth Observing-1 (EO-1) technology demonstration satellite was launched in late 2000 into a tandem orbit, trailing L-7 by 1 min, and carrying a prototype for L-8, which was launched 13 years later. EO-1’s Advanced Land Imager (ALI) demonstrated numerous improved technologies that were incorporated into the design of L-8, including two new bands (one for coastal aerosols and one for detecting cirrus cloud ice crystals (Gao et al. 1993), with its long-track detector arrays (more than 7000 detectors per band!) reporting 12-bit radiometric resolution. The EO-1 also carried the first civilian hyperspectral VSWIR instrument in space, Hyperion, the precursor to NASA’s upcoming Surface Biology and Geology (SBG) mission currently under development and contributed to two European missions, PRISMA and EnMAP, all of which are discussed in later sections. The EO-1 mission successfully collected data worldwide over its 17 years of operation (2000–2017). Both instruments on EO-1 were trail blazers with numerous new applications and technology enhancements arising from this mission as reported by Middleton et al. (2013) and in other articles in a 2013 Special Issue of *Journal of Selected Topics in Applied Earth Observations and Remote Sensing (JSTARS*; Ed., E.M. Middleton).

The USGS was finally able to adopt a free and open Landsat data policy in 2008 (Woodcock et al. 2008), when it became the National Land Imaging (NLI) program at the USGS Earth Resources Observation and Science (EROS) Center (Loveland and Dwyer 2012). The availability of “free and open” Landsat data produced an exponential increase in its use that continues unabated today and has benefitted society by a growing list of applications and stakeholders (Cohen and Goward 2004; Wulder et al. 2012; Roy et al. 2014). This key policy change enabled development of a diverse array of ecological applications and widespread use for biodiversity conservation (Turner et al. 2015). Furthermore, the success of this US data policy was adopted by the European Space Agency (ESA) for their Copernicus satellites and is now being adopted by other countries. All of the satellite instruments reviewed in this paper follow this policy and their data are available, although for a few, some restrictions limit research use. Landsat data are available at usgs.gov/Land-Resources and images can be viewed on these web sites: earthexplorer.usgs.gov, GloVis.usgs.gov and Landsatlook/usgs.gov.

The impact of the Landsat satellites, the workhorses for all satellite studies of terrestrial and shallow water environments, is demonstrated by the 27,392 papers identified in Web of Science (07/12/20). The accomplishments of the Landsat Program were poignantly captured in the recent book, *Landsat’s Enduring Legacy* (Gower et al. 2017) and the Case Studies 2018 eBook, *Landsat Benefiting Society for Fifty Years* (Rocchio et al. 2018). Thousands of environmentally focused papers have been published using Landsat data, including the first National Land Cover Dataset in the 1990s for the conterminous USA (Vogelmann et al. 2001) and continental scale maps of land cover change (Townsend and Walsh 2001; Hansen et al. 2014) and the forestry map of Canada (Wulder et al. 2003). Notable ecological studies have addressed forest health (e.g., monitoring woolly adelgid outbreaks in eastern hemlock; Royle and Lathrop 1997), forest survival after wildfire (Kushla and Ripple 1998; Miller and Yool 2002; Karlson et al. 2015), mapping the distribution of semiarid vegetation and environmental controls on species abundance patterns (Smith et al. 1990a, 1990b), and innumerable other ecosystem applications from agriculture (Leslie et al. 2017; Gumma et al. 2020) to wetlands (Johnson and Barson 1993; Tang et al. 2004; Schneider et al. 2009; Halabisky et al., 2016, biodiversity hotspots (Gould 2000; Helmer et al. 2002; Brandt et al. 2013; Cavender-Bares et al. 2016), alpine ecosystems (Dozier 1989; Bolton et al. 2018; Gianinetto et al. 2019), the arctic (Stow et al. 2004; Huang et al. 2017; Griffin et al. 2018), and to dry lands (Qi et al. 2000; Langley et al. 2001; Bradley and Mustard 2005; Chen et al. 2005; Sohn and Qi 2005) and landscape structure (Saunders et al. 2002) among other applications.

### Operational land imaging: Landsats of today and tomorrow

**Fig. 3:**
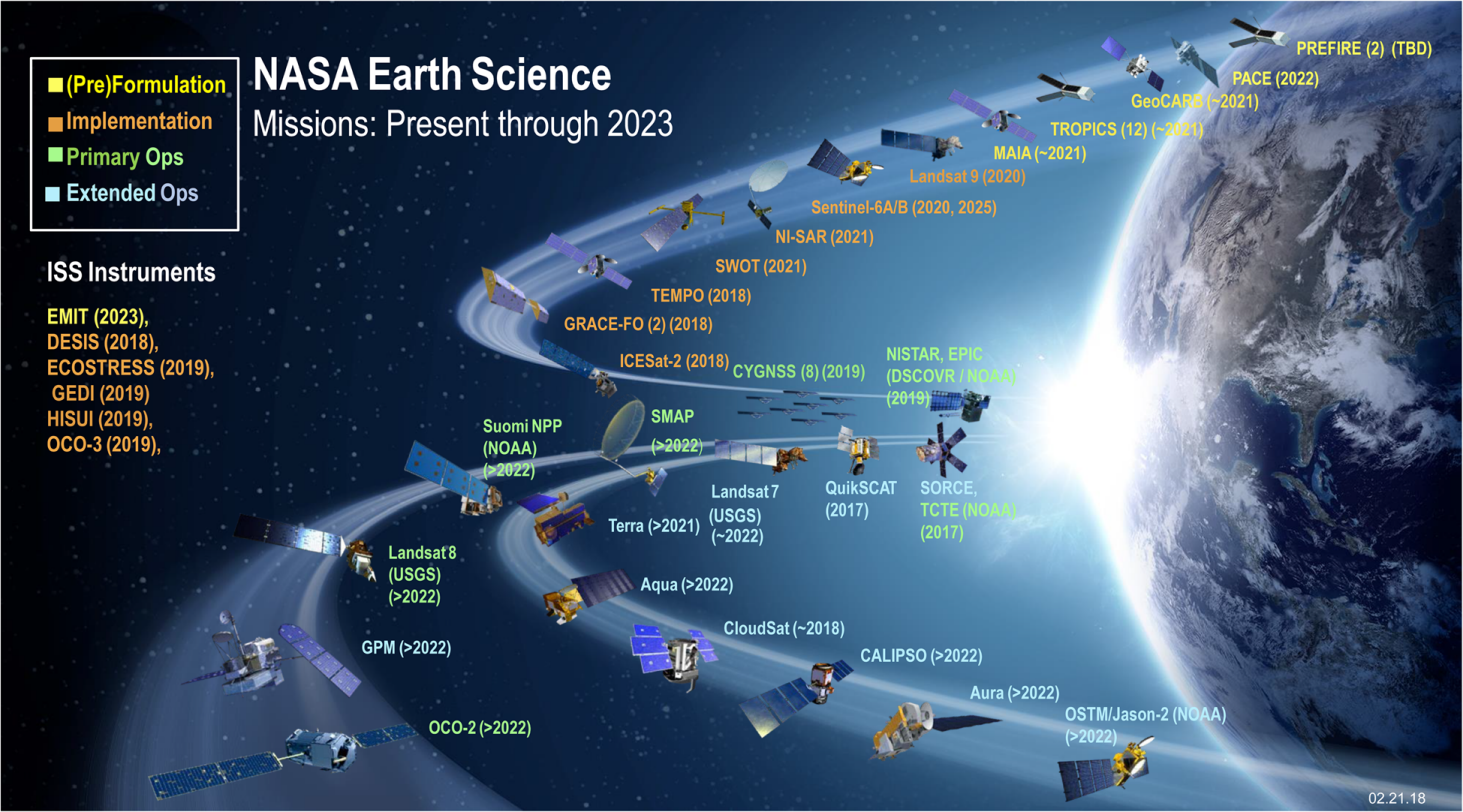
NASA’s active Earth science satellite missions in 2019, including those on the International Space Station (modified from www.eospso.nasa.gov). NASA's full Earth Observation fleet includes instruments measuring ocean, land, and atmospheric chemistry, processes and structures

In recent years, Landsat-8, launched in 2013, has routinely collected imagery over all continents and large islands (> 10 km^2^) worldwide and is universally hailed as the gold standard for Earth multispectral (MS) observations. L-8 ushered in a 30 -year vision for operational U.S. Landsat satellites under the Sustainable Land Imaging program. L-8 and L-9 (Table 2, Fig. 3) carry two new instruments, the 9-band Operational Land Imager (OLI) and the 2-band Thermal InfraRed Sensor (TIRS; designated as bands 10 and 11) (https//landsat.gsfc.nasa.gov/landsat-8/). Band 9 from OLI-1 (L-8) and OLI-2 (L-9) identifies atmospheric ice crystals enabling detection of cirrus clouds, an important advance as cirrus clouds are hard to identify and contaminate a large percentage of Landsat images (and images from other sensors), reducing the dynamic range of observations, thus affecting their quality and usability by reducing the potential to distinguish objects with similar reflectance. L-9 will fly in the same orbit as L-8 but positioned 180° offset to provide 8-day repeat data (at the equator). However, because L-8 has already exceeded its design life of 5 years, it may fail, but it does carry fuel for 10 years of operations, through 2023.

One of the most exciting new developments is that the European Space Agency’s Sentinel-2 satellites (S-2A and S-2B) fly in the same orbital path and similar time of day as L-8 and L-9 (Landsats at 10:00 and Sentinel-2s at 10:30 equatorial crossing times), but each S-2 has a 10-day repeat cycle. Although the Sentinel-2A and Sentinel-2B multispectral imagers (MSI) have higher spatial resolution for their 8 VNIR bands (either 10 or 20 m) than the Landsat OLIs, the two types of instruments share several similar spectral bands and data sensitivity, so their data can be combined into a time series to increase the frequency of data collection, potentially to 1–3 days after L-9 is launched (see “Sentinel-2” discussion below). Additionally, Sentinel-2s have three new “red edge” bands and a new NIR band (~ 940 nm) to aid atmospheric correction for water vapor. Combining data from Landsat-8 (and soon L-9) with Sentinel-2A and -2B through harmonization has proved to be fruitful for retrieval of chlorophyll in vegetation and other surface features (Fig. 3).

#### The 10th Landsat, Landsat-Next

The Sustainable Land Imaging (SLI) science requirements for the tenth Landsat, currently designated Landsat-Next, are under development and review (Masek, 2018), working toward a launch date before 2030. The draft plans are summarized for public distribution and are found online: https://beta.sam.gov/opp/ba6bec027510abc30e1f6fdafa74228c/view?keywords=landsat&sort=-relevance&index=&is_active=true&page=1). The new instrument package will discontinue the panchromatic band but will include at least six new bands matching Sentinel-2 bands, raising the total to 16 bands from the eleven on L-8 and L-9. All VSWIR bands would maintain the traditional 30 m GSD, except for three bands related to atmospheric corrections which will be 60 m GSD (B1, coastal aerosol; B9, water vapor; B10, cirrus), and three TIR bands will achieve GSD at 60 m (vs. 100 m of L-8/9). Tables 2, 3, 4, 5, and 6 provide information on current and planned satellites through the mid-2030s. The launch dates in the tables are announced by the agencies but should be considered “goals” as the schedules are routinely delayed, for every problem from bad weather to disrupted supply chains, and for the many reasons that the thousands of steps from concept to launch and many dependencies involved, result in delays.

**Table 3:**
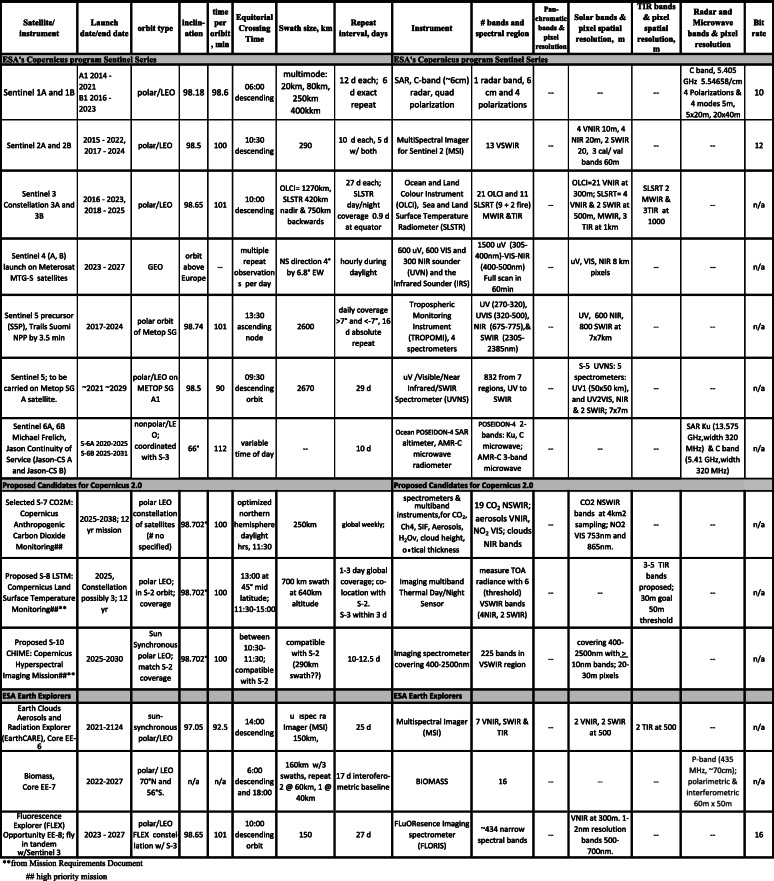
European Space Agency’s Copernicus program of record

**Table 4:**
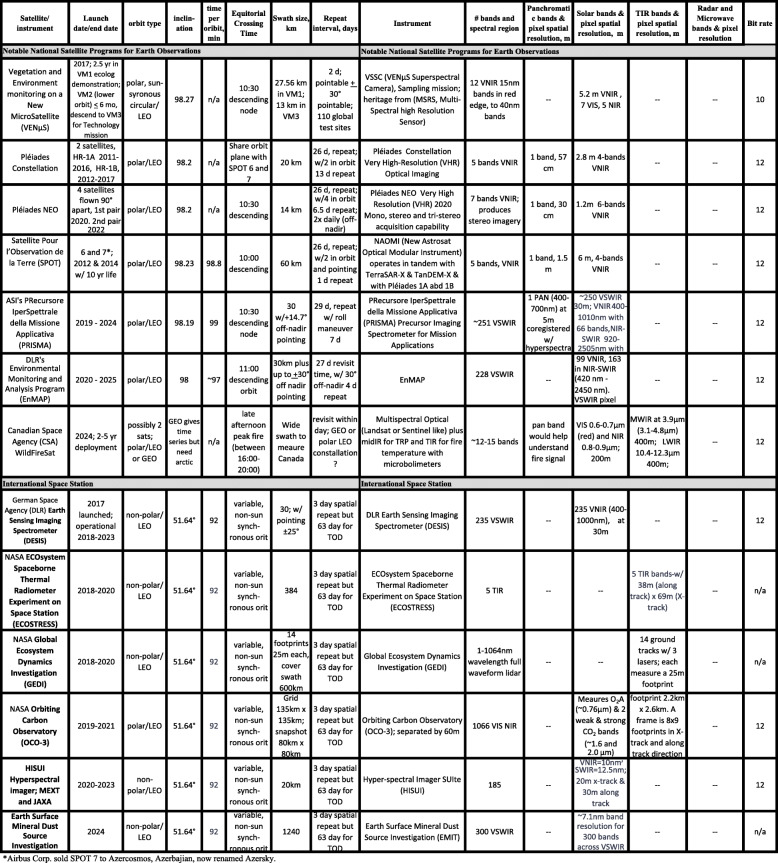
Notable other satellite programs for Earth observation, including the International Space Station

**Table 5:**
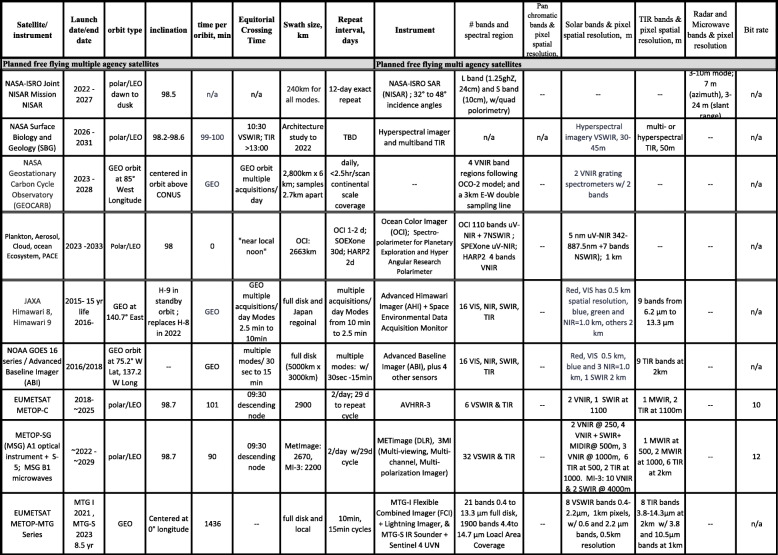
Planned free-flying satellites from multiple agencies

**Table 6:** Satellites organized by period of expected flight, and by orbit, sun-synchronous LEO, or GEO, or low-inclination angle. Older instruments are higher in the order and newer instruments lower on the list

	Sun-Synchronous	Sun-Synchronous	Low Inclination	Sun-Synchronous	Low Inclination	Sun-Synchronous	Low Inclination	Sun-Synchronous	Low Inclination	Sun-Synchronous	Low Inclination
	Orbit	Orbit	Orbit	Orbit	Orbit	Orbit	Orbit	Orbit	Orbit	Orbit	Orbit
***Study Site Size***	***Plot,*** ≤ ***500 m***^***2***^	***Local, 1 km***^***2***^		***Landscape, 10 km***^***2***^		***Regional, 100 km***^***2***^		***National, 1000 km***^***2***^		***Continental-Global,*** ≥ ***0.25 deg***	
***Target***	***Land & Inland Water***	***Land & Inland Water***		***Land & Inland Water***		***Land & Inland Water***		***Large Features/Areas***		***Area Averages***	
Examples	Urban/Transects	Agriculture/Forests/ Deserts		Agriculture/Forests/ Deserts		Watersheds/Islands		Ecosystems/Biomes/		Terrestrial Tiles	
	Flux Towers	Lakes/Wetlands/Coasts		Lakes/Wetlands/Coasts		Ecosystems/Bays/Lakes/Oceans		Coastlines/Plumes/Oceans		Ocean Patterns	
**Archive**	EO-1/Hyperion^$^	EO-1/Hyperion^$^	DESIS^$^	EO-1/Hyperion^$^	DESIS^$^	MODIS					
	EO-1/ALI^$^	EO-1/ALI^$^	ECOSTRESS^$^	EO-1/ALI^$^	ECOSTRESS^$^	VIIRS					
	CHRIS-PROBA^$^	CHRIS-PROBA^$^	GEDI^$^		ISS/GEDI^$^	MERIS	ISS/GEDI^$^				
	Landsats 1-5^$^	Landsats 1-5^$^	OCO-3^$^	Landsats 1-5^$^	OCO-3^$^	Landsats 1-5^$^	ISS/OCO-3^$^				
	Landsats 7-8	Landsats 7-8	HISUI^$^	Landsats-7-8	HISUI^$^	Landsats-7-8		Landsats 7-8			
	ASTER^#$^	ASTER^#$^					Himawari-8		Himawari-8		Himawari-8
	Sentinel-1	Sentinel-1A, 1B		Sentinel-1 A, B		Sentinel-1 A, B	GOES-16,-17	Sentinel-1 A, B	GOES-16,17	Sentinel-1 A, B	GOES-16,17
	Sentinel-2A,B	Sentinel-2A,B		Sentinel-2A,B		Sentinel-2A,B		Sentinel-2A,B		Sentinel-2 A. B	
				Sentinel-3A,B		Sentinel-3A,B		Sentinel-3A,B		Sentinel-3A,B	
	VENμS^$^	VENμS^$^		VENμS^$^							
		PRISMA^$^		PRISMA^$^		PRISMA^$^					
						S-5P TROPOMI		S-5P TROPOMI		S-5P TROPOMI	
				Terra, Aqua		Terra, Aqua		Terra, Aqua		Terra, Aqua	
				Suomi NPP VIIRS		Suomi NPP VIIRS		Suomi NPP		Suomi NPP	
				MERIS (EnviSat)		MERIS (EnviSat)		MERIS (EnviSat)		MERIS (EnviSat)	
								NOAA 20		NOAA 20	
**2021-2025**	Landsats 8^#^-9	Landsats 8^#^-9		Landsats 8^#^-9	Sentinel-4	Landsats 8^#^-9	Sentinel-4	Landsats 8^#^-9	Sentinel-4	Landsats 8^#^-9	Sentinel-4
	ASTER^#$^	ASTER^#$^									
	Sentinel 1A, 1B	Sentinel 1A, 1B		Sentinel 1A, 1B		Sentinel 1A, 1B		Sentinel 1A, 1B		Sentinel 1A, 1B	
	Sentinel-2A,2B	Sentinel-2A,2B		Sentinel-2A,2B	Sentinel-6	Sentinel-2A,2B	Sentinel-6	Sentinel-2A,2B	Sentinel-6	Sentinel-2A,2B	Sentinel-6
				Sentinel-3A,B,C		Sentinel-3A,B,C		Sentinel-3A,B,C		Sentinel-3A,B,C	
			DESIS^$^		DESIS$	S-5P TROPOMI		S-5P TROPOMI		S-5P TROPOMI	
			ECOSTRESS^$^	Sentinel-5	ECOSTRESS^$^	Sentinel-5		Sentinel-5		Sentinel-5	
	PRISMA^$#^	PRISMA^$#^	GEDI^$^	PRISMA^$#^	ISS/GEDI^$^						
	EnMAP^$#^	EnMAP^$#^	OCO-3^$^	EnMAP^$#^	OCO-3^$^						
	EE-7,BIOMASS^$^	EE-7, BIOMASS^$^	HISUI^$^	EE-7, BIOMASS^$^	HISUI^$^	EE-7, BIOMASS^$^		EE-7, Biomass$		EE-7, Biomass$	
	EE-8, FLEX	EE-8, FLEX	EMIT^$^	EE-8, FLEX	EMIT^$^	EE-8, FLEX		EE-8, FLEX		EE-8, FLEX	
									Himawari-8, -9		Himawari-8, -9
						WildFireSatS	GOES-16, 17	WildFireSatS	GOES-16, 17		GOES-16, 17
				NISAR		NISAR	MTG-I A,B^&^	NISAR	MTG-I A,B^&^	NISAR	MTG-I A,B^&^
				PACE		PACE		PACE		PACE	
				Terra^#^, Aqua^#^		Terra^#^, Aqua^#^		Terra^#^, Aqua^#^		Terra^#^, Aqua^#^	
				Suomi NPP^#^		Suomi NPP^#^		Suomi NPP^#^		Suomi NPP^#^	
				NOAA 20, 21		NOAA 20, 21		NOAA 20, 21		NOAA 20, 21	
				METOP-SG A1		METOP-SG A1		METOP-SG A1		METOP-SG A1	
**2025-2030**	Landsat 9, 10	Landsat 9, 10		Landsat 9, 10		Landsat 9, 10	Sentinel-4-series^#^	Landsat 9, 10	Sentinel-4-series^#^	Landsat 9, 10	Sentinel-4-series^#^
	Sentinel-1 series^#^	Sentinel-1 series^#^		Sentinel-1 series^#^		Sentinel-1 series^#^		Sentinel-1 series^#^		Sentinel-1 series^#^	
	Sentinel-2 series^#^	Sentinel-2 series^#^		Sentinel-2 series^#^	Sentinel-6	Sentinel-2 series^#^	Sentinel-6-series	Sentinel-2 series^#^	Sentinel-6-series	Sentinel-2 series^#^	Sentinel-6-series
				Sentinel-3 series*		Sentinel-3 series*	Sentinel-7, CO_2_M	Sentinel-3 series*	Sentinel-7, CO_2_M	Sentinel-3 series*	Sentinel-7, CO_2_M
						Sentinel-5-series		Sentinel-5-series		Sentinel-5-series	
	Sentinel-8, LSTM**	Sentinel-8, LSTM**		Sentinel-8, LSTM**		Sentinel-8, LSTM**		Sentinel-8, LSTM**		Sentinel-8, LSTM**	
	PRISMA^$#^	PRISMA^$#^		PRISMA^$#^							
	EnMAP^$#^	EnMAP^$#^									
				EE-7, BIOMASS$		EE-7, BIOMASS$		EE-7, Biomass$			
				EE-8, FLEX^#^		EE-8, FLEX^#^		EE-8, FLEX^#^			
			ISS/EMIT^$^	S-5P TROPOMI#	ISS/EMIT^$^	S-5P TROPOMI		S-5P TROPOMI		S-5P TROPOMI	
	SBG**	SBG**		SBG**		SBG**	GeoCARB	SBG**	GeoCARB	SBG**	GeoCARB
	Sentinel-10, CHIME**	Sentinel-10, CHIME**		Sentinel-10, CHIME**		Sentinel-10, CHIME**	Himawari 8, -9	Sentinel-10, CHIME**	Himawari 8, -9	Sentinel-10, CHIME**	Himawari 8, -9
		NISAR		NISAR		NISAR	GOES-18, 19	NISAR	GOES-18, 19	NISAR	GOES-18, 19
				PACE		PACE	MTG-I A,B^&^	PACE	MTG-I A,B^&^	PACE	MTG-I A,B^&^
				NOAA-20 series		NOAA-20 series		NOAA-20 series		NOAA-20 series	
				METOP-SG-I A,B		METOP-SG-I A,B		METOP-SG-I A,B		METOP-SG-I A,B	
Beyond 2030	Landsat tbd**	Landsat tbd**		Landsat tbd**		Landsat tbd**		Landsat tbd**		Landsat tbd**	
	Sentinel-1 series^#^	Sentinel-1 series^#^		Sentinel-1 series^#^		Sentinel-1 series^#^	Sentinel-4-series*	Sentinel-1 series^#^	Sentinel-4-series*	Sentinel-1 series^#^	Sentinel-4-series*
	Sentinel-2 series^#^	Sentinel-2 series^#^		Sentinel-2 series^#^		Sentinel-2 series^#^		Sentinel-2 series^#^		Sentinel-2 series^#^	
				Sentinel-3 series*	Sentinel-6-series*	Sentinel-3 series*	Sentinel-6-series*	Sentinel-3 series*	Sentinel-6-series*	Sentinel-3 series*	Sentinel-6-series*
				Sentinel-5-series*		Sentinel-5-series*		Sentinel-5-series*		Sentinel-5-series*	
	Sentinel-8, LSTM**	Sentinel-8, LSTM**		Sentinel-8, LSTM**		Sentinel-8, LSTM**	Sentinel-7, CO_2_M*	Sentinel-8, LSTM**	Sentinel-7, CO2M*	Sentinel-8, LSTM**	Sentinel-7, CO2M*
					GeoCARB*^#^		GeoCARB*^#^	EE-7, BIOMASS$	GeoCARB*^#^		GeoCARB*^#^
							Himawari-series	EE-8, FLEX#	Himawari-series		Himawari-series
				Sentinel-5P TROPOMI^#^		Sentinel-5P TROPOMI^#^	GOES-19-series	Sentinel-5P TROPOMI^#^	GOES-19-series	Sentinel-5P TROPOMI^#^	GOES-19-series
				NISAR^#^		NISAR^#^	MTG-series	NISAR^#^	MTG-series	NISAR^#^	MTG-series
				PACE*		PACE*		PACE*		PACE*	
	SBG**	SBG**		SBG**		SBG**		SBG**		SBG**	
	Sentinel-10, CHIME**	Sentinel-10, CHIME**		Sentinel-10, CHIME**		Sentinel-10, CHIME**		Sentinel-10, CHIME**		Sentinel-10, CHIME**	
								NOAA-20 series*		NOAA-20 series*	
								METOP-SG series*		METOP-SG series*	

$ Sampling Mission: Limited Collections in space or time

# Continuation Uncertain

* Expected to be Available

** Anticipated Mission

blank entries are left in table to allow following an instrument across spatial scales

The draft SLI science requirements for Landsat-next also envision an additional aspirational band set that would add 8–10 additional bands that are under consideration, bringing the total to 24–26 bands. If approved, fourteen of these bands will have higher GSD than the traditional 30 m resolution of all previous Landsats: four bands would have 10 m GSD (the blue, green, red, and new broad NIR band), and the remaining VSWIR bands would have 20 m spatial resolution. These higher spatial resolution bands are justified to support improved mapping capabilities for crops, minerals, snow/ice, and water resources, vegetation condition, and vegetation type discrimination. Plans for the Landsat-Next concept represent a significant evolution toward enhanced capabilities that facilitate integration of its high-quality global data with multiple space-based international instruments.

## Beginning

### Multispectral imaging satellites of NASA’s Earth Observation System (EOS and JPSS)

**Fig. 4:**
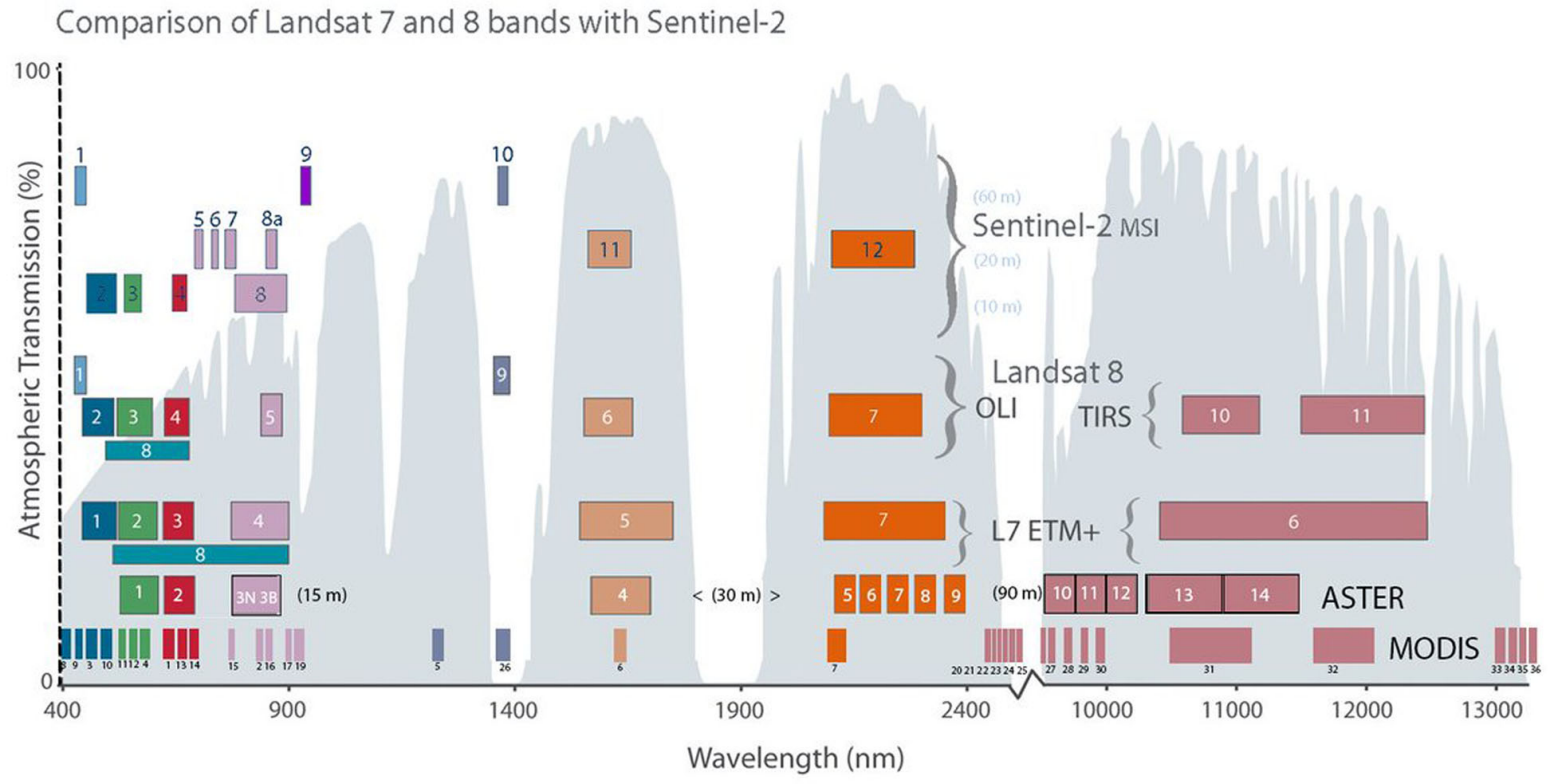
Comparison of Landsat-7, Landsat-8, and Landsat-9 bands with Sentinel-2, ASTER, and MODIS bands. Band widths, wavelength locations, and spectral region: VIS—blue, green, and red, NIR—lavender; SWIR 1— earth orange and SWIR—2 brick red; rose-colored bands are TIR. The band numbers for MODIS bands (*Bottom Row 1*) are shown in order of wavelength, in colors matching the bands: 8, 9, 3, 10—blue; 11, 12, 4—green; 1, 13, 14—red; 15, 2, 16, 17, 19—lavender; 5, 26—light blue; 6—earth orange; 7—brick red; 20, 21, 22, 23, 24, 25, 27, 28, 29, 30, 31, 32, 33, 34, 35, 36—rose. The narrow band 18 (931–941 nm) is not represented in this figure because it is within the wavelength range of band 19 (915–965 nm). Band 18 is located in the atmospheric water vapor absorption feature and contributes to the MODIS Precipitable Water Product. ASTER’s 14 bands (*Row 2*) range in size from 15 to 90 m, as shown. Landsat-7 and Landsat-8 (*Rows 3 and 4*) optical bands have 30 m pixels and thermal bands with 120 m (L-7) or 100 m (L-8). Sentinel-2 (*Top Row 5*) has 13 optical bands at either 10 m, 20 m, or 60 m spatial resolution and no TIR bands. Figure from https://twitter.com/usgslandsat/status/773939936755982336

NASA’s Earth Observing System (EOS) is a coordinated series of polar-orbiting and low inclination satellites for long-term global observations of the land surface, biosphere, solid Earth, atmosphere, and oceans. This program was conceived in the 1980s and began to take shape in the late 1990s, leading to core parts of NASA’s satellite fleet today. The subset of EOS missions focused on Earth observations has enabled understanding the Earth as an integrated system with focus on land surface ecosystem processes and hydrology, oceans, glaciers and ice, and characterization of many aspects of atmospheric components (Fig. 4). The EOS Project Office (in the Science Mission Directorate) brings information and resources to the science research community and the general public [www.eospso.nasa.gov].

The first EOS platform Terra was launched in late 1999, primarily for morning observations over land carrying five instruments and followed by Aqua in 2002 for afternoon observations primarily over oceans with six instruments. Both have sun-synchronous, near-polar LEO circular orbits providing global coverage every 1 to 2 days at variable viewing angles. Terra has a descending (during daylight) orbit with an equatorial overpass time of 10:30, while Aqua has an ascending (daylight) orbit that passes the equator at 13:30. Both Terra and Aqua were originally developed for a 6-year design life and both have greatly exceeded this with Terra in its 20th year and Aqua in its 18th year of successful operations. Of primary interest for environmental applications are the MODIS instruments on both platforms and the ASTER instrument on Terra. All three of these instruments leave a ~ 20-year legacy of Earth observation that is available at no cost from the NASA Land Processes Distributed Active Archive (LP DAAC), located at the USGS EROS Data Center in Sioux Falls, South Dakota, USA. The wavelengths and bandwidths for the spectral bands on MODIS and ASTER instruments on Terra, Landsat-8 OLI, and Sentinel-2 MSI are compared, with band colors indicating specific spectral regions in Fig. 3.

Four other notable Earth observation satellites (Fig. 4) of interest to ecologists include the SMAP (Soil Moisture Active Passive) satellite, a 2015 mission with an active radar (no longer functioning), but the passive radiometer continues to function flawlessly after 5 years (of a 3-year mission) to map soil moisture (every 2–3 days with 38 × 49 km pixels). The Visible Infrared Imaging Radiometer Suite (VIIRS) carries the MODIS mission into the 2030s, first on the NASA/NOAA Suomi NPP (National Polar-Orbiting Partnership) bridging mission, launched in 2011 and on the NOAA 20 JPSS series satellite in 2017 (see VIIRS below). The OCO-2 (Orbiting Carbon Observatory-2) mission has mapped atmospheric CO_2_ concentrations since 2014, which is improving understanding of sources and sinks of carbon at regional scales (> 1000 km) and across seasonal cycles. Lastly, the Global Precipitation Measurement (GPM) program is an international constellation of satellites (NASA and JAXA, the Japanese Aerospace Exploration Agency, launched the core satellite in February 2014) to observe near global patterns (from 65° N to 65° S), and intensities of rain and snow, providing timely information on disasters like floods, droughts, and landslides.

### Moderate Resolution Imaging Spectroradiometer (MODIS)

MODIS is the high-temporal and moderate spatial resolution polar-orbiting and sun-synchronous LEO imaging system on both Terra and Aqua that collects global data on a near-daily basis (Table 2) because its swath width of 2330 km, about 12.5 times wider than Landsat’s. MODIS has 36 bands from VIS through TIR (Fig. 3). Spatial resolution ranges from 250 m pixels for the red and NIR bands (bands 1 and 2), 500 m pixels for the five MODIS bands similar to those on L-4 to L-9 (MODIS bands 3, 4, 5, 6, 7), and the rest have 1000 m pixels, with bands 31 and 32 together approximating the TIR bandwidth on L-6 and L-7.

The MODIS land products are produced in three projections with the sinusoidal projection being the most common for land products. They are produced in adjacent non-overlapping tiles that are approximately ~ 10° square and are available from the NASA Land Processes Distributed Active Archive Center (LP DAAC), located at the US EROS Data Center (https://lpdaac.usgs.gov/), and at the NASA Goddard Space Flight Center MODIS web site (https://modis.gsfc.nasa.gov/data/).

**Fig. 5:**
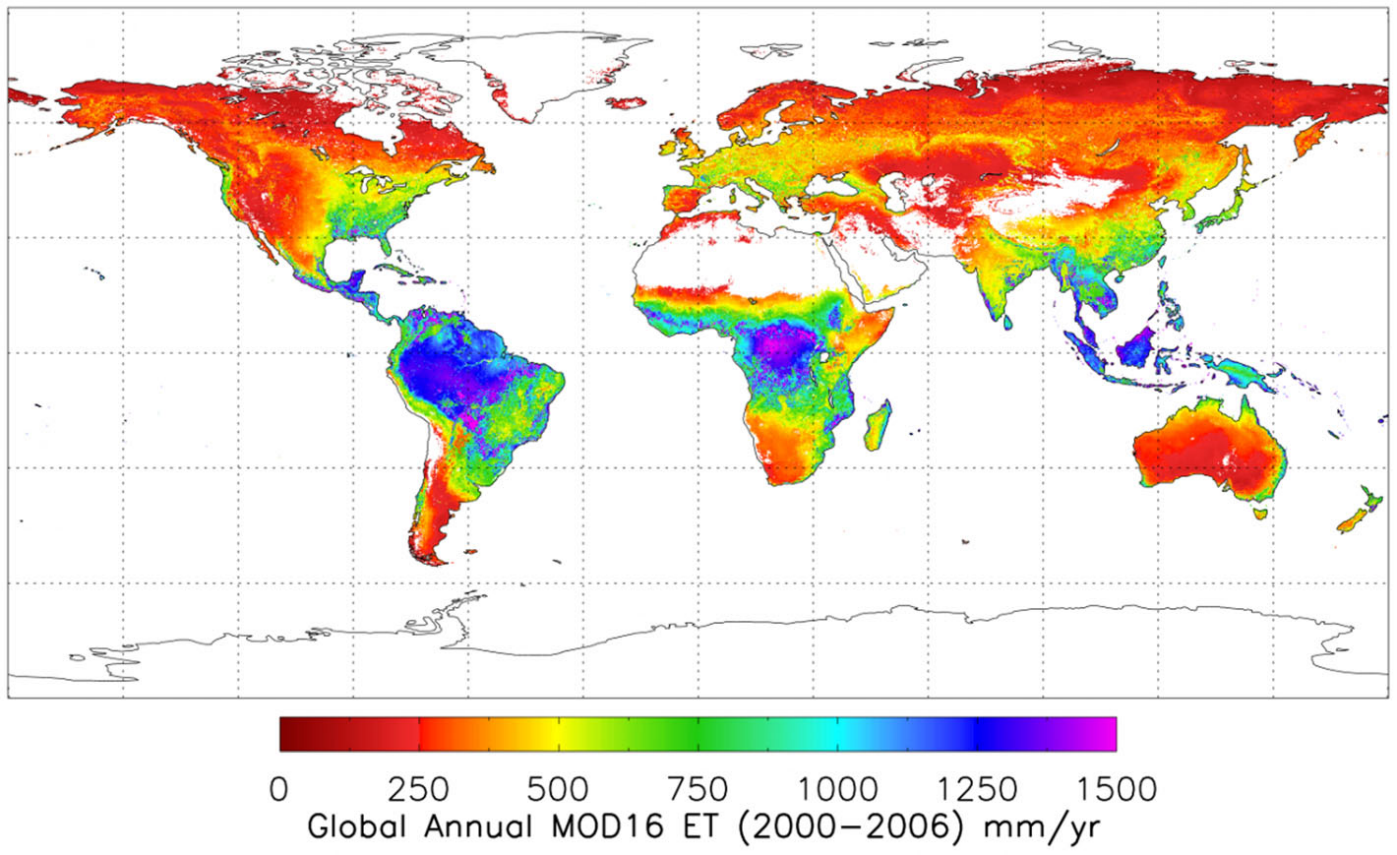
Example of the MOD16 evapotranspiration (ET) product, showing the average annual ET for a 7-year period (from https://www.ntsg.umt.edu/images/modis/GMAO_CMGalbedo_0.05deg_GEO.png)

The MODIS data products cover a wide range of science and application topics, and the number of resulting publications (24,074, 07/12/20) is nearly that of Landsat publications. MODIS data have transformed the ability to capture dynamic processes in the Earth system on land and ocean surfaces and in the atmosphere. Most global maps of seasonal vegetation patterns are based on the MODIS Normalized Difference Vegetation Index (NDVI) and/or the Enhanced Vegetation Index (EVI), from which many other vegetation parameters are estimated. Among many other applications in the last two decades, notable examples include biome distributions (e.g., Bonan 2008), vegetation cover types (Cohen et al. 2006), dynamic land cover (Friedl et al. 2002), fractional tree cover (DeFries et al. 1999), Leaf Area Index (LAI), the Fraction of Photosynthetically Active Radiation (FPAR; Myneni et al. 2015), Gross Primary Productivity (GPP), and Net Primary Productivity (NPP) (Zhao et al. 2005). The MODIS standard Global Evapotranspiration product MOD16 provides global evapotranspiration (ET; Fig. 5), latent heat flux (LE), potential ET (PET), and potential LE (PLE), originally processed using a modified Penman-Monteith model (Mu et al. 2007, 2011) as described for the MOD16 product for the time period 2000–2010 (http://www.ntsg.umt.edu/project/modis/ mod16.php). Anderson et al. (2007) used a Priestley-Taylor approach in the ALEXI model to estimate continental scale ET computed from MODIS data.

### Advanced Spaceborne Thermal Emission and Reflection Radiometer (ASTER)

ASTER is a multispectral imaging sensor, launched in 1999 on the EOS Terra platform and built by Japan’s Ministry of International Trade and Industry (METI); it is jointly managed by METI and NASA. All ASTER data are free and available at the LP DAAC site (lpdaac.usgs.gov). ASTER has a 60-km swath and 15 bands, four VNIR at 15 m pixels (2 VIS and 2 NIR, 1 nadir, 1 backward looking), 6 narrow SWIR bands with 30 m pixels, and 5 TIR bands with 90 m pixels, with placements designed specifically for geological/mineralogical research (Table 2, Fig. 3). They are, however, also useful for vegetation and soil properties. Unfortunately, the SWIR bands failed in 2008, but the VNIR and TIR remain fully functional. ASTER was designed to be a sampling instrument, collecting a limited 8-min along-track dataset each orbit. It has two telescopes, one nadir looking (down) and one backward looking with a single band detector, which together collect stereo imagery for resolving vertical height. Additionally, the entire telescope system can be rotated so it is pointable side-to-side in the cross-track direction, providing additional stereo data and increasing the potential of observing a particular land feature from adjacent orbits as well as the nadir orbit. Despite its limited collections, the stereo feature allowed ASTER to collect stereo imagery many times nearly everywhere on earth until a global dataset of 2.3 M scenes was produced and used to create a 30 m Global Digital Elevation Model (DEM) (version 3, 2019). The DEM covers the land surface from 83° N to 83° S with 7 m vertical resolution (Tachikawa et al. 2011; Abrams et al. 2015). This DEM is available free from NASA and METI (at the LP DAAC (https://lpdaac.usgs.gov/news/lp-daac-offers-aster-digital-elevation-model-dem-products-using-new-production-software/).

ASTER’s primary mission is geomorphology and mineral exploration (Rowan and Mars 2003; Di Tommaso and Rubenstein 2007), but the data are good for general land cover mapping due to its VNIR bands at 15 m pixel resolution and SWIR bands at 30 m (Fig. 3, Table 2). ASTER enabled similar studies to those in the Landsat literature on forest fuels (Falkowski et al. 2005), fractional cover bare ground (Gill and Phinn 2008), urban mapping (Pu et al. 2008; Chen et al. 2007), heat islands (Weng et al. 2011), and landslide susceptibility (Choi et al. 2012), wildfire detection (Giglio et al. 2008), and bare ground and green vegetation fractions (Gill and Phinn 2008). The SWIR bands were useful for identifying many geologic minerals (e.g., hematite, goethite, pyrite, olivine, quartz, and carbonate (Rockwell and Hofstra 2008) and soil components, like clays (e.g., kaolinite, alunite, montmorillonite), organic matter and dry plant residues (Hunt 1977; Daughtry et al. 2004), and soil moisture (Lobell and Asner 2002). The five TIR bands are sufficient to separate emissivity and kinetic temperature (Gillespie et al. 1998). Hulley et al. (2015) created a global dataset of emissivity at 100 m resolution available at NASA LP DAAC at https://lpdaac.usgs.gov/news/aster-global-emissivity-dataset-ged-product-release/. TIR bands are less frequently used for land cover mapping but are important in detecting thermal-induced vegetation stress (Gerhards et al. 2019), extreme hot or cold temperatures (Carter et al. 2008), heat island effects (Roberts et al. 2012), and temperatures of glaciers and water bodies (Wessels et al. 2002; Hellman and Ramsey 2004), and are used to estimate evapotranspiration (Galleguillos et al. 2011; Hoedjes et al. 2008b; Li et al., 2017).

### NASA-NOAA’s Visible Infrared Imaging Radiometer Suite (VIIRS)

The success of the MODIS instruments led to development of an operational global instrument, VIIRS, first flown on the prototype Suomi NPP mission in 2011 that was named for Verner E. Suomi, a meteorologist recognized for establishing satellite meteorology. Suomi NPP carries four instruments, all of which (including VIIRS) trace their heritage to the NASA Terra flagship. The Suomi NPP mission bridged the gap between the EOS-era instruments and the next-generation of polar orbiting weather satellites in the Joint Polar Satellite System (JPSS) Program (NOAA-NASA) which formally began with the launch of the NOAA-20 polar orbiting weather satellite in 2017.

Suomi NPP was launched into a 13:30 afternoon Equatorial crossing time LEO carrying VIIRS, extending the MODIS record on Aqua (afternoon orbit). Also, VIIRS continues NOAA’s imagery of the Earth’s surface from their earliest land imager, the Advanced Very High Resolution Radiometer (AVHRR). A VIIRS instrument (JPSS-1) became the first in its series of operational instruments in the JPSS series, which are the primary polar-orbiting US weather satellites for the next two to three decades (JPSS 2014). Note that JPSS-1 became NOAA-20 after launch and has a 12:40 Equatorial crossing time. The VIIRS instrument has a 3060-km swath to attain global daily coverage at varying view angles (Table 2). It has 22 spectral bands, five with high spatial resolution (375 m pixels) imagery bands (I-bands), and 16 moderate spatial resolution VIS through TIR bands (M-bands, 750 m pixels), providing 9–50 times better spatial resolution than MODIS. The panchromatic day/night band (allows data to be collected day and night) also has 750 m pixels.

**Fig. 6:**
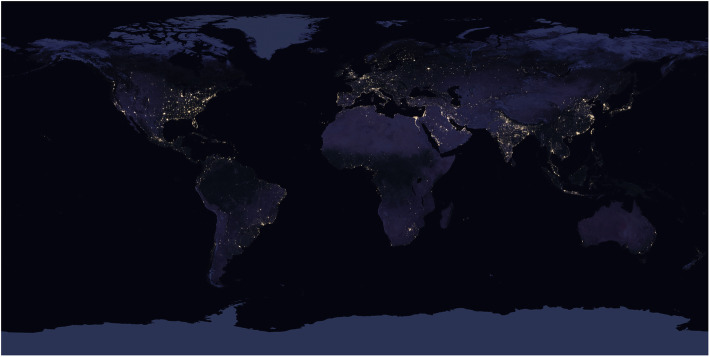
A VIIRS 2016 Black Marble 3 km composite image of global night lights, created from the NASA VIIRS DNB (Day-Night Band) algorithm (resolution reduced from https://viirsland.gsfc.nasa.gov/images/BlackMarble_2016_3km.jpg)

VIIRS bands provide data for regional to continental and global studies of vegetation phenology patterns (Zhang et al. 2018; Moon et al. 2019), active fire detection (Schroeder et al. 2014), burned area mapping (Olivia and Schroeder 2015), and albedo (Liang et al. 2013). The day-night band is used for detecting small fires and urban lights (Fig. 6) that is similar to the band on the Defense Meteorological Satellite Program-Operational Line Scanner (DMSP-OLS). It is sensitive to very low light levels, millions of times lower than reflected sunlight. The DMSP-OLS has coarser spatial resolution but has successfully mapped electricity consumption based on nighttime light intensity (Elvidge et al. 1997; Janinski 2019), population distributions (Gao et al. 2019), and economic needs such as estimating storm damage (Cao et al. 2013). The VIIRS design also adopted the method used on DMSP-OLS to reduce the size distortion of pixels toward the edge of the swath, based on a method of constrained off-nadir pixel growth, that produces near constant spatial resolution across and along track for all 22 bands (Schueler et al. 2013).

An early evaluation of VIIRS vegetation indices (NDVI and EVI) show their measurements are consistent with MODIS (Vargas et al. 2013), thereby supporting the potential to extend the long-term terrestrial data record. Despite much better spatial resolution than MODIS for many applications, a large number of environmental/ecological applications using these data are not yet into the literature. Nevertheless, the VIIRS instrument onboard Suomi NPP and NOAA-20 and subsequent ones flying over the next 20–30 years should be of significant interest for ecosystem measurements, and for extending time series data linked to Aqua and Terra. These advances in global monitoring with multiband imagers by NASA and NOAA have compelled other international space agencies to plan and implement similar missions.

### ICESat-2

Another mission that deserves mention for the biomass community is NASA’s ICESat-2 (Ice, Cloud, and land Elevation Satellite 2), launched in September 2018 into a polar orbit at ~ 500 km (Table 2). ICESat-2 is a 3-year satellite mission (with enough fuel for 7 years) to measure ice sheet height, sea ice thickness, and land topography, including vegetation height characteristics and clouds. ICESat-2’s sole instrument is the Advanced Topographic Laser Altimeter System (ATLAS), a space-based multi-beam LiDAR system that emits laser pulses from three pairs of lasers at a wavelength of 532 nm. ATLAS measures the two-way travel times from satellite to Earth for these photon pulses, which are used to determine elevation. Elevation measurements are made at 1 km spatial resolution and enable determination of vegetation height to 3-m accuracy (Herzfeld et al. 2014). These vegetation canopy height measurements are then utilized for estimating large-scale biomass and biomass change.

### Commercial satellites

Commercial satellites are relevant to ecosystem research, despite cost, because of their high spatial resolution data (commonly < 5 m). Such data can be used to improve maps from coarser satellite data (e.g., to train classifiers) or to validate results and interpretations. Although much of the orbital imagery collected by “for-profit” commercial entities are not typically “free and open,” we mention them here because much of their data are available on public access websites, especially if older than 5 years. They are also worth mentioning because they provide high temporal frequency data globally that are suitable for building time series maps, and because they are pushing the envelope for new data types from space.

The first commercial satellite, SPOT 1 (Satellite Pour l’Observation de la Terre), was launched in 1986, less than 2 years after Landsat-5 was commercialized. The SPOT satellites have been a commercial collaboration with the French Centre national d'études spatiales (CNES). SPOTs 1–4 offer a choice between operating as a panchromatic band at 10 m pixel resolution or as a 3-band (Green, Red, NIR) multispectral sensor with 20 m pixels, both types were acquired across a 60-km swath. Several of the commercial satellites (WorldView 1–4, Quickbird, GeoEye, and Ikonos-1 and Ikonos-2) have high spatial resolution (1–5 m) that were previously managed by Digital Globe, but are now incorporated into Maxar Technologies. These and other commercial satellites with similar high spatial resolution have most often been limited to 3–4 VNIR bands, with some including a panchromatic band at even higher spatial resolution. More recent small satellites, such as WorldView 4 (failed in 2019), had 31 cm resolution for panchromatic data and 1.24 m resolution for VNIR multispectral data, plus 8 SWIR bands and 12 bands to detect clouds, aerosols, water, and snow (known as CAVIS bands). Planet Lab has the largest fleet of 21 CubeSats (as of 8/2020), referred to as Doves, that acquire sub-meter resolution data. This SkySat constellation fleet can image any point on Earth at 50 cm resolution up to twice daily, the highest revisit provided from any commercial high-resolution imager. Planet Lab also manages the 5-CubeSat constellation of RapidEye, each satellite has identical 5 VNIR bands with 6.5 m resolution.

In many cases, the commercial entities for these satellites have changed over time and their data, or some of their data, have been included in public databases. Airbus, the corporate owner of SPOT satellites, has quit processing SPOT 1–5 data, but which are available (along with newer SPOT 6 and 7 data) at the Theia Land Data Center (https://www.theia-land.fr/en/satellite-data/), providing free availability of orthorectified products for non-commercial use (often for limited areas or times, primarily Europe and Africa), derived from multispectral data from the SPOT 1–7 satellite family (Table 4). Also posted there are other commercial (e.g., RapidEye/Satellite Imaging Corp.) and government or research satellite data from Landsats 7–9 (Table 2), ASTER (Table 2), Sentinel-2 (Table 3), Venμs, and Pléiades (Table 4). Another large source of data is from ESA’s GEOSS Portal from the Group on Earth Observations (www.geoportal.org). In the USA, the US Geological Service’s EROS Data Center (www.usgs.gov/products) provides an extensive database of historic data, including SPOT 1–5 images for the America’s and GeoEye’s OrbView data, and a range of other data types from multiple sources, including CBERS, THEOS, and ERS-1 and ERS-2. The USGS/NASA Land Processes Data Active Archive Center (LP DAAC; https://lpdaac.usgs.gov/) is another source of various satellite datasets (discussed in later sections), and at the NASA Goddard Space Flight Center data archives (https://modis.gsfc.nasa.gov/data/). Another source for free and open access (although with registration, proposal, and non-commercial use) is ESA’s Web Client Catalogue (eocat.esa.int/sec/#data-services-area) that includes SPOTs 1–7 (archived and new), SPOTMaps, and Pléiades (see also Table 4), PROBA CHRIS, RapidEye, SeaSat, SMOS, ALOS, POLDER, JERS-1, and the WorldView satellites 2, 3, and 4.

## Europe’s Earth Observation Program

### Copernicus and the European Space Agency

**Fig. 7:**
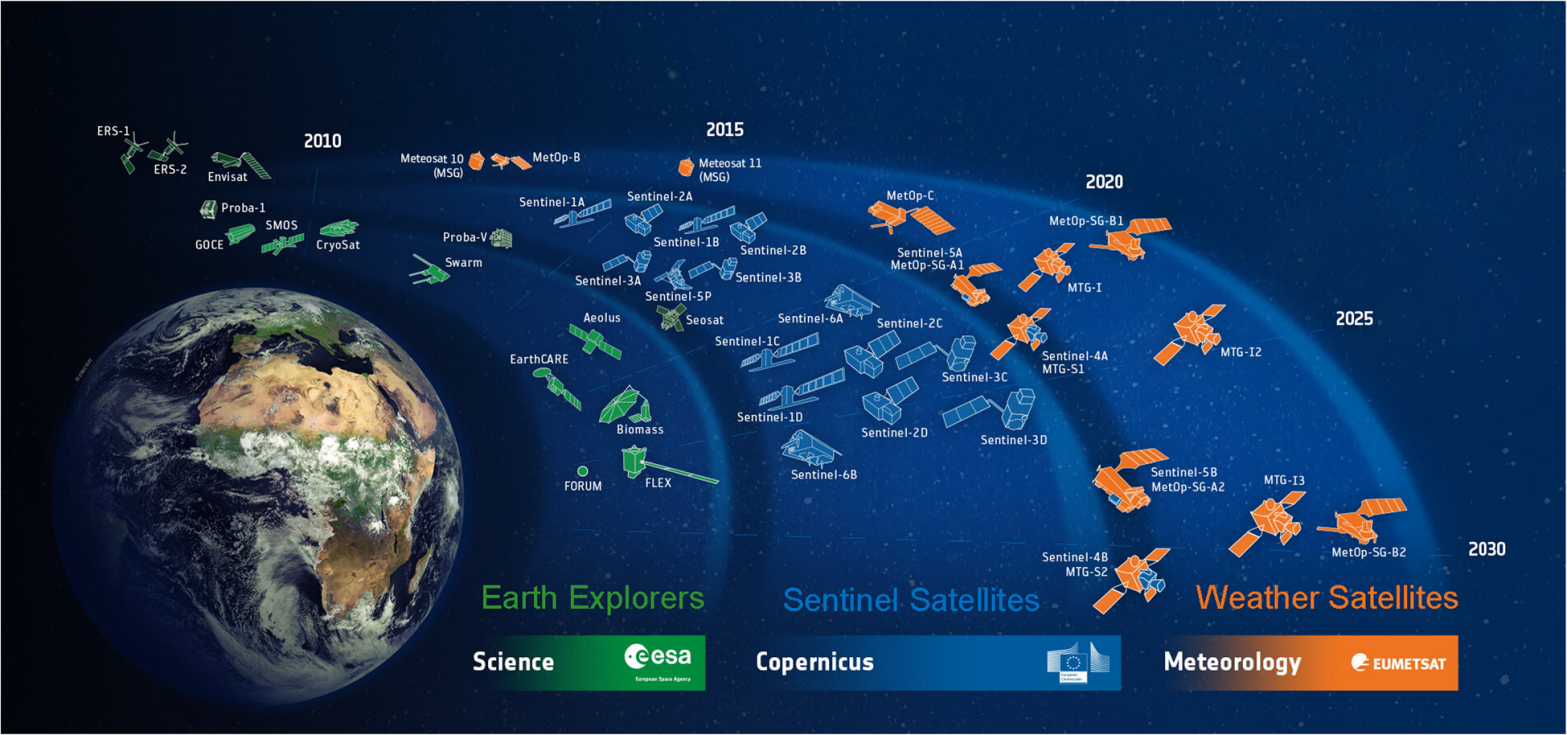
The European Space Agency’s program in Earth observation from 2010 to 2030. The period from 2020 to 2025 is especially active, launching several science demonstration missions (green, the Earth Explorers), a large number of Copernicus satellites, the Sentinel operational mission series of Earth observers, and the Operational Meteorology program operated by EUMETSAT, with several Sentinel missions in partnership with ESA

The European Union’s (EU) Copernicus “Europe’s eyes on Earth” Program (Fig. 7), formally initiated in 2012 in coordination with the European Space Agency (ESA), develops and manages satellites addressing scientific needs for land, air, and sea. ESA operates the Sentinel program (https://www.esa.int/Applications/Observing_the_Earth/Copernicus/Overview3) as well as the Earth Explorer series (currently EE-1 through EE-9). The European Organization for the Exploitation of Meteorological Satellites (EUMETSAT) is the agency responsible for the operational meteorological satellite programs, as well as the marine program under the Sentinel-3’s mission, and will operate and deliver products from Sentinel-4, Sentinel-5, and Sentinel-6 satellites. The EU introduced a full, free, and open data policy in 2013, after the successful demonstration of the 2011 US initiative.

ESA operated two of the EU’s earlier Earth satellites: the Earth Remote Sensing (ERS-1 and ERS-2) radar satellites flown from 1991 to 2011, and the 10 instruments on the Environmental Satellite (EnviSat) that was operated for a decade, 2002–2012. EnviSat’s most important land observation instrument was MERIS, the programmable MEdium Resolution Imaging Spectrometer, a MODIS-like VIS/NIR imager that allowed the user to define fifteen spectral bands (by wavelength and bandwidth); observations were acquired at nadir over a wide 1150-km swath that enabled a 3-day repeat cycle. A second important instrument, and also the smallest ESA land imager, launched in 2001 as a 2-year demonstration and later converted to operational use and is still operating: the Compact High Resolution Imaging Spectrometer (CHRIS) onboard the PROBA-1 (PRoject for On-Board Autonomy-1) CubeSat. CHRIS acquires 17–34 m pixels for 13-km^2^ scenes for 18 user-selected VIS/NIR wavelengths (from 63 possible) for measurements acquired from up to five different viewing angles. Multiple view angles produce slightly different images of the surface. When combined, they provide information about 3-D surface structure. These two instruments are precursors for today’s European hyperspectral satellites PRISMA and EnMap, and the proposed operational hyperspectral satellite CHIME, described below.

### The Sentinels

The Sentinel satellite constellation is the operational component of Copernicus, the EU’s Earth Observation Program. ESA has committed to support several types of polar-orbiting Sentinels that are currently in orbit—Sentinel-1, Sentinel-2, Sentinel-3 (for the mission’s land component), and Sentinel-5P, at least until 2030, and Sentinel-4, Sentinel-5, and Sentinel-6 are in development. Each Sentinel has at least one satellite in orbit at a time, with duplicates launched at staggered times to ensure continuity and to increase observation frequency. Multiple satellites in a series are designated with letters of the alphabet, such as Sentinel 2A and 2B (S-2A, S-2B), and Sentinel 3A, 3B, and 3C (S-3A, S-3B, S-3C). This nomenclature is used for S-1 through S-3, and for some future Sentinels. As they age, the oldest pair of a series (e.g., S-1A and S-1B) are planned to be replaced and upgraded by succeeding pairs (e.g., C-D and E-F, etc.), thus providing operational continuity over the next two decades.

In addition to the four Sentinel series currently in orbit, three more are in the pipeline for launch between 2021 and 2030, bringing the existing and planned suite of Earth Sentinels to seven “flagship” satellite types (Fig. 7): Sentinel-1 (radar), Sentinel-2 (passive multispectral), Sentinel-3 (passive multispectral, TIR, altimetry, and more), Sentinel-4 (atmospheric constituents from GEO), Sentinel-5P (atmospheric constituents from LEO), Sentinel-5 (atmospheric constituents from LEO), and Sentinel-6 (ocean altimetry from LEO). ESA is already preparing for Copernicus 2.0 with six additional high-priority instruments that expand the capabilities of the current generation, for the next high-priority science missions, described below.

#### Sentinel-1 (2A, 2B, and replacements 2C, 2D)

**Fig. 8:**
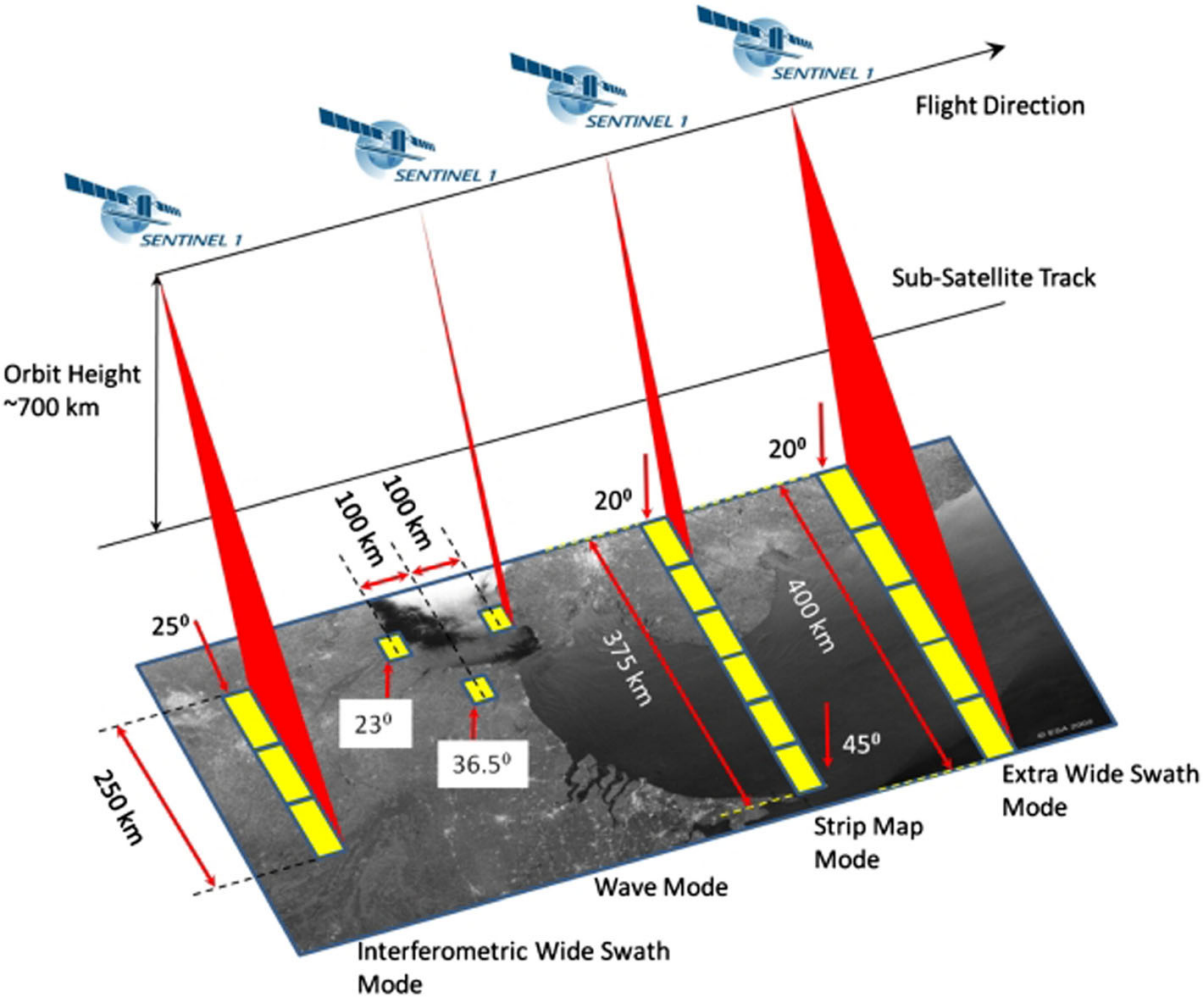
Four acquisition modes for Sentinel-1 (from https://sentinel.esa.int/web/sentinel/technical-guides/sentinel-1-sar/sar-instrument under the subheading Acquisition Modes). Radar satellites differ from optical sensors by their ability to shift between different acquisition modes to accomodate a larger suite of applications

The first satellite in the Sentinel suite is Sentinel-1 with a 7-year lifespan, composed of a pair of polar-orbiting (LEO) satellites (Sentinel-1A launched in 2013 and Sentinel-1B launched in 2016) that continue all-weather, day-and-night imaging with C-band (~5.4 GHz frequency distribution corresponding to 5.55 cm wavelength resolution) Synthetic Aperture Radar (SAR) (Table 3). This instrument builds on heritage from SAR missions like ERS-1, ERS-2, Envisat, and Canada’s RADARSAT. The S-1s collect data in four polarization orientations (VV, HH, VH, HV). This nomenclature indicates the radar pulse is released in a vertical polarization and received at the detector in a vertical polarization (VV) or horizontal (HH). Generally, VV provides a stronger return for objects like buildings and HH for ground surface. The polarization bands yield four types of images, and four viewing modes that have different swath widths and spatial resolutions (Fig. 8): Stripmap (SM) has an 80-km swath and 5 m × 5 m pixels; Interferometric Wide Swath (IW) a 250-km width with 5 m × 20 m pixels; Extra Wide Swath (EW) a 400-km swath with 25 m × 100 m pixels; and Wave (WV) with 20 km × 20 km blocks and 5 m × 20 m pixels. The SM mode is only operated on request for extraordinary circumstances to obtain continuous high spatial resolution observations. The IW mode was designed to generate interferograms and coherence maps, which are widely used to locate landscape changes between different dates of imagery, especially disasters like earthquakes, volcanic eruptions, and landslides, but ecological and land use changes are also measured, e.g., wildfires or clear-cut logging among others. The IW supports a new ScanSAR mode termed *Terrain Observation with Progressive Scan SAR* (*TOPSAR*) which achieves highly accurate, nearly uniform responses for image co-registration by reducing drawbacks that shrink the azimuth antenna pattern (along-track direction) as seen by a ground target. The EW mode also uses the TOPSAR imaging technique (De Zan and Guarnieri 2006) for acquiring five small strips (vignettes) of 100-km-long alternating intervals in the along-track direction to cover a very wide area at Landsat-scale ground resolution. In the WV mode, a SM image is acquired, and vignettes at the same incidence angle are separated by 200 km.

Applications utilizing S-1 data include land surface monitoring (forests, water, soil, and agriculture); maritime monitoring for coastal zones, iceberg, and sea ice; and mapping natural disasters including oil spills. C-band radar is thought to mostly detect scattering from the upper layer of vegetation canopies with little penetration of forests to the ground surface, so it is likely better for land cover and vegetation mapping than for mapping understory topography or vegetation height. At high latitudes, because of overlapping orbits near the poles that enable frequent acquisitions, the S-1s can observe changes in ice flows, ship detection, snow melt, snow water content, flooding, and other changes on a daily basis (Malenovsky et al. 2012). These datasets enable timeliness and reliability for operational services and applications that require time series data.

#### Sentinel-2 satellites (2A, 2B, and replacements 2C, 2D)

Sentinel-2s are expected to be the primary satellites of interest for environmental and ecological applications in the Copernicus program, with goals similar to the Landsat program (Table 3, Fig. 3). They form a pair of LEO polar-orbiting multispectral high-resolution imaging systems for land monitoring of vegetation, soil, and water, between 84° N and − 56° S latitudes. Sentinel-2 provides routine land cover mapping with improved spatial and spectral resolution bands relative to the L-8 OLI. Sentinel-2A was launched in 2015 and Sentinel-2B in 2017, both flown in the same sun-synchronous descending polar orbit as Landsat, with a similar equatorial overpass time (10:30) but with a wider swath of 290 km (vs. the Landsat’s 185 km), which, along with a lower orbit, enable each S-2 to have a 10-day repeat observation period, and when flown 180° apart, they achieve 5-day repeat coverage at the Equator and more frequent acquisitions at higher latitudes. The Sentinel-2 instrument is the multispectral imager (MSI) which has 13 spectral channels with differing spatial resolutions, including 10 image bands (Table 3) and 3 bands for calibration. Three new spectral bands measuring in the red edge of plant spectra (~ 0.7–0.8 μm) are unique to Sentinel-2 and have 20 m pixels. There are four bands with 10 m pixels (blue, green, red, and NIR) with similar placements to L-8’s OLI. Six bands are collected at 20 m pixels (in the red edge; NIR and SWIR), and three calibration bands at 60 m pixels, one each in the blue wavelengths for aerosols, NIR for water vapor, and SWIR for ice crystal cirrus cloud detection (Fig. 3). All data are reported with 12-bit radiometric resolution.

Given their improved spatial and spectral resolutions, the Sentinel-2 MSIs function like an enhanced Landsat, although lacking the thermal bands (Fig. 3). MSIs’ unique four narrow (~ 0.015–0.020 μm) “red edge” bands (Figs. 1 and 3) are key measurements for plant/ecosystem stress detection, and for estimating plant chlorophyll content (Clevers and Gitelson 2013; Schlemmer et al. 2013), leaf area index (Delegido et al. 2011, 2013), and nitrogen content (Clevers and Gitelson 2013; Schlemmer et al. 2013). The Copernicus satellite data are available from the ESA web sites or though the USGS EROS Data Center, and some are available “harmonized” with Landsat data (Claverie et al. 2018; Masek et al. 2018). This is done by resampling the spectral resolutions of S-2 bands (2, 3, 4, 8, 11, 12) to Landsat bands, normalization of BRDF effects to account for different solar and view angles (Masek et al. 2018), and then processed MSI images are gridded to match the 30 m spatial resolution of the Landsat OLI. For this special subset of combined Sentinel-2/Landsat-8 data, 5-day coverage is achieved; when L-9 is launched, a 2/3-day repeat coverage at the equator could be realized (and near daily in mid-latitudes). For the selected regions included in the combined harmonized datasets, this high-frequency monitoring of agriculture will increase the likelihood of obtaining timely data throughout a growing season, especially at critical developmental stages in a crop’s life cycle or when affected by environmental stresses (e.g., drought or disease). This unique effort demonstrates the value of a shared international prototype mission that could revolutionize dynamic monitoring in agriculture and ecology.

**Fig. 9:**
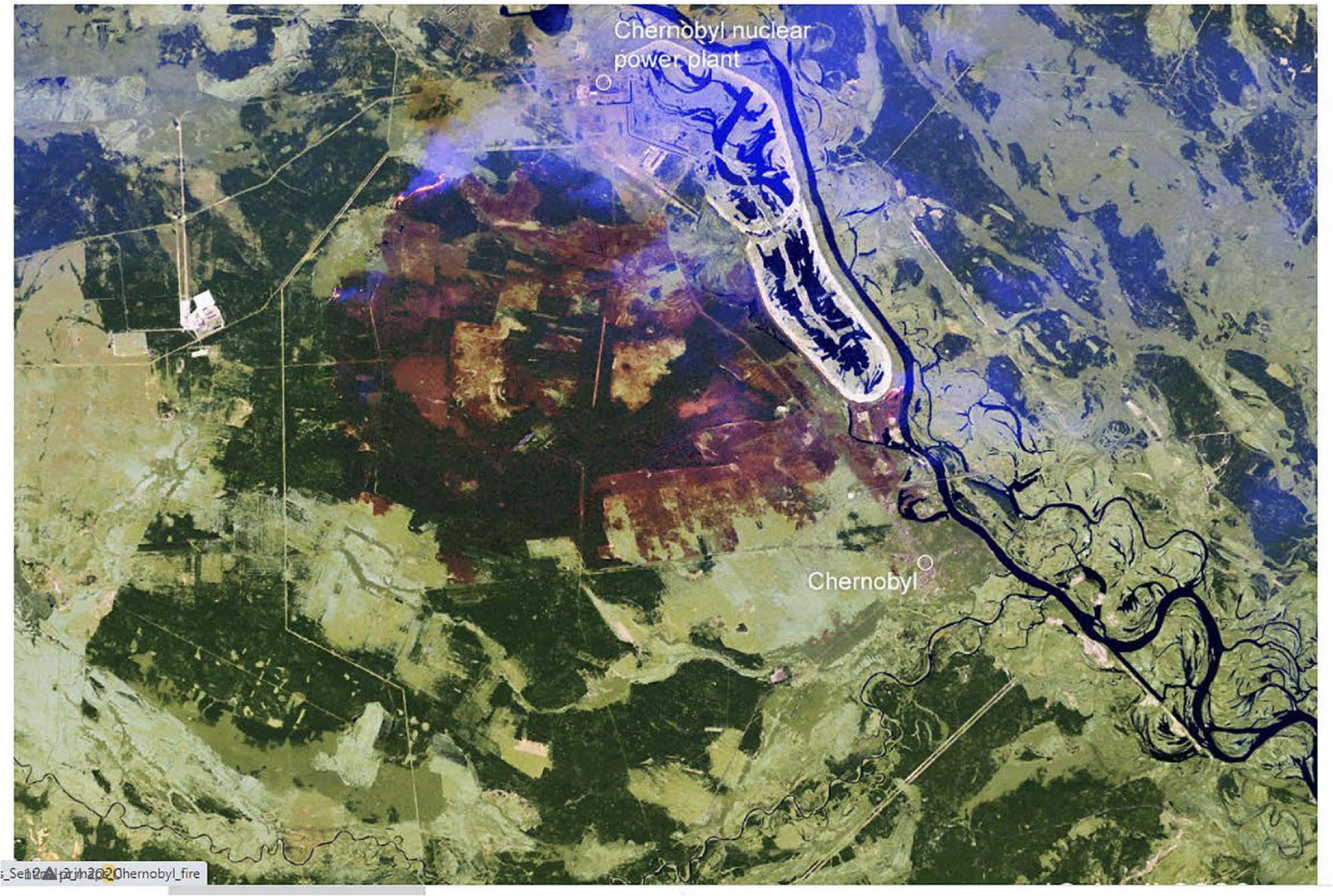
Sentinel-2 image with few active fire fronts remaining from the 7–12 April, 2020, fire within the 30 km diameter Exclusion Zone around the Chernobyl reactor site that had a catastrophic meltdown in 1986. The 13 spectral bands from S-2 data were processed by ESA to show the smoke plumes and the bright active fire areas along the front edges of the burned area. Smoke plumes are easily observed in the visible bands while the enhanced radiance of the active fire area is easily detected in the SWIR region. The burned area is often imaged from a normalized burn ratio (the difference in reflecctance between NIR-SWIR divided by the sum NIR + SWIR). Figure is based on analysis of Copernicus Sentinel-2 data (12/04/2020), processed by ESA under Creative Commons Attribution-ShareAlike 3.0 IGO (CC BY-SA 3.0 IGO) License)

Figure 9 illustrates the repeat coverage capability of S-2 satellites and some of its ecological information. The small town of Chernobyl is indicated to the southeast of the reactor site (Fig. 9); the larger town of Pripyat was located to the northeast and remains unoccupied. The reactor complex is seen on the upper left corner of the cooling pond, near the edge of the burned area. The fire burned an area (seen in this image as a reddish-brown color) close to the damaged reactor where a radioactive storage facility is still located. The pine forest that surrounded the plant in 1986 when the reactor meltdown occurred was heavily contaminated and crews removed and buried the trees on-site, also removing 10–15 cm of top soil. Vegetation surrounding the site today are patches of pine and broadleaf forests (dark green), interspersed with grasslands (light green) that are either former agricultural fields or contaminated soils. Despite the radiation, wildlife has returned to the region along with extensive vegetation regrowth. A few people are still living in the area since the accident.

The white area delineates the levee boundary of the reactor’s cooling pond, constructed with local sands (Bugai et al. 1997). The dike down the center of the lower pond area guided the hot water from the reactor down the left side, cooling as it moved around and up the right side of the pond before it was returned to the Pripyat River. The meandering Pripyat River running toward the southeast (which appears black) on the northeast side of the pond was the source of the cooling water.

#### Sentinel-3 satellites (S-3A and S-3B)

**Fig. 10:**
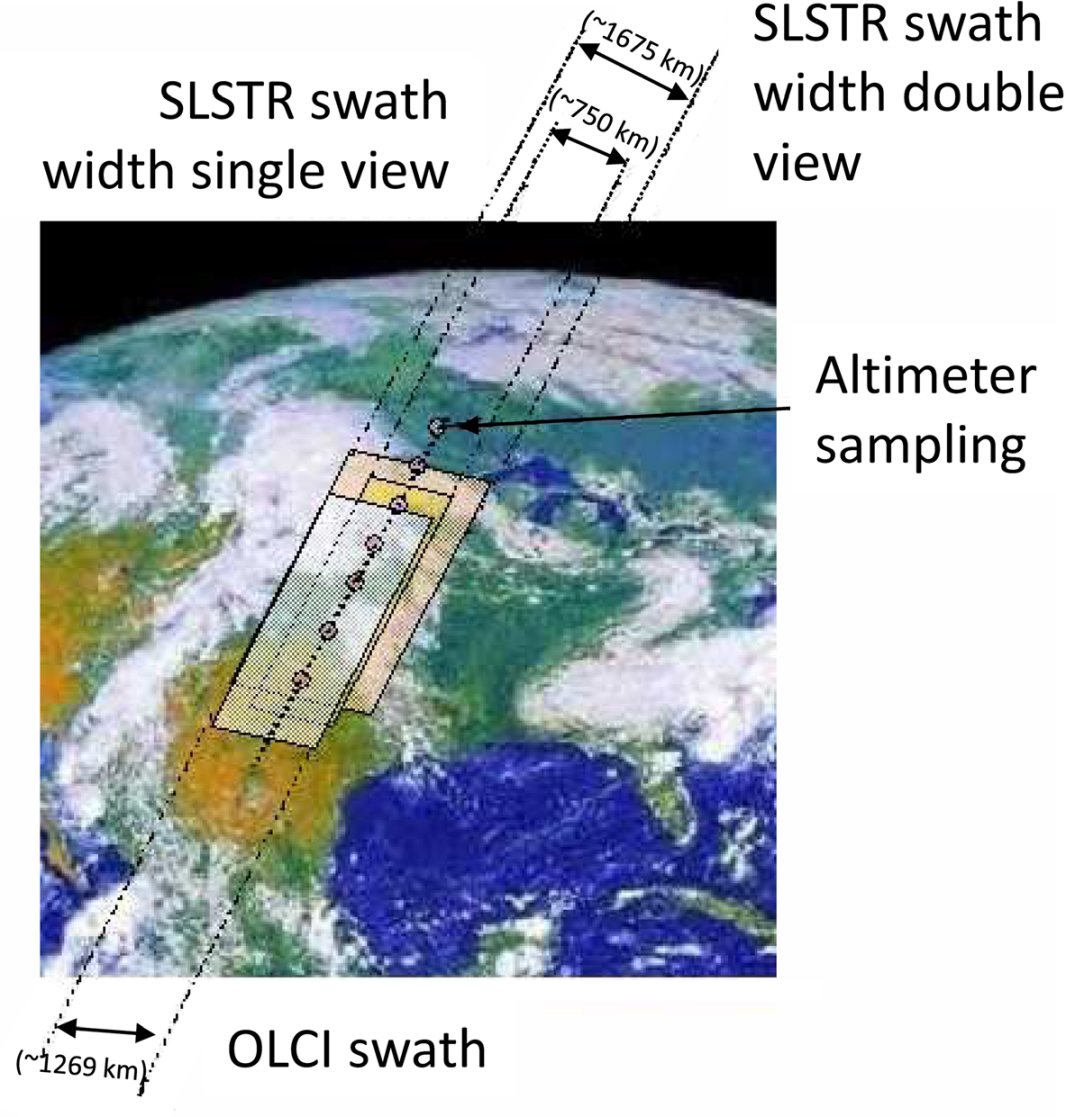
The Sentinel-3 ground track acquisition geometry showing co-registered segments of SLSTR and OLCI imaging sensors. Figure derived from https://sentinel.esa.int/web/sentinel/user-guides/sentinel-3-synergy/coverage

The S-3s carry identical 5-instrument packages in LEO orbit that are flown offset by 140° with a 10:00 equatorial crossing time and combined revisit frequency of 1–2 days (Table 3). The first S-3A was launched in 2016, and S-3B was launched in 2018. Of most interest to the ecological community is the Ocean and Land Colour Instrument (OLCI), with heritage to EnviSat/MERIS for measuring land and ocean reflectances at high accuracy and reliability for environmental and climate monitoring. OLCI has five camera modules for VIS/NIR images, and a swath width of 1200 km. The Sea and Land Surface Temperature Radiometer (SLSTR) has a swath width of 750 km, a track that falls within that of OLCI (Fig. 10) to provide concurrent measurements of land surface temperatures and land color with 300 m pixels. The S-3 satellites also have dual (near-nadir to reduce sunglint, plus backward looking) views that provide an improvement over MERIS for relatively high spatial resolution sea surface temperatures, data needed for modeling global ecosystem processes. Two bands were added for measuring fire hotspots over land, although all land products are currently secondary to production of ocean and marine observations. Land products will become a priority for Sentinel-3C which is planned for launch in 2022, and will partner with the FLuorescence EXplorer (FLEX) mission (Table 3), the Earth Explorer 8, in a tandem mission.

#### Sentinel-5 Precursor (S-5P) satellite

The S-5P is the prototype for the future Sentinel-5 and was launched in LEO polar orbit in 2017. S-5P hosts the TROPOMI (TROPOspheric Monitoring Instrument) with a swath of 2600 km with 7 km × 3.5 km pixels for VIS/NIR bands and 7 km × 7 km for SWIR bands. TROPOMI hosts a high-resolution spectrometer (Table 3) that was needed sooner than the Sentinel-4 scheduled launch (2023) into GEO and Sentinel-5 scheduled for LEO launch (2021), all three for satellite chemistry and weather observations. The S-5P mission is an effort to extend measurements of atmospheric chemistry from earlier satellites such as the Ozone Monitoring Instrument (OMI) on the EOS Aura platform, and the SCanning Imaging Absorption spectroMeter for Atmospheric CHartographY (SCIAMACHY) on EnviSat, while waiting for the launches of S-4 and S-5. TROPOMI monitors air pollution that affects air quality and climate, by providing data on ozone (O_3_), methane (CH_4_), carbon monoxide (CO), formaldehyde (HCOH), nitrous dioxide (NO_2_), sulfur dioxide (SO_2_), and aerosols. TROPOMI currently provides the most detailed monitoring capability for methane emissions available from space. Measurements are made with grating spectrometers, sensing ultraviolet (UV), VIS, NIR (designated as UVN), and SWIR radiation. The UV spectrometer has a spectral range between 0.270 and 0.320 μm, the VIS spectrometer has a UV to blue-green spectral range between 0.310 and 0.500 μm, the NIR spectrometer has a far-red to NIR spectral range between 0.675 and 0.775 μm, and the SWIR spectrometer has a range of 2.305–2.385 μm.

**Fig. 11:**
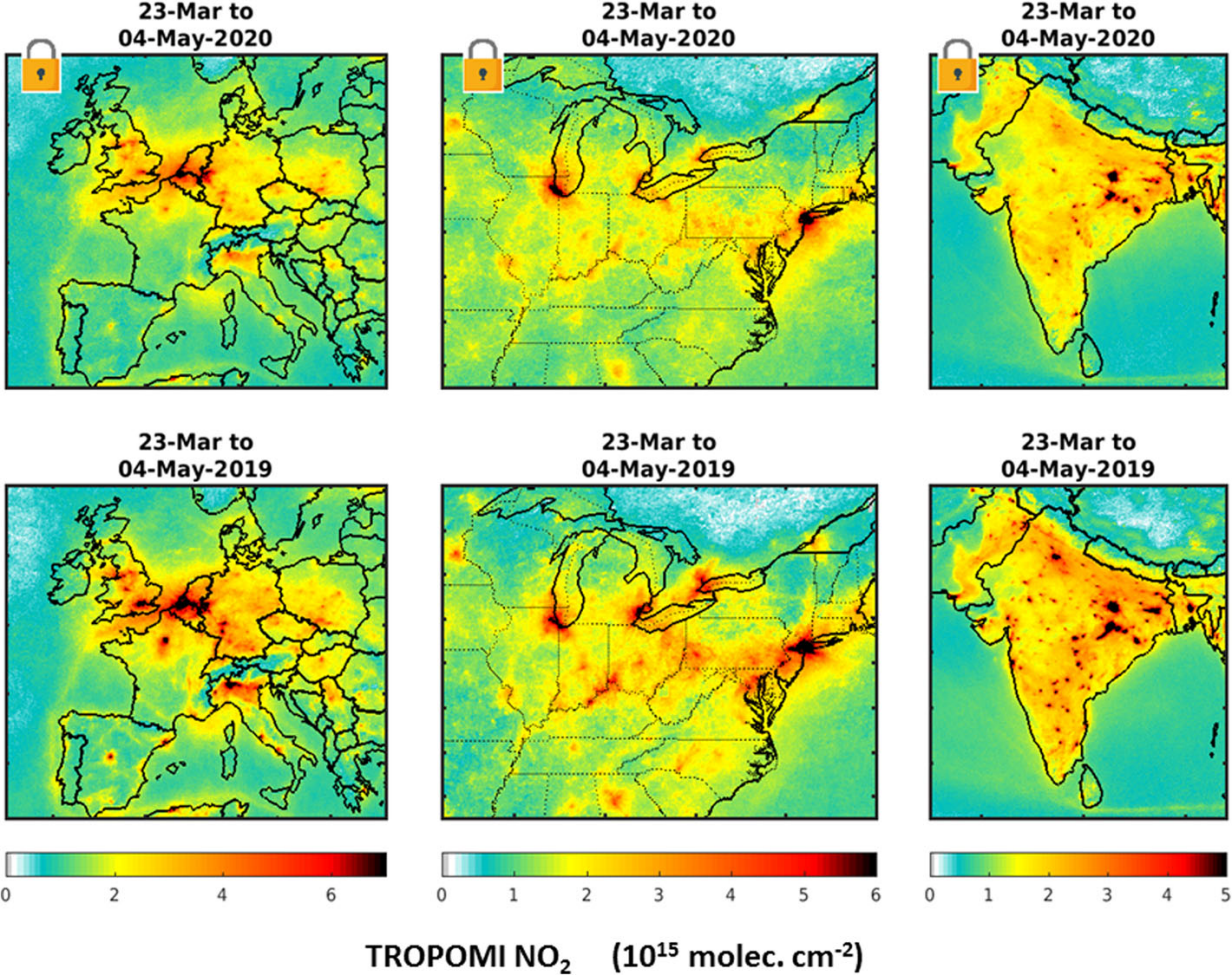
TROPOMI (S-5P) data showing changes observed in tropospheric NO_2_ over urban areas during the COVID-19 outbreak compared to the same period in the previous year. (From: the Royal Belgian Institute for Sapce Aeronomy (BIRA-IASB), in collaboration with the Royal Netherlands Meteorological Institute (KNMI) and the European Space Agency.https://www.aeronomie.be/en, then clicking on: "Satellites see a worldwide decrease in nitrogen dioxide pollution as a result of the COVID-19 crisis, China shows first signs of economic recovery''. Modified S-5P data, press release after Bauwens et al. 2020 was published on May 8, 2020. Accessed September 6, 2020

TROPOMI’s ascending overpass at 13:30 is in the same orbit and trails behind the US Suomi NPP platform by 3.5 min, offering possibilities to link atmospheric chemistry data more closely with plant and soil physiological processes derived from VIIRS’s land data acquired on Suomi NPP. The atmospheric chemistry data from S-5P and future LEO Sentinel S-5 and GEO Sentinel S-4 are expected to provide information about ecosystem conditions and environmental health that will greatly advance understanding of environmental responses to climate conditions. For example, monitoring during the coronavirus pandemic in early 2020 revealed dramatic reductions in NO_2_ concentrations in major global cities, consistent with restricted human activities (Fig. 11). This figure shows average reductions in NO_2_ emissions for the period 23 March to 04 May in 2009 and 2020 during restrictions (indicated by padlock on figure) for three regions in eastern Europe, northeastern USA and India. Chinese cities also showed similar patterns in NO_2_ reductions, especially the larger region around Beijing and secondarily around Shanghai (Bauwens et al. 2020).

#### Sentinel-4, Sentinel-5, and Sentinel-6 missions

The atmospheric chemistry missions for S-4 and S-5 will build on the S-5P atmospheric mission and the subsequent S-6 to measure sea surface heights. All three weather satellites will be launched before 2025 (Table 3). Sentinel-6 is being jointly developed by the ESA, NASA, the EU, EUMETSAT, and NOAA, and with support from the CNES. It is the first Sentinel to be named for a person, Dr. Michael Freilich, in honor of his lasting contributions to Earth Science and his work in advancing this mission. Dr. Freilich (1954–2020) was an oceanographer and NASA administrator of the Earth Science Division for 12 years. The S-6 mission will now be called “Sentinel-6 Michael Freilich.” It will carry a radar altimeter that observes annual changes in sea level height to within 1 cm measurement precision, building on a 26-year history of ocean surface topography available from the French-US Topex-Poseidon, the Jason missions, Envisat, and Sentinel-3. The S-6 mission provides continuity of service to the Jason satellites for which they are also called Jason-CS A and Jason-CS B.

Because the main missions of these three satellites (S-4, S-5, S-6), and S-7, identified in the next section, are related to atmospheric and oceanic processes, we do not provide additional details about them here as they are not of primary interest for ecological and environmental monitoring.

### Copernicus 2.0

The Copernicus Program is anticipating its second generation of Earth satellites, designated as Copernicus 2.0. ESA will evolve the Copernicus Sentinels into the Copernicus Services program that will focus on societal challenges such as urbanization, food security, rising sea levels, diminishing polar ice, natural disasters and of course climate change. Several high-priority candidate missions are being evaluated to address EU policy concerns and gaps in Copernicus user needs, and to expand the current capabilities of the Copernicus space component. The first satellite selected under Copernicus 2.0 is Copernicus Anthropogenic Carbon Dioxide Monitoring (CO_2_M) mission to be Sentinel-7, dedicated to monitoring anthropogenic CO_2_ emissions. The CO_2_M mission, to be flown in 2025, aims to identify specific sources of carbon emissions and will be a constellation of three satellites viewing the Earth’s surface across 250 km swaths. CO_2_M will be a significant improvement over current orbiters that view more limited regions with narrow swaths. This program complements NASA’s OCO-2 mission and the OCO-3 instrument on the International Space Station.

Although five other high-priority mission candidates are being evaluated for Sentinel-8, the most likely choice is the Copernicus Land Surface Temperature Monitoring (LSTM) mission, to be flown in 2025 (Table 3). If selected, LSTM will fly as a companion to the Sentinel-2 satellites, providing a high spatial-temporal resolution TIR imager for monitoring land surface temperature in order to quantify urban heat islands and to derive evapotranspiration products that are necessary to understand and respond to climate variability. In addition, the LSTM will provide critical information for managing water resources for agricultural production and coastal and inland waters, as well as predicting droughts, addressing land degradation, and tracking natural hazards such as fires and volcanoes. NASA’s plans for the thermal instrument on the SBG mission may be affected by the EU’s decision to fly LSTM.

Sentinel-9 and Sentinel-10 remain undecided but will likely be chosen from these candidates: CHIME (Copernicus Hyperspectral Imaging Mission) for which specifications are under development, CRISTAL (Copernicus Polar Ice and Snow Topography Altimeter), CIMR (Copernicus Imaging Microwave Radiometer), and ROSE-L (L-band SAR). At this time, their detailed designs have not been released. The CHIME mission is especially relevant to ecological studies and would provide ESA with a hyperspectral imager to acquire critical satellite data to address ecological and societal applications. This full-solar spectrum hyperspectral imaging mission would complement the multispectral Copernicus S-2 for land cover mapping with improvements for resolving health status of ecosystems including enhanced services for sustainable agriculture, biodiversity management, characterization of soil properties, mineral deposits for mining, and changes in the natural environment. Discussions are underway for coordinating this mission (and the LSTM) with NASA’s future SBG mission (described below).

### The ESA Earth Explorers

ESA’s Earth Explorer missions comprise a separate satellite series in addition to the Sentinels, to support cutting-edge science and technology testbeds that contribute to global understanding of our planet. Explorers are ESA’s Principal Investigator-driven missions (in contrast to the operational Sentinel missions). These missions address high-priority science areas that are not sufficiently mature to demand an operational mission; thus, these have limited flight commitments of 2–5 years. Designed for research purposes while demonstrating breakthrough observing technologies, Earth Explorer missions fall into three categories: “Core” missions addressing specific areas of great scientific interest (EE-1, EE-5, EE-6, EE-7); faster, lower cost “Opportunity” missions to address key scientific challenges identified by the Earth science community (EE-2, EE-3, EE-4, and EE-8); and “Fast-track” missions (EE-9). Of these, only four have relevance to environmental monitoring and processes of the land surface. Other Explorer missions address other key aspects of the whole Earth system, but are of less direct interest to ecological studies and are not included here but can be accessed at https://www.esa.int/Applications/Observing_the_Earth.

#### Earth Explorer-7 Biomass

The objective of the EE-7 Biomass mission (Table 3) is to quantify how much carbon and biomass are stored in the aboveground canopies of global forests and how biomass is changing due to climate and disturbances like wildfire, logging (harvest), and insect outbreaks. Biomass addresses a vital gap in understanding the global carbon cycle by mapping global forests for biomass and carbon storage and monitoring them over time with a P-band SAR (Table 3). P-band has good penetration of glaciers and sea ice, and dense vegetation canopies and soil, making it a good choice for estimating aboveground plant biomass; and because of its canopy penetration capability, it can provide information about underlying soil surface condition.

At a measurement scale of 200 m, the Biomass mission will provide the first systematic global measurements of forest biomass and ground surface topography. Biomass' radar bands are polarized to send and receive in either horizontal or vertical directions, or to send in one orientation and receive in the other. These polarization bands are analogous to adding spectral bands in a multispectral imager in that more bands provide more information (in addition to wavelength and spatial resolution) about surface structure. Vertical polarization responds most strongly to vertical elements (buildings, trees, steep terrain) in the landscape, horizontal to horizontal elements (land surface, roads, water bodies), and cross-polarization responds to intermediate orientations (multilayered forest, savanna, different crop types), thus contributing to understanding the 3-D structure of a landscape. Interferometry is measured when data from multiple overpasses are compared, allowing small changes in height or position to be detected, quantified and mapped, thus permitting detection of forest logging, selective logging or regrowth and big changes from an earthquake, tornado, or hurricane.

#### The Earth Explorer-8 FLuorescence EXplorer (FLEX) mission

**Fig. 12:**
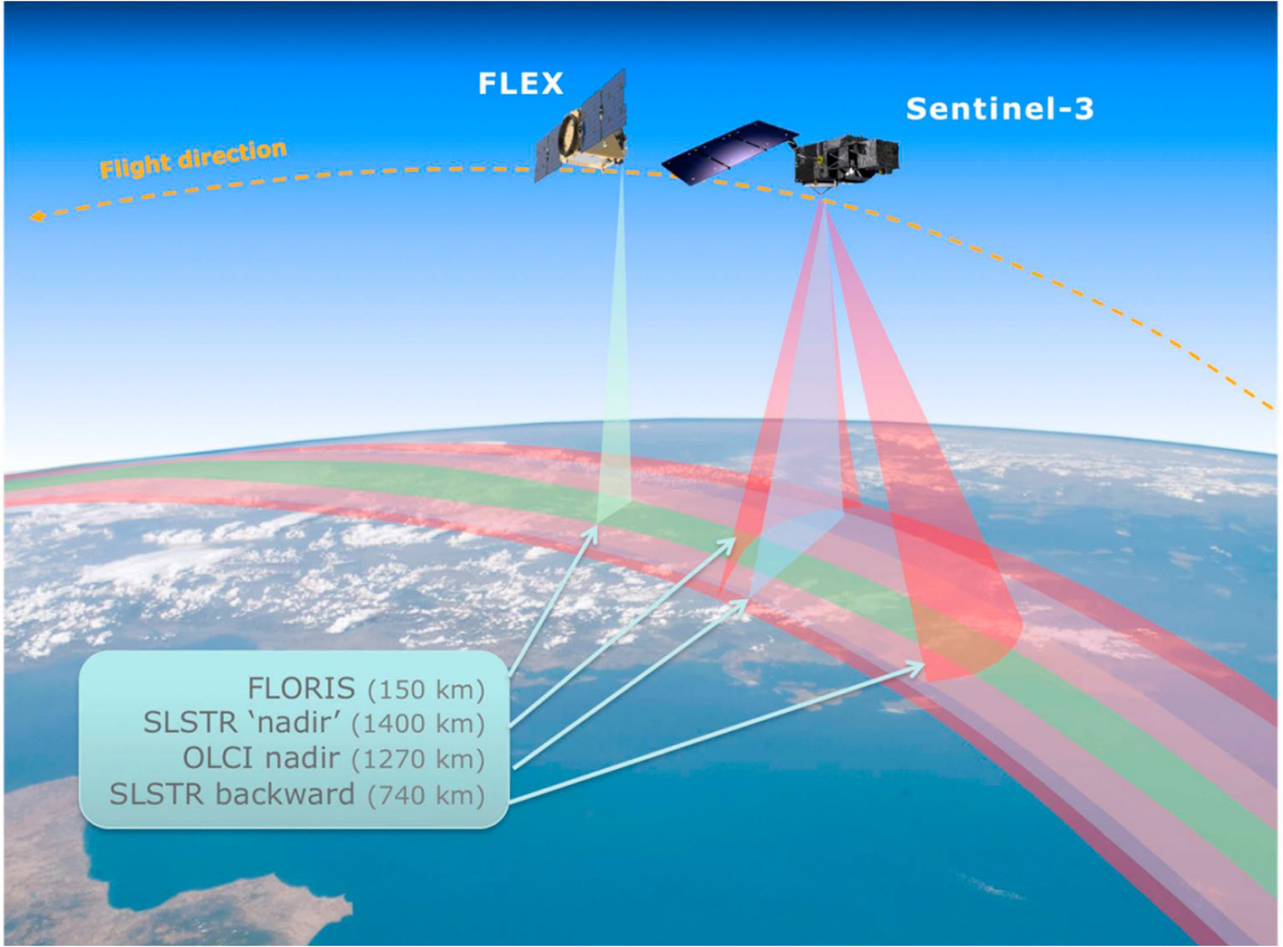
The FLEX Earth Explorer 8 mission consists of a fluorescence instrument, FLORIS, and Sentinel-3C. FLEX will fly ahead of S-3, overlapping swaths of OLCI and SLSTR instruments. (from https://directory.eoportal.org/web/eoportal/satellite-missions/f/flex. Accessed 19/04/2020)

The EE-8 FLuorescence EXplorer (FLEX) mission will carry the FLuORescence Imaging Spectrometer (FLORIS), which will be the first satellite mission to obtain, and seasonally monitor, high spatial resolution (300 m pixel) retrievals of solar-induced chlorophyll fluorescence (SIF) over land areas globally (Table 3, Fig. 12). FLEX is designed as a tandem mission with Sentinel-3C, which will provide essential supplementary optical and thermal surface measurements, and other necessary atmospheric information (Drusch et al., 2017; Mohammed et al. 2019).

**Fig. 13:**
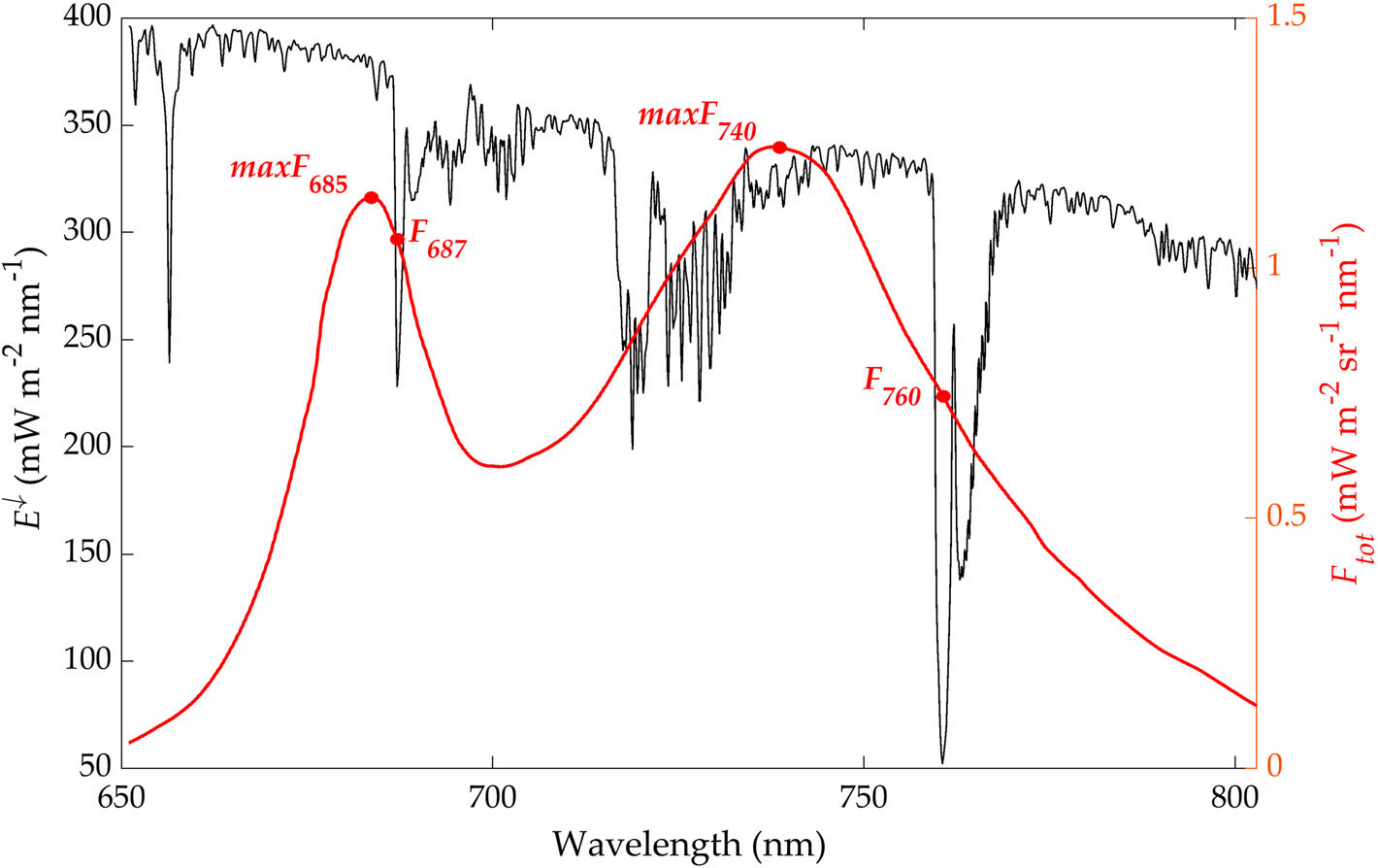
The black line shows irradiance at ground level and the red line shows a typical chlorophyll fluorescence spectrum, with fluorescence peaks at 685 nm and 740 nm (maxF685, maxF740). Solar-induced fluorescence (SIF) is estimated at the closest Fraunhofer lines to these maxima, located within the O_2_B lines centered at 687 nm and O_2_A lines centered at 760 nm (F687, F760). Figure with permission from Cendrero-Mateo et al. 2019

Solar energy absorbed by vegetation as photosynthetically active radiation (PAR) is utilized to drive the photosynthetic process. But plants protect photosystems by passively discarding excess absorbed energy as either thermal energy or as photons emitted by chlorophyll *a* molecules in the red and far-red wavelengths (i.e., SIF, 650–800 nm), producing fluorescence peaks at 685 nm and 740 nm (Fig. 13). As SIF values increase and decrease, patterns of stress are revealed. FLEX will assess the quality of fluorescence-derived photosynthesis data, as measured for SIF emissions that can be retrieved in the narrow wavelength bands associated with the two primary atmospheric oxygen absorption features, O_2_-B and O_2_-A, centered at 687 nm and 760 nm, respectively (Theisen 2000). FLEX promises to improve our understanding of how carbon transfers between cycles in the atmosphere, biosphere, and hydrosphere.

**Fig. 14:**
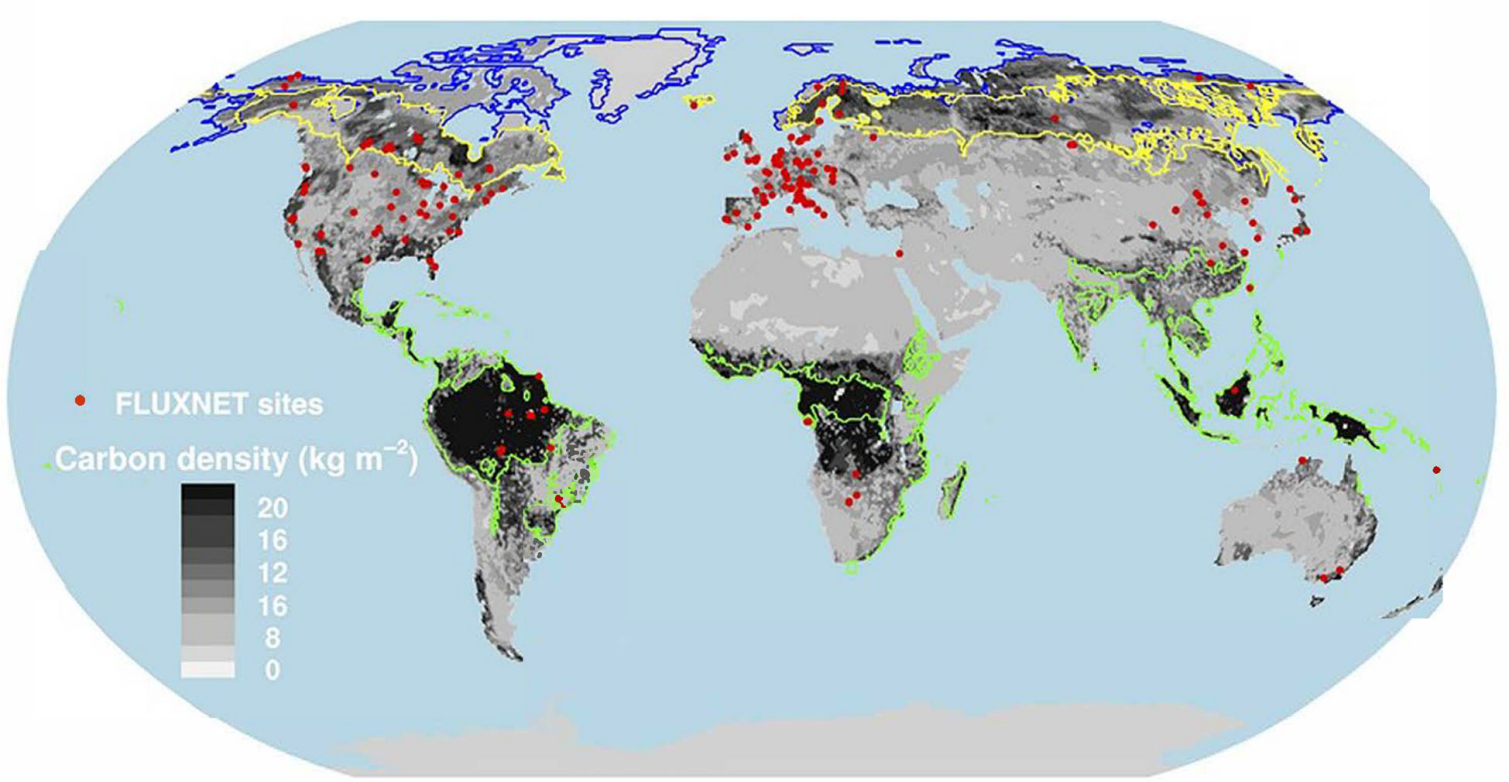
Climate tipping elements occur where biomass storage is high and at risk for large losses due to climate change (Lenton et al. 2008). Three climate tipping elements are shown in blue (frozen regions), green (tropical forests), and yellow (boreal forests) overlayed on a map of terrestrial vegetation and soil carbon storage. The Red dots indicate flux tower locations, which are largely located outside of the tipping element regions, illustrating the need for satellite measurements to fill gaps in ground sampling. Original figure from Schimel et al. 2014; data from Ruesch and Gibbs (2008) and FAO et al. (2009)

FLORIS is a dual-spectrometer imaging system on FLEX, consisting of narrow-band (0.3 nm) and wide-band (3–5 nm) spectrometers, measuring the spectral range between 500 and 780 nm to capture the full SIF emission range as well as reflectance for vegetation indices. Instruments from S-3C will provide atmospheric and thermal data, geolocation, and other ancillary data. Mission products will be derived from harmonized Top of Atmosphere (TOA) synergy data using FLORIS and OLCI, and SLSTR radiances from Sentinel-3 that have been cross-calibrated, geometrically co-registered, and orthorectified to a common 300 × 300 m^2^ grid. The need for the FLEX mission (Fig. 14) and its value in monitoring early warning changes in SIF that indicate losses (or gains) in carbon is highlighted by these “tipping point” regions.

### Other notable national satellite programs for Earth observations

We note that the space agencies of several countries have multispectral or hyperspectral optical imaging orbital instruments, including Japan, China, and India. But data from most of these programs are either not publicly available or are not cost-free to users. There are also a growing number of commercial civilian programs that are collecting high-resolution data (either spectral or spatial) primarily for application users who pay for data, such as SPOT, WorldView, and Planet, and many others are emerging. As described earlier, much of the older data (often after 5 years) is available on various public websites. We highlight several instruments sponsored by international programs of interest next, from which mapping data could be of considerable interest to environmental science studies and which follow the free and open data access policy.

#### Vegetation and Environment monitoring on a New Micro-Satellite (VENμS)

The Vegetation and Environment monitoring on a New Micro-Satellite (VENμS) program is a research demonstration mission for the GMES (Global Monitoring for Environment and Security) program, a joint initiative of ESA and the EC (European Commission) launched in 2017 (Table 4). Dedicated to monitoring vegetation, it sets the foundation for an operational GMES observatory, designed to monitor the environment and manage natural resources. VENμS provides frequent fly-by observations (2-day repeat) at high resolution (5 m) and at constant viewing angles at the same time of day for an instrument with 12 spectral bands. Itwas flown in a sun-synchronous descending orbit with a 10:30 local equatorial crossing time, acquiring 27 km × 27 km individual images at nadir in the initial sampling mission (VM1) at 720 km altitude for ~2.5 years, after which it was moved into a lower orbit (VM2) at an altitude of 410 km to support a technology mission in its final year. During its science sampling mission, the satellite consistently imaged 110 sites all over the world, which were chosen for specific experiments, primarily a vegetation and ecological focus by the French Space Agency, CNES. Time-composited images are produced that are cloud-and-aerosol free. VENμS data are free to registered researchers, with the primary application being for vegetation studies, including land cover change detection, biodiversity and environmental function, crop growth conditions, precision farming, etc.

#### Pléiades Constellation and Pléiades NEO

The Pléiades Constellation consists of two commercial satellites (Table 4), first flown in 2011 and 2012 that share orbit planes with the SPOT 6 and 7 satellites. Together, this gives them rapid repeat coverage. The instrument has five spectral bands in the VNIR wavelengths, including a panchromatic band with 57 cm pixels and four multispectral bands with 2.8 m pixels. With pointing, it can acquire data nearly everywhere within 2 days.

The Pléiades NEO (Table 4) will consist of four satellites, with the first pair flown in 2020 and the second pair in 2022. The satellites will be placed at 90° positions from each other and will continue in the orbital plane of SPOTs 6 and 7. It will also have seven bands in the VNIR, with a pan band at 30 cm and six multispectral bands at 1.2 m pixels. Because of its redundancy and pointing capability, it will be able to create very high spatial resolution mono, stereo, and tri-stereo acquisitions to make detailed DEMs of the surface materials.

#### Satellite Pour l’Observation de la Terre (SPOT)

The SPOT family of satellites (Table 4), developed by the French CNES and the SPOT Corporation, was the first to launch a commercial satellite in 1986. It had 3 bands (G, R, NIR) and data with 20 m pixels. Since then, SPOT has flown SPOTs 2, 3, 4, 4 Take 5, 5, and 5 Take 5. Starting in 2012 and 2014, the newest satellites SPOTs 6 and 7 were built by Airbus Corporation. All of these have had tandem flights to increase the frequency of data collections from 26 days to 13-day repeats. SPOTs 6 and 7 fly in tandem with Pléiades 1A and 1B but also with the TerraSAR-X and TanDEM-X (from the German Space Agency, DLR), high-resolution radar satellites that provide global DEMS at high spatial resolution. Note that the older data are available in several locations. As described in the “Commercial satellites” section.

#### PRecursore IperSpecttrale della Missione Applicativa (PRISMA)

**Fig. 15:**
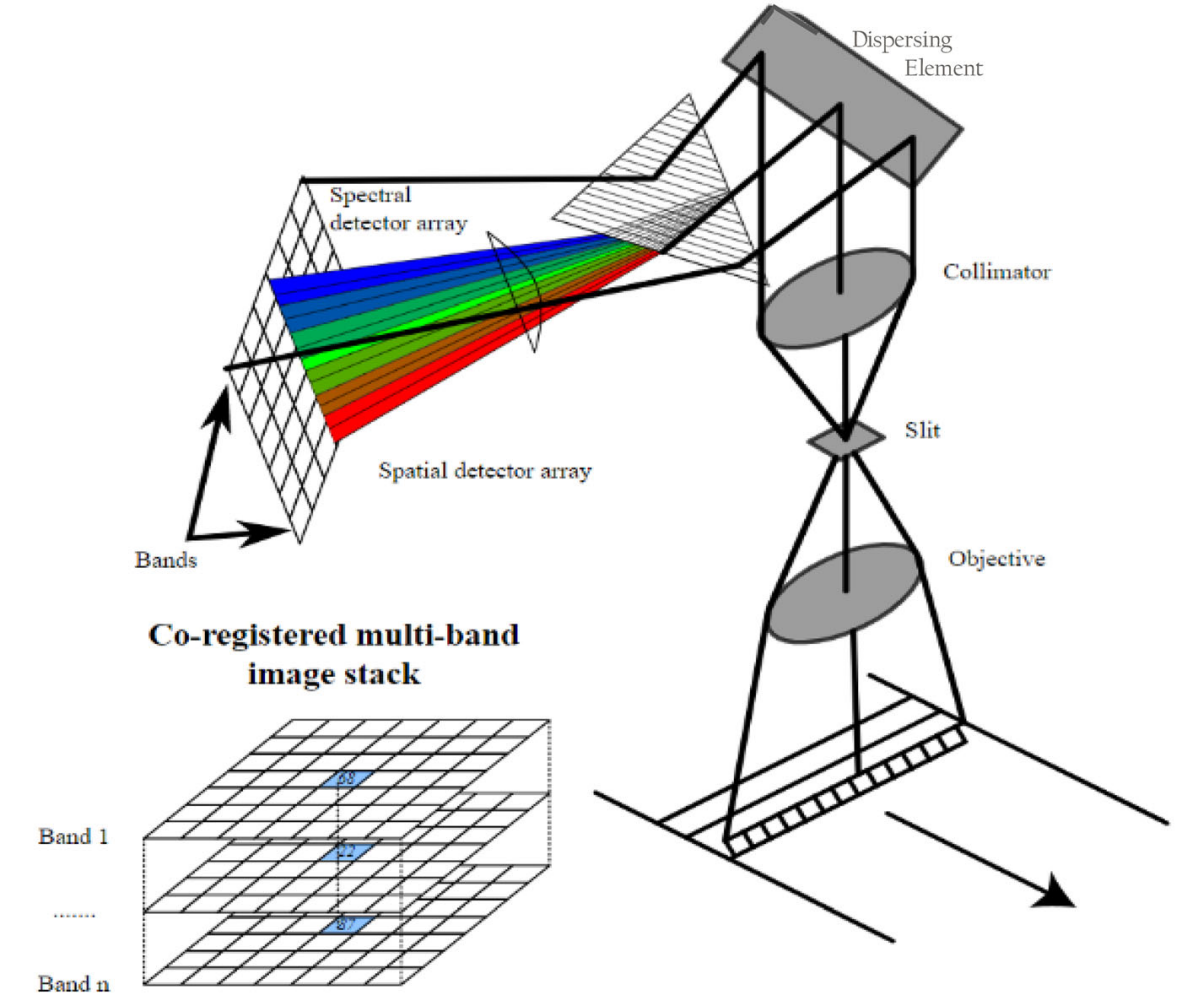
Imaging spectrometers measure a full spectrum for each pixel. Light from each pixel area enters the objective and is directed through the entrance slit and onto a collimator before it intersects the dispersing element, such as a prism (as for PRISMA) or a diffraction grating, which disperses the light into different wavelength intervals onto the spectral detector array (as for EnMAP), which dispurses light onto the spectral detector array (one detector per spectral band). Prior to the dispersing element, both PRISMA and EnMAP split the light into two pathways, with different spectral detectors for the VNIR spectrum and the other for the SWIR spectrum. Figure modified from a license under the Creative Commons Attribution 3.0 Unported License. Author: Arbeck

The PRISMA satellite (Precursor Imaging Spectrometer for Mission Applications) from the Italian Space Agency was launched in 2019 (Table 4). This sampling mission, based on programmed user and project requests, focuses on collecting spectroscopy (i.e., hyperspectral) images over Europe and northern Africa, with a priority to observe Italy, to detect and monitor ecosystem health and pollution of natural resources, agriculture, soils, inland waters, and coastal zone of the Mediterranean Sea. PRISMA is Italy’s new technology development in VSWIR imaging from space, building upon spectroscopy technologies pioneered by the EO-1’s Hyperion and ALI and ESA’s CHRIS PROBA, but using a prism optical design (Fig. 15). PRISMA is a sun-synchronous satellite in LEO with a morning descending orbit, 30 m pixels, and a swath of 30 km. It has a panchromatic camera with 5 m pixels and the hyperspectral imager has about 250 bands from 400 to 2500 nm, with an average bandwidth of 12.5 nm (Table 4). The data are now available to the scientific community at the Italian Space Agency (ASI) after a delay allowing Italians to have first access; registration is required.

#### Environmental Monitoring and Analysis Program (EnMAP)

**Fig. 16:**
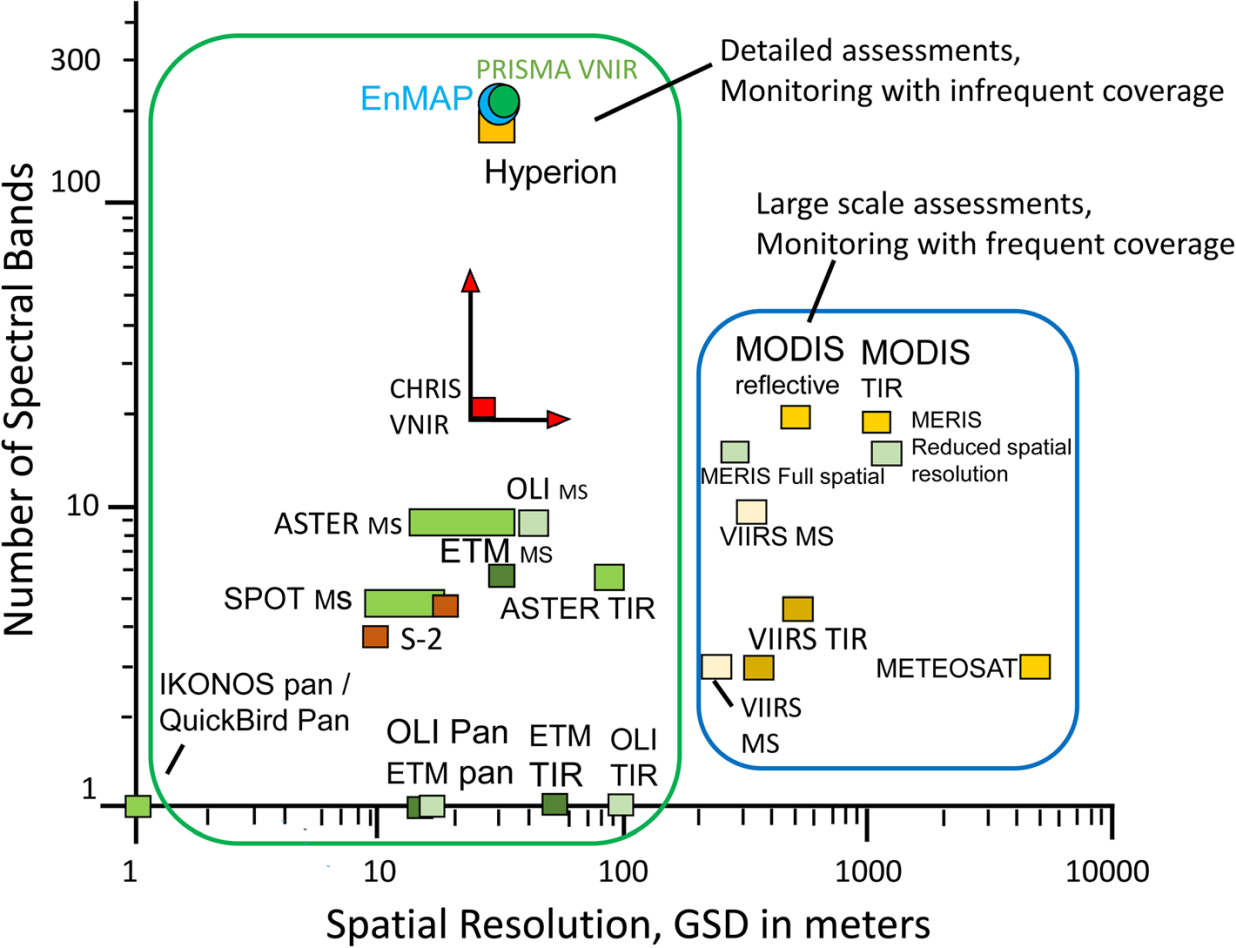
Comparison of EnMAP spectral bands and pixel (ground spatial) resolution with other optical imagers. Image credit: OHB/Kayser-Threde, GFZ, from: https://earth.esa.int/web/ eoportal/satellite-missions/e/enmap

EnMAP is planned for launch by the German Space Agency (DLR) in late 2020 or early 2021. It is also a new generation of VSWIR hyperspectral imaging system to provide high spectral resolution sampling at 5 to 20 nm per band (Fig. 16) across the 400–2500 nm range with a pixel resolution of 30 m (Table 4). EnMAP is also a sampling mission that will acquire up to 5000 km along-track data per orbit from user or mission requests (similar to NASA’s retired EO-1 Hyperion). EnMAP’s spectroscopy imagery will provide detailed monitoring and characterization across the globe of rock/soil targets, vegetation, and water quality information from inland and coastal waters. This information is key to improving understanding of the impacts of climate change, extreme weather, invasive species, pollution, and other stressors on global ecosystems. EnMAP has 96 bands in the VNIR (mean bandwidth 6.5 nm) and 136 bands in the SWIR (mean bandwidth 20 nm) providing sufficient density of spectral information to retrieve many key biochemical signals such as plant biochemical constituents and canopy structure information. EnMAP data are under a free and open policy (as DOI-referenced data), but different classes of users can request new target sites to be acquired, in an order of priority.

#### The WildFireSat Mission

WildFireSat is a satellite initiative with a planned launch late in 2024 by a consortium of the Canadian Space Agency (CSA; Table 4), the Canadian Forest Service (CFS), as part of Natural Resources Canada (NRCan), and Environment and Climate Change Canada (ECCC), tasked with monitoring all active wildfires in Canada on a daily basis. WildFireSat’s scientific objective supports research on the behavior of wildfires and the emission of carbon, aerosols, and other particles produced by wildfires. WildFireSat will obtain key daily observations of wildfire activity and Fire Radiative Power (FRP) during the peak seasonal wildfire period, and will also draw upon other existing, contemporary satellite data to significantly broaden understanding of how wildfires behave and how behavior is changing due to climate influences. WildFireSat measurement goals include forecasting wildfire smoke trajectories, fire spread and fire boundaries, and total column water vapor. The WildFireSat mission plan consists of one or more satellites to acquire two measurements of the surface condition in short succession near peak wildfire burn in late afternoon. It will be equipped with infrared sensors to measure fire intensity (i.e., RFP), in addition to wildfire-generated carbon emissions and smoke pollution. WildFireSat will use an innovative type of IR sensor, based on microbolometer technology that does not need to be cooled. This technology approach allows reductions in the satellite’s weight, size, and operating power, and therefore, the cost, which could revolutionize longwave measurements from space.

### The International Space Station

**Fig. 17:**
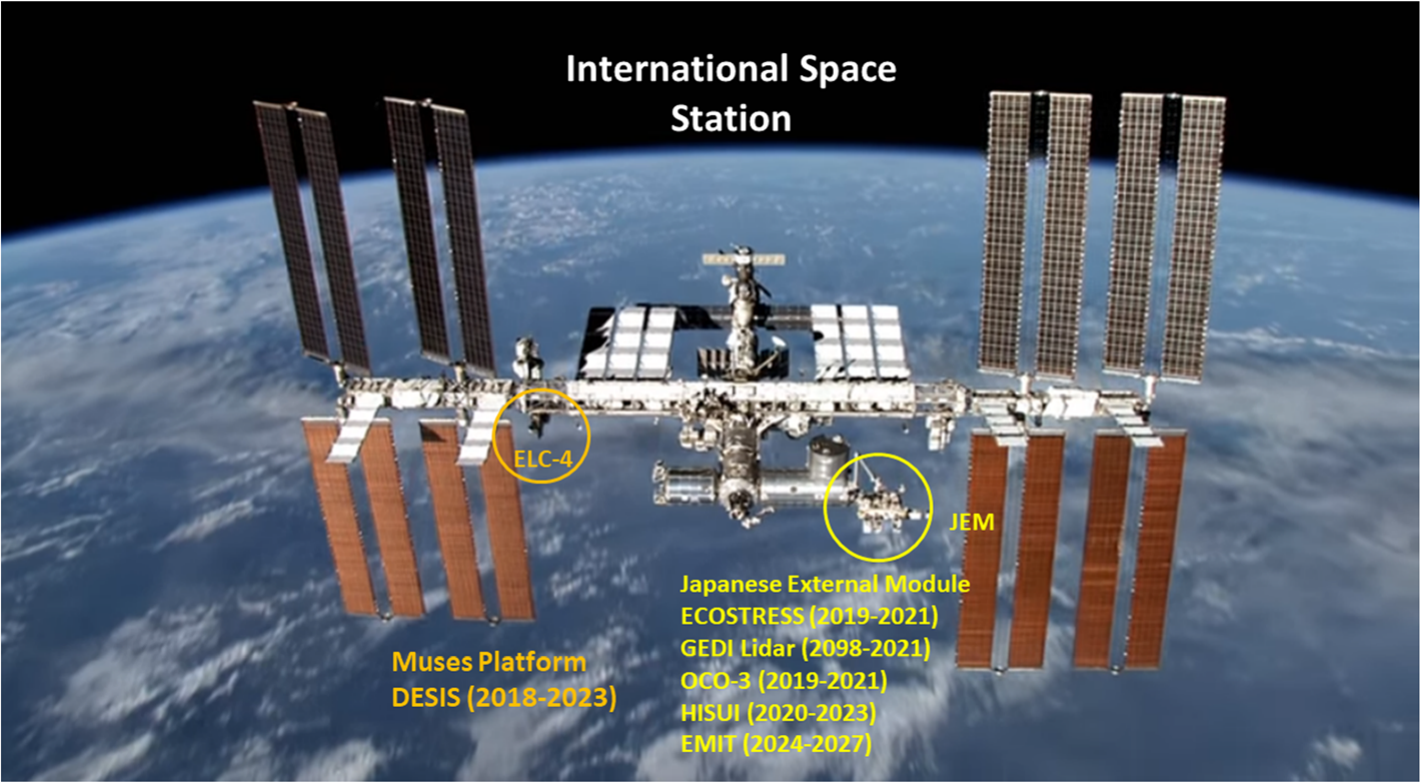
The ISS observed on 21 February 2018 showing the positions on the Muses Platform where DESIS resides, and the Japanese External Module (JEM) where five new Earth observing instruments are located. Co-location allows synthesis of data from these sensors, whch has potential to significantly advance scientific understanding of earth system processes. Image courtesy of Michael Freilich, NASA Headquarters

The International Space Station (ISS) (Fig. 17) provides a unique opportunity to host Earth observing instruments at half the altitude of typical LEO satellites (400 km vs. 700–800 km), which also can provide higher spatial resolution data. It costs less to send instruments to the ISS than into LEO (https://directory.eoportal.org/web/eoportal/satellite-missions/content/-/article/iss-muses), but more importantly, instruments can be replaced if they fail or repaired on-site. The disadvantage is that the ISS is not in a sun-synchronous polar orbit but rather in an oblique 50° low-inclination orbit, meaning its orbits range from about 51° North to 51° South. Consequently, subsequent overpasses are shifted in time, although in a predictable fashion. It requires more detailed calibrations to compare imagery across different sun and view angles, and atmospheric conditions. Another non-ideal property of the ISS is the strong gravitational drag at this lower altitude, thus requiring powerful thrusters to periodically increase the altitude, so that the ISS regularly cycles between 300 and 400+ km above the surface. Despite these two technical problems, the system’s characteristics are known and are monitored at all times.

The ISS orbit opens up, for the first time, an opportunity to view locations at different times of the day and night, and potentially at higher spatial resolution than previously possible from global-scale observations. It also has more dwell time over temperate landscapes, which can increase the signal to noise ratio (SNR) of measurements. The orbits do not explicitly provide diurnal measurements within any given week because it takes a few weeks to accumulate data covering a 24-h period. True diurnal measurements can be obtained from the new third-generation geostationary weather satellites (NOAA, JAXA, ESA), but at 500 m to 1 km pixel sizes in solar bands and 2 km for thermal imagery.

**Fig. 18:**
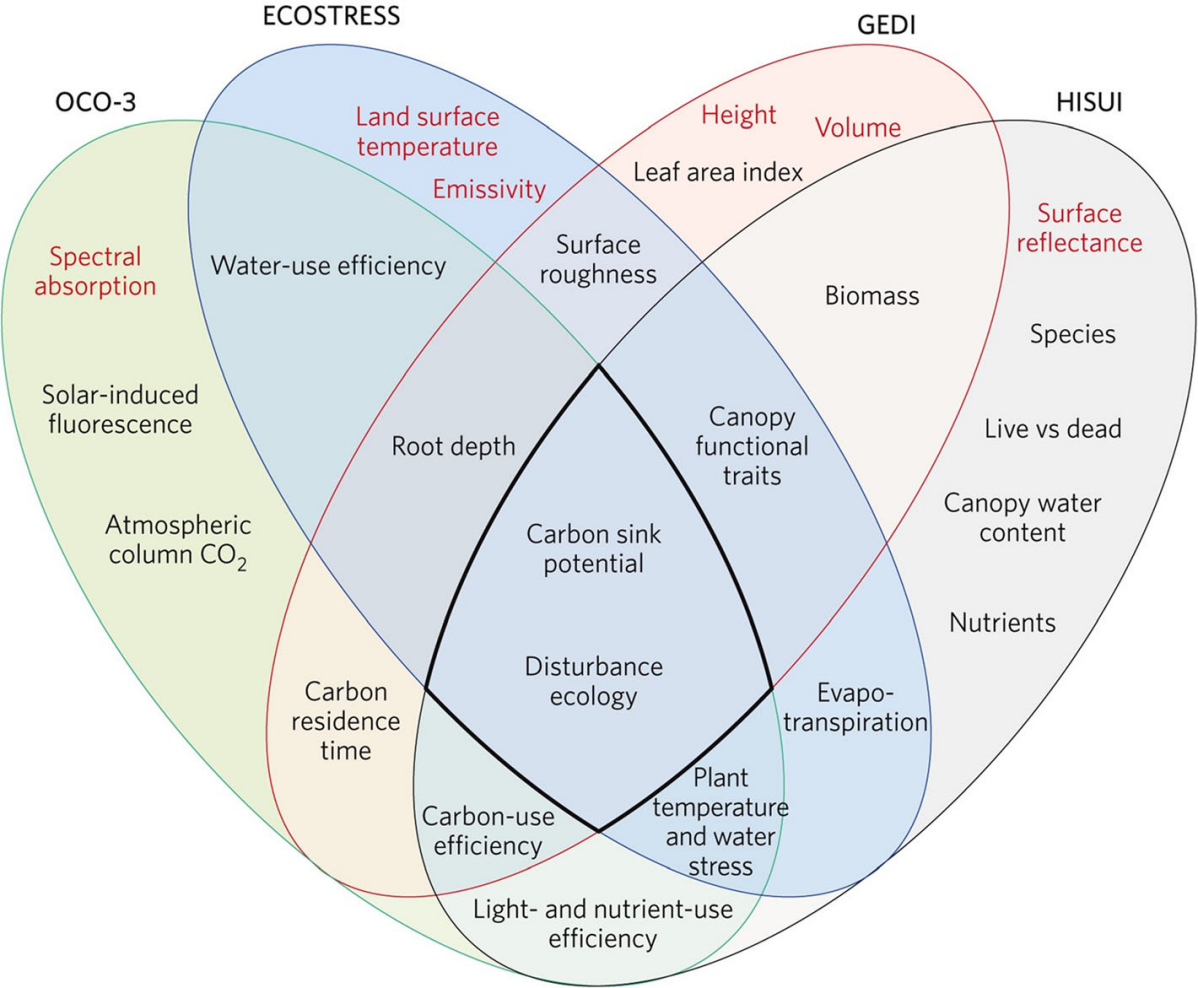
The capabilities of four hosted ISS instruments for Earth observations during 2019–2023 are shown. The measurement types of each are listed within ellipses: OCO-3 (green), ECOSTRESS (blue), GEDI (pink), and HISUI (grey). The overlaps are where multiple instruments will provide measurements of related variables. Additionally, the central region outlined in black is where all four instruments contribute measurements related to change detection and disturbance and the potential for supporting carbon sink quantification. Figure reproduced with permission from Stavros et al. (2017)

There is a unique opportunity now for ecosystem research on the synergies of several unique Earth observing instruments co-hosted on the ISS (described below) that measure different terrestrial and aquatic properties (Stavros et al. 2017). Data from these sensors will be available under free and open data policies. By 2021, there will be (or have been) six Principal Investigator-led Earth observing research missions on the ISS that promise important new types of data products individually. Possibly even more important, this contemporaneous measurement capability provides an unprecedented opportunity to combine these new data types in creative ways that advance much deeper understanding of Earth processes. The promise of the synergies possible for Earth observations is illustrated for four of the five ISS instruments (DESIS, ECOSTRESS, GEDI, OCO-3, and HISUI) described below (Fig. 18).

#### Earth Sensing Imaging Spectrometer (DESIS)

**Fig. 19:**
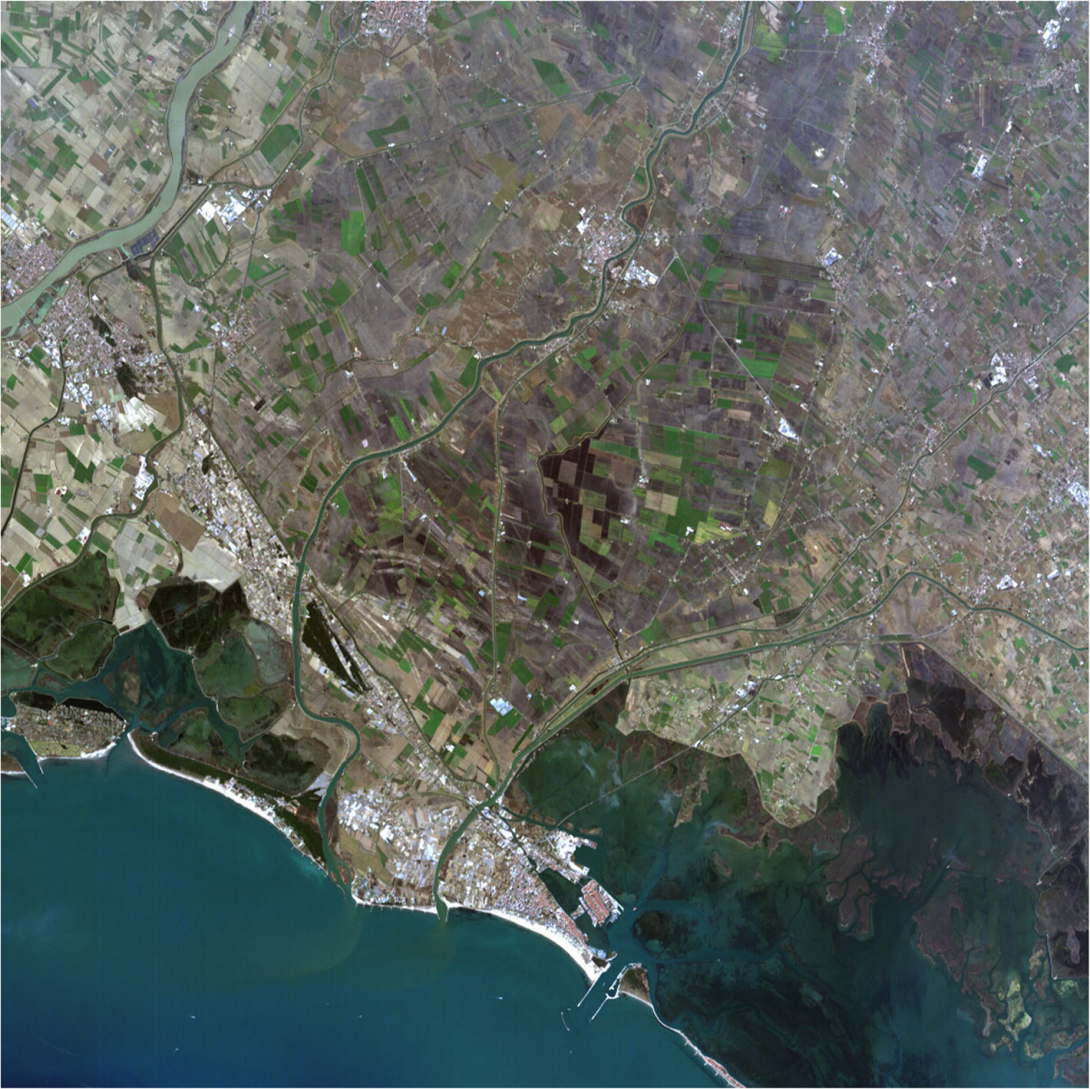
This is a true color image of the coastal region near the small city of Sottomarina, Italy (white pixels), located on a peninsula protruding into the Lagoon of Venice. On land, the agricultural fields are easily seen, while many features in the lagoon and coastal waters are evident. Note that the Brenta River cuts through farmland, sending a plume into the lagoon. This image was acquired by DESIS on 8 February 2019. Image from DLR Earth Observation Center (https://www.dlr.de/eoc/en/desktopdefault.as)px/tabid-13614/)

DESIS was installed by NASA on the ISS in 2018. It is a VNIR spectrometer (Table 4) developed collaboratively by Teledyne Brown Engineering (Huntsville, AL, USA) and the German Aerospace Center (Deutches Zentrum für Luft-und Raumfahrt; DLR, Cologne, Germany). DESIS was originally developed to support the DLR’s EnMAP satellite and to validate instrument technologies for that upcoming 2021 mission. DESIS, managed by Teledyne Brown Engineering, is integrated onto the MUSES (Multi-User System for Earth Sensing) platform on the ISS, shown in orange (Fig. 19), intended for hosting Earth-viewing commercial instruments (Table 4). Access to DESIS data at no cost is restricted to scientific and educational applications.

From this platform, DESIS can image about 90% of the area covered by the ISS’ orbit. This VNIR imaging spectrometer measures the spectral range from 402 to 1000 nm, with a native spectral sampling resolution of 2.55 nm, for which there are four band binning options of the 235 overlapping channels. The first option is no binning of the 235 channels (SNR at 195); the second option bins two adjacent channels to give ~ 5 nm spectral resolution; the third option of binning three adjacent channels gives a spectral resolution of ~ 7.5 nm; and the standard product is the fourth option that bins four channels to yield a spectral resolution of 10 nm (SNR at 386). The VNIR coverage provides information on water quality in lakes and rivers and along the coastal margins, as seen in a DESIS image (Fig. 19). This capability was originally provided by the first instrument hosted on the ISS—HICO (the Hyperspectral Imager for the Coastal Ocean). HICO was a demonstration instrument supported by the US Office of Naval Research (ONR) and collected data between 2009 and 2014, providing new insights into coastal environments around the world, information to be supplemented and updated with DESIS.

These high spectral resolution data are ideal for monitoring the spectra of different photosynthetic pigments (Schalles and Yacobi 2000), water constituents and pollution (Leifer et al. 2012), and other biochemicals (Krutz et al. 2019). For example, with 30 m pixels and narrow spectral bands, DESIS has the potential to identify different algal phyla based on their pigments or to detect different types of harmful algae (Craig et al. 2006) and cyanobacteria blooms (Kutser 2004; Kudela et al. 2015). DESIS also provides land cover mapping, especially in the tropics for forestry applications, where crossing at different times of day may increase chances of obtaining no or low cloud cover. The VNIR coverage provides information on fractional soil cover, soil texture, iron oxides, and other products, and on pigments and water in canopy foliage and LAI for the land areas it views.

#### ECOsystem Spaceborne Thermal Radiometer Experiment on Space Station (ECOSTRESS)

ECOSTRESS was selected for the ISS under the second NASA Earth Venture Instrument (EV-2) Pathfinder Program (Table 4). The design is based on the PHyTIR airborne instrument (Silvestri et al. 2020). ECOSTRESS is mounted on the Japanese Experiment Module (JEM) external facility of the ISS (Fig. 18). The ECOSTRESS, managed by the NASA/Jet Propulsion Lab (JPL, Pasadena, California, USA), was launched to the ISS in 2018 and has 5 TIR bands (8.29, 8.78, 9.20, 10.49, and 12.09 μm; Table 4). It has a repeat cycle (at different local times) of almost 3 days and spatial resolution of about 38 m × 69 m, which is significantly higher spatial resolution than other satellite TIR imagers. Gillespie et al. (1998) showed that kinetic temperature can be consistently separated from emissivity in thermal radiance data with multiple TIR bands, and the TIR bands on ECOSTRESS can perform this correction. Because the ISS orbit produces overpasses at different times of the day and night (spread over several days or weeks) as it completes a full cycle, it nevertheless provides an opportunity to evaluate generalized diurnal patterns of evapotranspiration and water stress and how these interact with the carbon cycle.

**Fig. 20:**
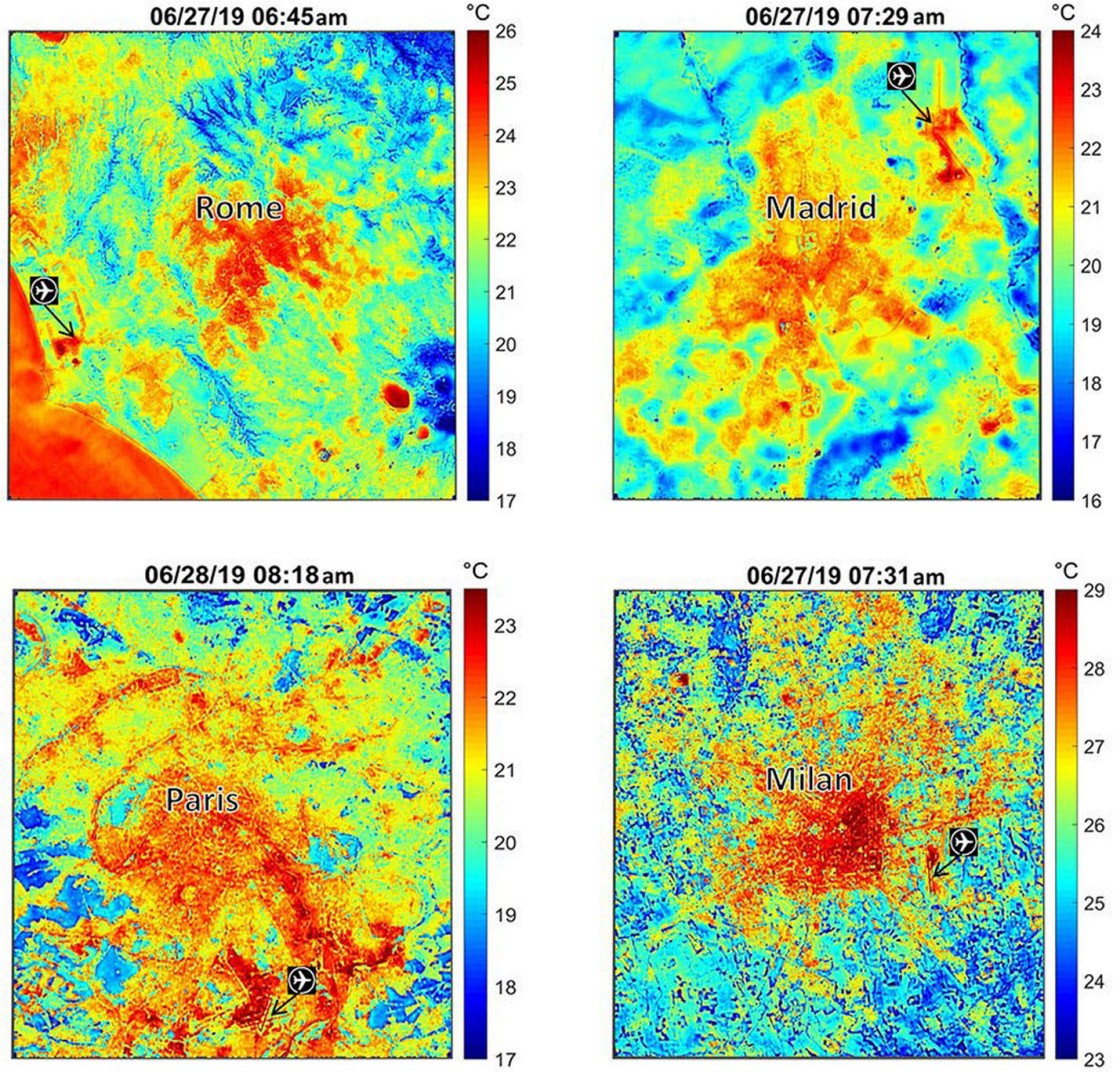
Early morning temperatures (between 06:45 and 08:18) over four European city centers (top row: Rome and Madrid); bottom row: Paris and Milan), measured by ECOSTRESS on 27 and 28 June during the 2019 heat wave. Urban temperatures were 6–9 °C hotter than temperatures in the surrounding areas (blue/aqua/green shades). Images were sharpened to delineate airports. Figure from NASA/JPL-Caltech. https://www.jpl.nasa.gov/news/news.php?feature=7445

Other types of thermal applications include monitoring of urban heat stress, volcanic eruptions, and wildfires (Silvestri et al. 2020). It is expected that the ECOSTRESS time series data will provide information on ecosystem resiliency and how different ecosystems respond to water deficits. TIR imagery will aid crop monitoring by documenting severe plant stress responses that potentially affect crop yields, enabling early warnings for irrigation requirements. The urban heat island effects of high morning temperatures in four European cities are distinct from temperatures in the surrounding countryside, during the 2019 heatwave, shown in Fig. 20.

#### Global Ecosystems Dynamics Investigation LiDAR (GEDI)

GEDI is a new NASA spaceborne laser instrument that provides a unique 3-D view of Earth’s aboveground structure and is contributing to a better understanding about the role of canopy structure in the carbon cycle (Table 4). It, like ECOSTRESS, was selected in 2014 for NASA’s EVI (Earth Venture Instrument proposals) and is managed jointly by the University of Maryland (College Park, MD, USA) and NASA/Goddard Space Flight Center (Greenbelt, MD, USA). GEDI will characterize the impacts of climate and land use changes on ecosystem structure and dynamics, primarily for forests. Data from GEDI data significantly improve our ability to quantify carbon stocks at high spatial resolution to better understand how the land surface interacts with atmospheric CO_2_, and whether the terrestrial carbon sink will continue or weaken in coming decades. The ISS orbit results in capturing most of the world’s tropical and temperate forests and data from GEDI are also expected to contribute to improved understanding of biodiversity in forests and habitat quality.

**Fig. 21:**
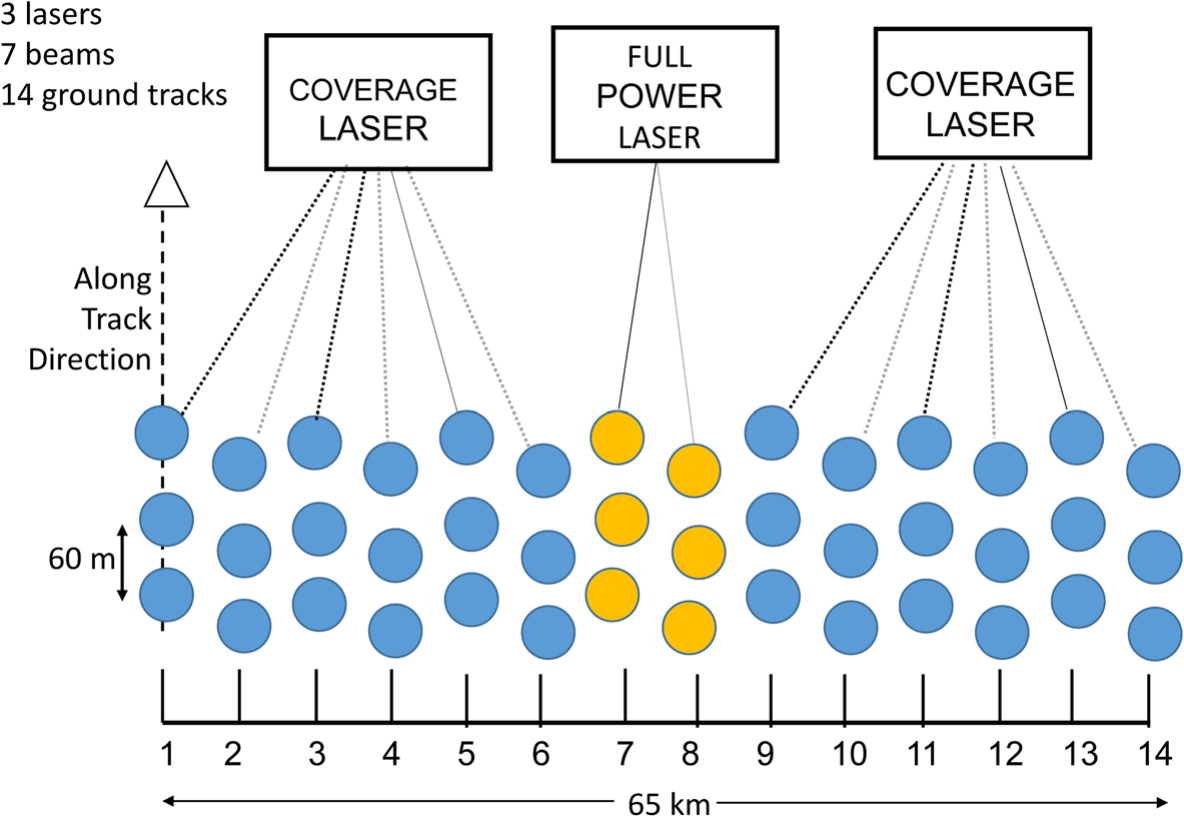
This diagram shows the pulse density and sampling patterns of the GEDI lasers. Each laser pulse produces a full waveform (based on all returns from the pulse) which is used to capture the full 3-D vertical structure from the ground to the tallest point. Redrawn from NASA, https://directory.eoportal.org/web/eoportal/ satellite-missions/i/iss-gedi

GEDI has a complicated sampling strategy (Fig. 21) that collects 14 ground tracks (Table 4). Each beam is ~ 22 m in diameter and these are collected 60 m apart along track and 600 m apart across track (Fig. 21), making the swath 65 km wide. As data are collected from multiple orbits over time, the space between ground tracks is filled in (Patterson and Healey 2015). Additional opportunities for filling the data gaps are being explored, with one option to merge GEDI data with the DLR’s TanDEM-X data, a system of two radar satellites obtaining high spatial resolution (12 m) spatial datasets with 2 m relative vertical accuracy. The first TanDEM-X radar platform was the TerraSAR-X, operational in 2008, followed by TanDEM-X in 2010. These are X-band SARs (wavelength 31 mm, frequency 9.6 GHz), flying in tight formation a few hundred meters apart and recording synchronous data. The DLR has released a global 90 m spatial resolution DEM dataset for scientific use through its Science Service System. Higher-resolution DEMs are available through their joint commercial venture with EADS Astrium (now Astrium SAS). The SAR images have a native resolution of 1 m^2^ with high-quality radiometric accuracy.

**Fig. 22:**
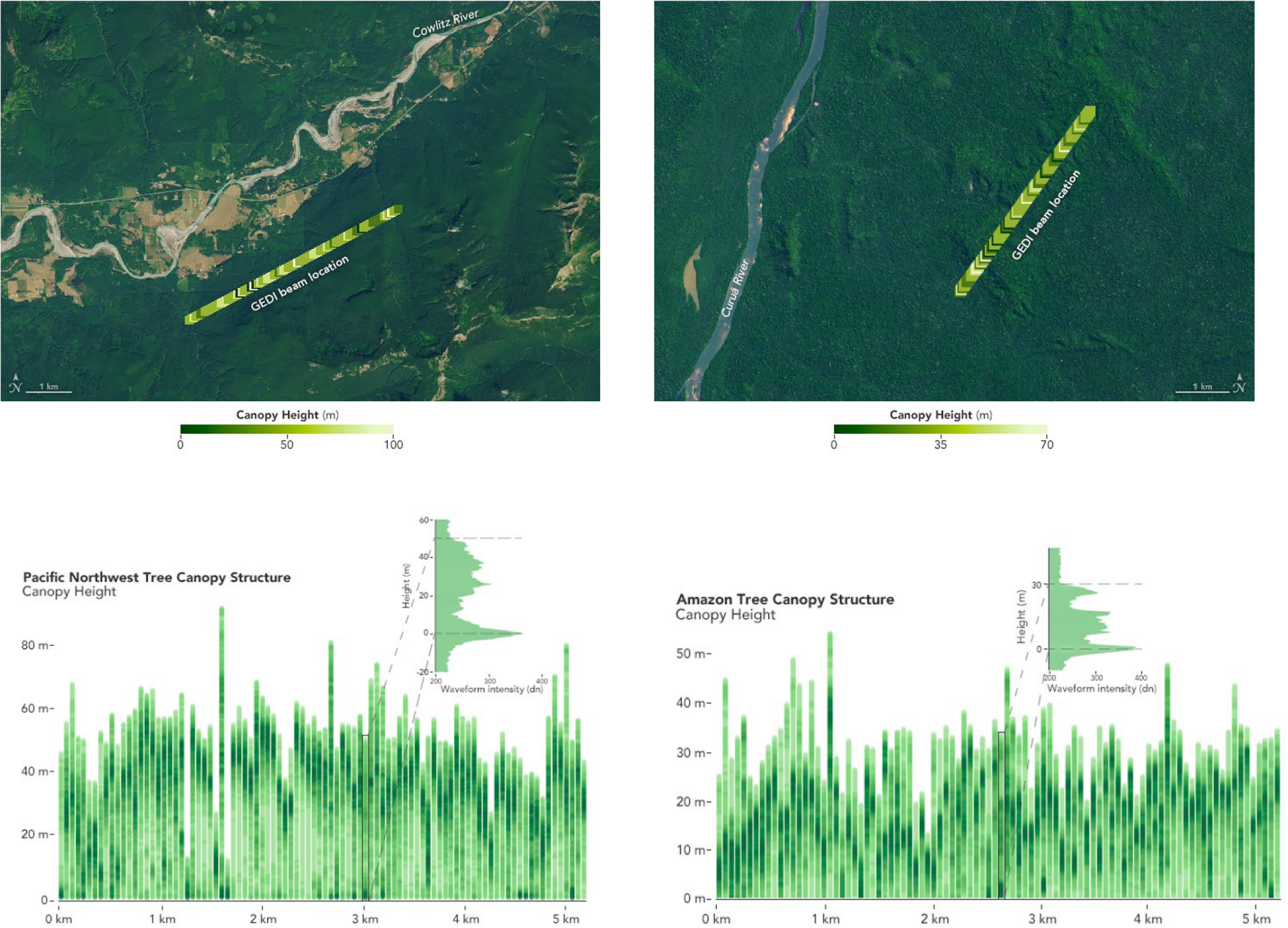
The upper panels show a strip of early GEDI data superimposed over true color Landsat-8 images, from July 29, 2019, for the Cowlitz River on the left and August 9, 2019, for the Curuá River on the right. The left panels are located over conifer forests in the Coast Range of southwestern Washington, USA, along the Cowlitz River, a tributary draining into the Columbia River, and (right) over the rainforest of Brazil, along the Curuá River, a small tributary of the eastern Amazon in the State of Para. (NASA.gov Credits: NASA Earth Observatory / Lauren Dauphin).

GEDI data provide both vertical and horizontal structure information from full waveform data of all LiDAR returns from the ground surface as well as those from the highest branches in the canopy. The spatial distribution of tree heights and vertical spacing are precisely measured and averaged for the pixel (Fig. 22). Areas of dark green in Fig. 22 indicate where the return density in the canopy is highest. The two expanded vertical distribution sections, connected by dashed lines to the position at 3 km along the horizontal distance, show the distribution of returns by height for that specific area and illustrate the full waveform information. In both forests, most returns are from the ground but the vertical distribution of foliage and small stems are quite different for the conifer site which has more growth in the upper canopy versus more growth in the lower canopy for the rainforest trees. Trees in the conifer forest are much taller (~ 50 m) with maximum heights more than 80 m, but in the rainforest, the average heights are closer to 30 m, with maximum height no greater than 50 m. These true color images (Fig. 22) show a transect (on the left) from southwestern Washington, USA, in the Coastal Uplands ecoregion of the Coast Range (as defined by the US Environmental Protection Agency). This ecoregion is composed of dense mature and regrowth conifer species known to be among the tallest trees in the world: Coast Douglas fir (*Pseudotsuga menziesii*; 60–75 m), western hemlock (*Tsuga heterophylla*; 50–75 m), western red cedar (*Thuja plicata*; 65–70 m), and Sitka spruce (*Picea sitchensis*; more than 90 m) (http://ecologicalregions.info/data/reg10/ORWAFront90.pdf). The panel on the right covers a transect along a tropical moist evergreen broadleaf rainforest in Brazil near the Curuá River, a small tributary of the Amazon, located within the ecoregion defined by the Tapajós and Xingu Rivers (https://globalforestatlas.yale.edu/amazon/ecoregions).

#### NASA Orbiting Carbon Observatory (OCO-3)

OCO-3, managed by a NASA/JPL team, was launched to the ISS in 2019 (Table 4). OCO-3’s primary mission is to collect space-based measurements to quantify variations in the atmosphere’s column-averaged CO_2_ dry air mole fraction, XCO_2_, with the precision, resolution, and swath coverage needed to reduce uncertainty in surface CO_2_ sources and sinks at scales of about 1000 km (Table 4). In addition, OCO-3 measures solar-induced fluorescence (SIF) from ecosystems. OCO-3 includes a 100 m resolution optical camera to provide context for understanding the SIF and CO_2_ data. OCO-3’s primary instrument is similar to the polar-orbiting OCO-2 mission, because it was built from spare parts of that instrument. Because the ISS orbit at 410 km is significantly below the 705 km altitude of OCO-2, OCO-3 has a much higher spatial resolution. Its data also differ from OCO-2, which acquires observations at a fixed time in early afternoon; OCO-3 benefits from the non-sun-synchronous orbit of the ISS to examine diurnal trends. It has three bore-sighted diffraction grating spectrometers that measure reflected sunlight in the NIR and SWIR wavelengths in three spectral regions centered at around 0.765 μm (O_2_ A-band), at 1.61 μm (a weak CO_2_ band), and at 2.06 μm (a strong CO_2_ band). For SIF retrievals, extremely narrow-band data are measured in Fraunhofer lines located close to the O_2_-A feature. These are narrow (< 0.1–0.5 nm) drop-out lines in the solar spectrum due to absorption at the sun’s surface, and therefore, no sunlight in these wavelengths reaches the Earth. Upwelling TOA radiation measured in these narrow lines is assumed to come from SIF emitted by plants at the land surface.

**Fig. 23:**
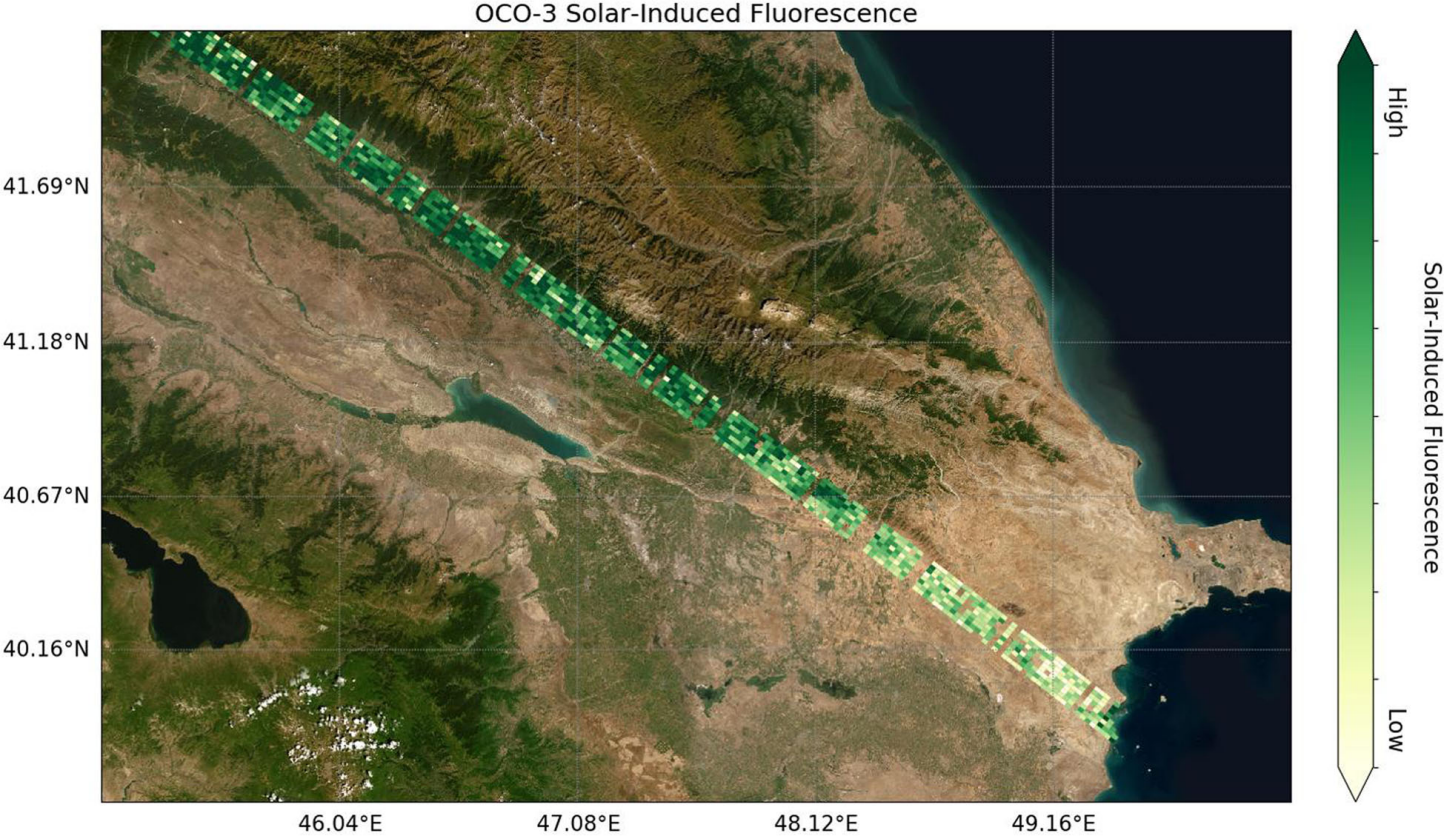
The first preliminary SIF data from OCO-3, showing a strip from the edge of the Caspian Sea inland into Azerbaijan, superimposed over a true color image. Vegetation near the Caspian Sea is steppe and grasslands but it becomes forested as it moves into the mixed broadleaf deciduous forests of the southeastern Greater Caucasus Mountains, located north of the Mingachevir Reservoir (near the figure center). Credit: NASA/JPL-Caltech

An early example of OCO-3 data (Fig. 23) depicts variation in SIF (shown in shades of green to yellow) over an area west of the Caspian Sea (seen on the right side of Fig. 23). Areas with less photosynthetic activity (semiarid steppe grasslands) are yellow to light green and areas with high photosynthetic rates are dark green. Regions of denser forest are seen in the background image as shades of dark green.

#### Hyperspectral Imager SUIte (HISUI)

HISUI is a contribution to the ISS from the Japanese Ministry of Education, Culture, Sports, Science and Technology (MEXT) and the Japanese Space Agency (JAXA). Launched on December 5, 2019, for a 3-year mission, it is the second hyperspectral imager on the ISS (Table 4) and differs from DESIS by being a full VSWIR spectrometer (Table 4) with 185 spectral bands over the 400–2500 nm range. The VNIR wavelengths are viewed at 10 nm spectral resolution (SNR, 450@620 nm), whereas the SWIR region is viewed at 12.5 nm (SNR, 300@2100 nm). HISUI’s pixel size is 20 m × 30 m (600 m^2^) within each 20-km swath. The data quality is expected to be sufficient to identify the presence and quantify the concentration of many plant biogeochemicals that characterize ecosystem trait complexes, and to show how these suites of signals change seasonally and inter-annually.

HISUI is expected to enable identification of land cover classifications at the levels of vegetation species and plant communities (Meerdink et al., 2019). Thus, it is expected to contribute to improved understanding of biodiversity patterns in the mid to low latitudes, and to better quantify high spatial resolution changes in land cover. Collaborators will have priority access for observations, priority downlink, and distribution at no cost for their requested areas. Archive data will be distributed by the HISUI project at no cost to science investigators who agree to share their outcomes with the HISUI project. The HISUI project will provide opportunities to propose research collaborations using HISUI data.

#### Earth Surface Mineral Dust Source Investigation (EMIT)

EMIT is a NASA Earth Ventures-Instrument (EV-4, 2017) selection for the ISS (Table 4) and managed by NASA/JPL. It will be a full VSWIR (0.380–2.51 μm) hyperspectral imager with a mission to map the minerology of arid regions across the globe. These are sources of dust that are propelled into the upper troposphere and lower stratosphere, affecting Earth’s radiation budget. Windblown dust causes large errors in calculating the Earth’s energy budget due to the wide range of albedos in the minerals from arid lands. Understanding the impact of dust on the planetary radiation budget depends on knowledge of the chemistry of the dust. For example, light-colored clays and carbonates that reflect more light contribute to cooling of the atmosphere, whereas iron oxides and silicates that are darker and absorb more solar energy contribute to heating of the atmosphere. This information is essential to support development of a more accurate model of the planetary radiation budget, necessary for climate change projections. The proposed EMIT instrument is expected to have ~ 300 VSWIR spectral bands with 30 m pixels in a 1240-km wide swath. In addition to mapping soil minerology, EMIT should provide good-quality data for ecological and agricultural applications, when time is available to collect non-primary sites after the primary objectives of this project are met. These data will be complimentary to HISUI acquisitions.

**Fig. 24:**
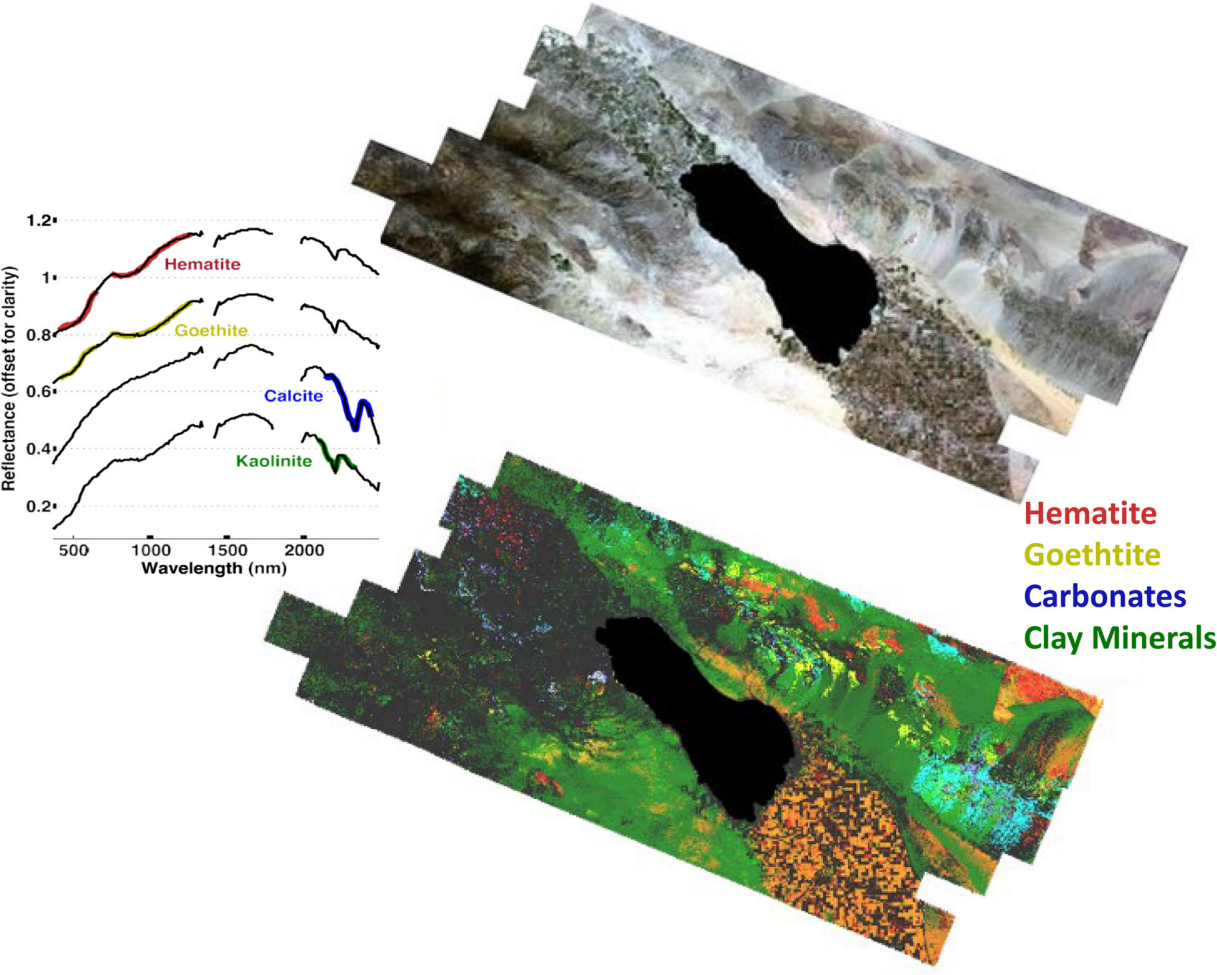
Illustration of mapping mineral exposures in arid and semiarid sites that are sources for airborne dust that gets into the upper troposphere and stratosphere, absorbing or reflecting soil radiation, thus affecting the radiation budget of the earth. While the true color image makes the area look to be composed of similar geologic minerals (being different tones of gray), but when locations of surface minerals are color coded and mapped, the complex surface minerology is evident. In this example, the hematite and goethtite minerals are dark and absorb more solar energy while the carbonates and clay minerals are light-colored and absorb less energy, and scatter energy back toward space. Data was acquired for this example on 03/31/2014 by the Airborne Visible Infrared Imaging Spectrometer (AVIRIS) over the Salton Sea, California, flown on the NASA ER-2 aircraft at 30 km altitude (Baradley et al. 2020)

Figure 24 shows spectral differences among minerals in an image from the Salton Sea area in southeast California. The upper panel shows the Salton Sea near the center of seven flight lines obtained by airborne AVIRIS (Advanced Visible InfraRed Imaging Spectrometer) data, providing a prototype of what EMIT will see from the ISS. The various tones of gray and light blue show that there is little vegetation in this semiarid desert region, consequently there is surface expression of the exposed weathered geologic formations. The center panel names several common soil minerals by color in the region, and the spectrum of related minerals is similarly color coded. The lower panel shows the complex distribution of these minerals in the area, with clay minerals dominating most pixels. Areas of yellow and red look to be mostly pure hematite or goethite outcrops, and the cyan color indicates mixtures of the carbonate and clay minerals. The goal of EMIT is to use these mineral signatures to determine the mineral composition of surface dust from arid sites and consequently improve estimates of solar energy absorption of the particles comprising dust clouds and dust storms.

## New advances in Earth observing satellites that are under development

### Joint NASA and Indian Space Agency (ISRO)

The NISAR (NASA-ISRO Synthetic Aperture Radar) is an ambitious joint mission for an L-band (24 cm wavelength, 3.20 GHz) polarimetric SAR provided by NASA, and an S band (9.3 cm; 3.20 GHz) polarimetric SAR provided by ISRO. The satellite has an expected launch in 2022 Table 5. The large SweepSAR antenna (Table 5) has advantages over traditional approaches, with its very low mass and large surface area that requires less transmitting power and a less complex array design (Freeman et al. 2009). In polar LEO orbit, it will collect all-weather day and night images of the Earth’s entire land and ice masses four to six times/month at high resolution, 5 m to 10 m (25–100 m^2^) pixel sizes. The focus of this mission is to monitor the Earth’s changing ecosystems, and its dynamic surface (e.g., surface deformation, landslides, earthquakes, and ice masses). It will monitor disasters and extreme weathers events, e.g., droughts, wildfires, floods, hurricanes, and even insect outbreaks to provide information for managing state and health in natural resources of the Earth, in terms of plant biomass and changing hydrologic processes, e.g., sea level rise, changes in glaciers and ice sheets, and groundwater changes. NISAR will monitor properties of significant interest to the ecological community such as sources and sinks of carbon and changes in biomass storage that will improve understanding of carbon uptake in woodlands, agriculture, wetlands, and permafrost systems.

### Surface Biology and Geology (SBG) mission

The SBG concept achieved the highest category recommended to NASA by the 2017 Decadal Survey (NAS 2018). This new mission is based on heritage from the HyspIRI (Hyperspectral and Infrared Imager) mission, the previous recommendation of the 2007 Decadal Survey (NAS 2007). The SBG mission (expected to be renamed before launch) is currently in the planning stage by NASA, to design, build, and launch a satellite mission in 2026 or 2027 for global measurements at near-Landsat spatial scale (30–60 m) with hyperspectral and thermal instruments. The plan (Table 5) calls for a full wavelength VSWIR imaging spectrometer covering the spectrum at 10 nm resolution between 0.350 and 0.400–2.500 μm to acquire “high-fidelity” measurements at 30 m spatial, having spectral sensitivities of > 400 for VNIR and > 250 for SWIR wavelengths to provide ≥ 5% accuracy. These instrument specifications are necessary to achieve the science goals of mapping species and community traits like deciduousness and leaf type (needle leaf or broadleaf), leaf area index, canopy volume and shape, and biochemical traits such as mapping plant chlorophyll and carotenoid concentrations, water content, foliar dry matter (including ligno-cellulose), leaf mass per unit area (Serbin et al., 2019), and other traits. The spectrometer should provide more highly resolved species differences for vegetation mapping and crop monitoring and should improve mapping accuracies for soils, minerals, and water quality. The Phase 2 SBG plan for a multiband thermal instrument includes 5 TIR bands from 8 to 12 μm and a 3–5-μm band, all with response sensitivity at 60 m spatial resolution. These measurements will provide surface temperature and emissivity measurements for global energy budgets, as well as volcano monitoring capabilities.

The Recommended Architecture made to NASA HQ on 7/15/2020 describes two freeflyer platforms: a dedicated wideswath VSWIR instrument at 30 m spatial resolution with a morning (10:30–11:00) polar LEO (@ $400–500 M) compatible with ESA’s CHIME; and a multiband thermal instrument supported by a VNIR camera for context, with a 935-km swath in LEO orbit for 3-day revisits. Two alternative architectures were offered (at an estimated cost of ~ 4× the recommended architecture): a single platform supporting both the VSWIR and TIR instruments, possible in 2027–2028; and a Constellation of SmallSats, with challenging calibration and validation issues. The recommended 2-platform architecture relies on collaborations and synergies expected between 2026 and 2032 with ESA, CNES, ISRO, and other space agencies. Other discussions relate to synergies with Landsat-10 (currently in design as Landsat-Next), ESA’s Sentinel-2 operational series, and ESA’s decision on Sentinel-8 for the proposed Land Surface Temperature Monitoring (LSTM) mission (2025 launch date) if selected as a companion to Sentinel-2 satellites, thereby supporting SBG and discussions between NASA and ESA on the complementary CHIME mission.

### Carbon Cycle Observatory (GeoCARB)

**Fig. 25:**
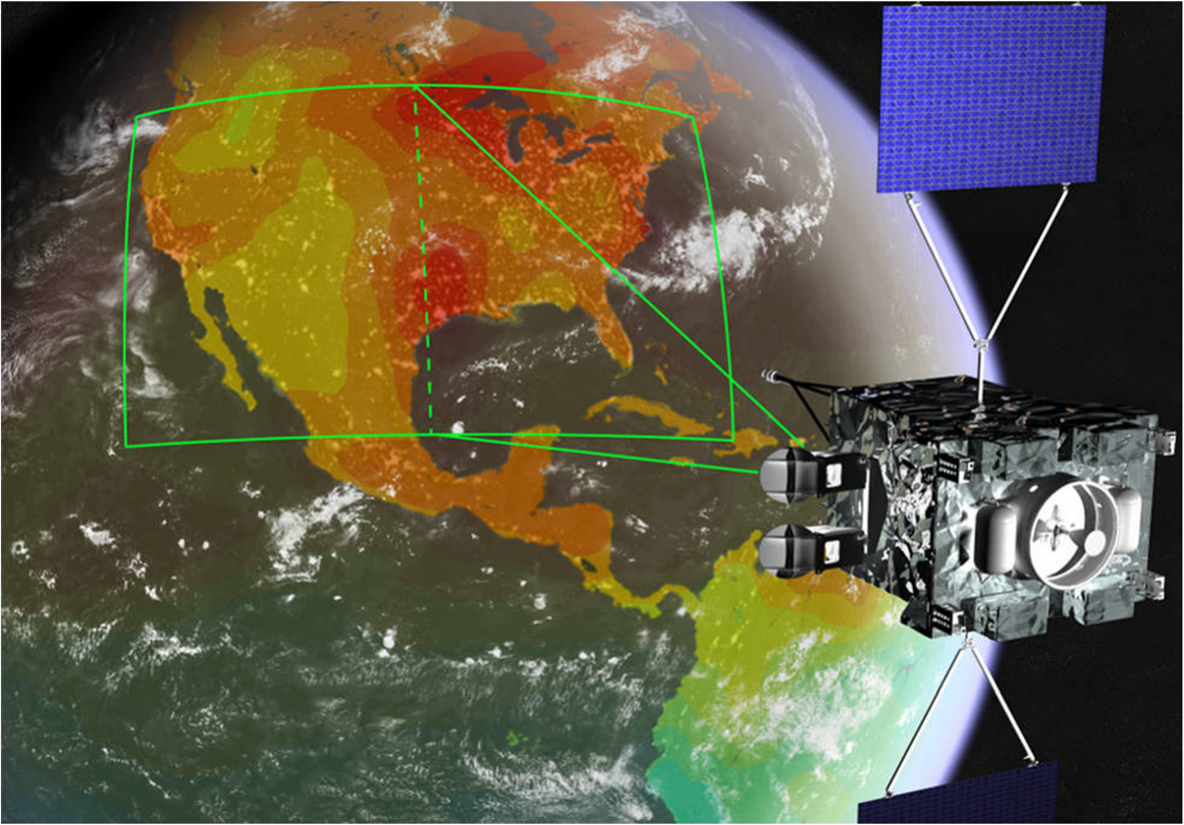
Artist’s rendition of the areal coverage of GeoCARB (Geostationary Carbon Cycle Observatory) being mounted on a commercial geostationary communication satellite operated by SES S.A. GeoCARB will be centered at 85° West longitude over the Americas and will make observations between 50° North and South latitudes. GeoCARB will acquire properties of atmospheric constituents including carbon dioxide, methane, and carbon monoxide at multiple times during the daylight hours, to improve understanding about the global carbon cycle and vegetation health. Figure from https://www.nasa.gov/feature/jpl/geocarb-a-new-view-of-carbon-over-the-americas

GeoCARB is a NASA geostationary satellite mission, led by Dr. Barrien Moore at the University of Oklahoma, with an expected launch in 2023 to detect atmospheric concentrations of CO, CO_2_, CH_4_, O_2_, and far-red SIF (Table 5). GeoCARB will provide data about the global carbon budget in relation to fAPAR, GPP, and SIF that will lead to improved models of Earth System processes. GeoCARB promises to improve understanding of the carbon cycle and monitoring of vegetation health on a commercial geosynchronous satellite in stationary orbit over North America, centered at 85° West longitude (Fig. 25, Table 5). The specifications for GeoCARB are still in review and so these characteristics are uncertain. Spatial resolutions are expected from 0.5 to 10 km and cover the continental USA. The abundance and distribution of carbon gases in the atmosphere are determined by a balance between photosynthesis and respiration. Carbon is exchanged between land, oceans, and the atmosphere, being transported by prevailing winds. These exchanges are best understood by making frequent, densely spaced observations of flux properties that only a geostationary orbit can provide. The GEO orbit allows multiple acquisitions per day (~ 2.5 h/scan) of very high spectral resolution measurements. Another advantage is that at the altitude (~ 35,000 km) of GEO satellites, it will measure total upwelling radiance that is an aggregate of both atmospheric and land surface processes that scatter, absorb, and reflect photons, which can be modeled with modern radiative transfer models. The baseline mission is to produce accurate column-averaged mixing ratios of CO_2_, CH_4_, and CO (Polonsky et al. 2014). GeoCARB will measure reflected NIR and SWIR wavelengths centered at the 0.763 μm (O_2_-A atmospheric absorption feature) and at the weak CO_2_ feature at 1.611 μm. Additional measurements will be made at the strong CO_2_ absorption feature at 2.06 μm to obtain a column integrated CO_2_ dry air mixing ratio (XCO_2_), and at 2.32 μm for XCH_4_ and XCO.

### Plankton, Aerosol, Cloud, ocean Ecosystem (PACE)

NASA’s Plankton, Aerosol, Cloud, ocean Ecosystem (PACE) mission is a polar LEO observatory with three instruments scheduled for launch in late 2022 that will extend and improve NASA’s more than two-decade record of satellite observations of global ocean biology, aerosols, and clouds (Table 5) (www.gsfc.nasa.gov). PACE builds on the heritage of several NASA satellites including the Coastal Zone Color Scanner (CZCS), the first ocean color satellite, a multispectral radiometer launched in 1978; the Sea-viewing Wide Field-of-view Sensor (SeaWIFS), a multispectral imager with 8 VNIR bands (416 to 865 nm) that operated from 1997 to late 2010 in a noon overpass with 4 km resolution ocean data; and the MODIS on Aqua and the VIIRS, described above.

The primary instrument on PACE is the hyperspectral Ocean Color Instrument (OCI) with continuous 5 nm spectral measurements from the UV (0.340 μm) to the NIR (0.890 μm) plus seven discrete bands between 0.940 and 2.260 μm, acquired at 5 nm resolution. It is supplemented by multi-angle Polarimeters. The OCI has a 2663 km swath that allow a 1-day repeat cycle for global ocean measurements, with 1 km^2^ pixels at nadir. The polarimeter radiometers measure polarized light backscattered from clouds, aerosols, and ocean, thus enabling better understanding of how light interacts with these components of the earth system.

By measuring the distribution of phytoplankton and algae that sustain the marine food web, PACE will advance understanding of ocean health. PACE continues systematic records of key atmospheric variables associated with air quality and Earth’s climate. Although designed primarily for ocean studies, OCI data will be collected over land, routinely processed, and atmospherically corrected. Therefore, its data should be of interest for terrestrial ecosystem researchers who have used MODIS data from Terra or Aqua in their studies.

### Himawari 8, GOES-16 and GOES-17, and MTG-1

While weather satellites are not necessarily of interest to the ecological community, these GEO satellites uniquely provide diurnal imagery of the land surface at useful ecological scales from about 1/3 to 1–2 km, depending on the band and the instrument. The ability to monitor crop (or forest) production 4 to 12 times per hour and up to 288 observations in 24 h throughout a growing season, provides multiple opportunities to understand both carbon and water fluxes at field-scale to regional-scale, with pixels about the scale of flux tower footprints (Table 5). The third-generation GEO satellites have improved spatial, spectral, radiometric, and temporal imager resolutions and the lightning imagers allow analysts to follow weather systems from their origin. The new sounders improve the 3-D probing of the atmosphere with data from the UV, VIS, and NIR-TIR. Together, the imagers and sounders are expected to greatly improve weather forecasting, both near-real-time (nowcasting) and extended range forecasting, with data providing new inputs to Numerical Weather Prediction Data Assimilation Models. The enriched data from these instruments will lead to more quantitative data products, contributing to advances with these systems. Today, JAXA has been flying one of the third-generation systems since 2014, NOAA has been flying two systems since 2016 and 2018, and EUMETSAT will fly three systems at a time, one MTG-I imager for the full disk that covers from western Asia to the western Atlantic and a second imager that covers Europe and North Africa, in 2021, and the MTG-S sounder in 2023. These four next-generation GEO full-disk weather satellites cover the Earth several times per hour, providing unprecedented ability to follow storms and other severe weather from their origin to the end.

The Japanese Space Agency (JAXA) was the first to fly a third-generation geostationary satellite in 2014, the Himawari 8 (Table 5), that carries the Advanced Himawari Imager (AHI), a 16-band multispectral imager similar to NOAA’s Advanced Baseline Imager (ABI) on the Geostationary Operational Environmental Satellite (GOES) system (GOES-16 and GOES-17) and with similar spatial resolution. These three satellites (Himawari 8, GOES-16, and GOES-17) serve as the first group of new third-generation GEO weather satellites that are part of the international consortium to obtain next-generation weather data covering the whole Earth.

The two NOAA GOES satellites, GOES-East (GOES-16, centered at 72.2° West) and GOES-West (GOES-17, centered at 137.2° West), cover the regions from the eastern Atlantic to the western Pacific and from pole to pole (Table 5). They host five new and updated instruments. The instrument most useful for ecological/environmental research is the ABI and the Earth Observation Global Lightning Mapper (GLM). The ABI has 16 spectral bands covering the VIS (blue and red) region, with 4 NIR and SWIR bands, a mid-IR band, and 9 bands in the TIR wavelength region. This is nearly as many bands as the polar-orbiting VIIRS, and with the spatial resolution of MODIS. The planned polar-orbiting ocean PACE will have similar resolutions in its solar reflectance and thermal bands. NOAA reports that “It [GOES] provides three times more spectral information, four times the spatial resolution, and more than five times faster temporal coverage than the previous [GOES] system.” (https://www.goes-r.gov/spacesegment/abi.html).

**Fig. 26:**
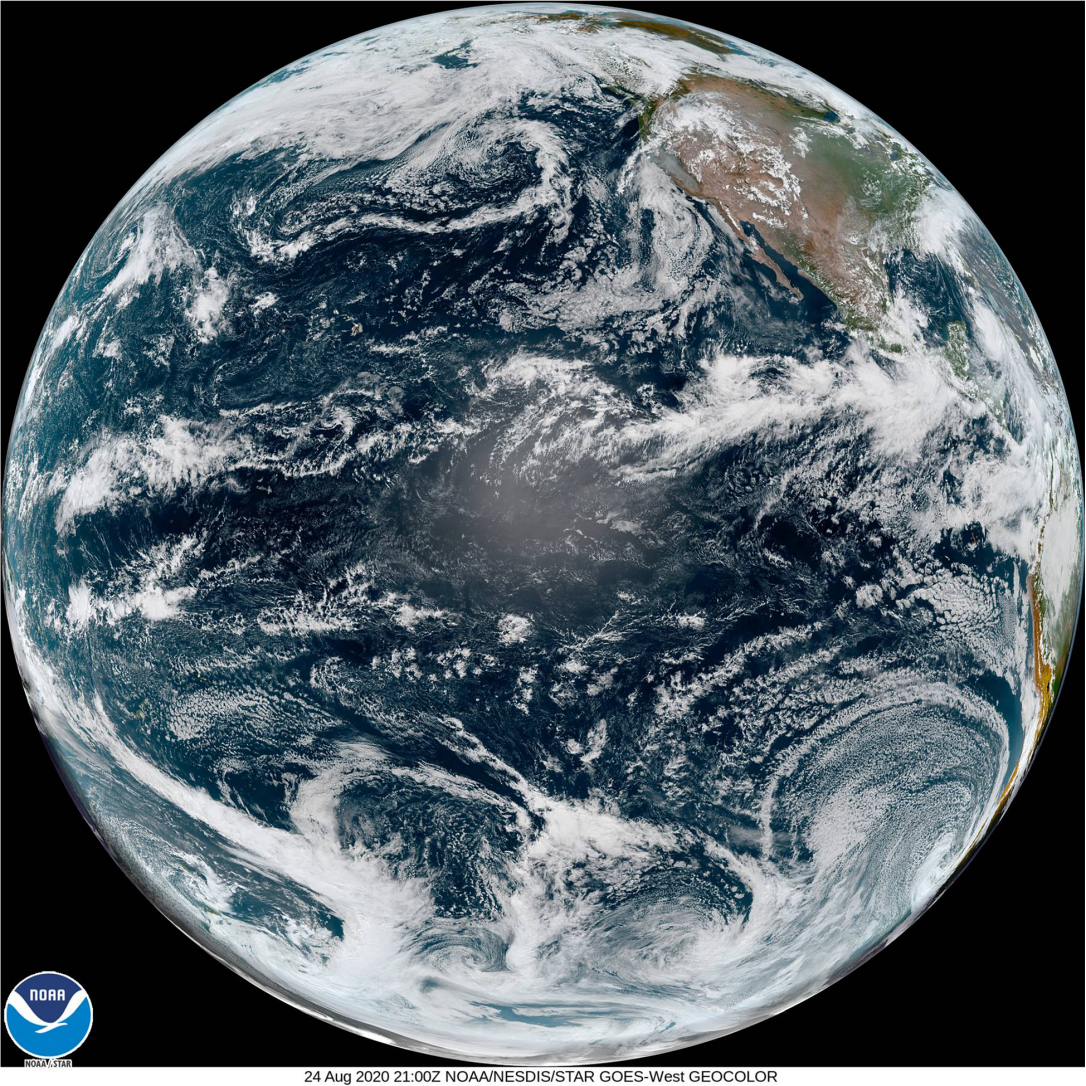
This GOES-West image stretches from the western Pacific ocean to the western North America, and from just south of Alaska southward to Central America and the far western margin of Equador and Peru in South America. These disks map the Earth every 10 min at spatial resolutions from 500 m to 2 km and subsets are mapped even more rapidly. The data quality makes these data an exciting time series for study of diel processes

NOAA’s GOES 16 and 17 have multiple collection modes (Table 5). In the continuous full disk (mode 4), the ABI scans a full disk of the Western Hemisphere from pole to pole, producing an updated image every 5 min. In the flex (mode 3), the ABI produces a full disk every 15 min (Fig. 26), and a CONUS image (resolution 3000 km × 5000 km) every 5 min, and two mesoscale domains (resolution 1000 km × 1000 km) obtained at the satellite sub-point every 60 s or one sub-domain every 30 s. Mode 6 is a 10-min flex mode that became NOAA’s default operating mode for GOES-16 and GOES-17 in April 2019. It provides a full disk image every 10 min, and a CONUS (GOES-16) / PACUS (GOES-17) image every 5 min.

**Fig. 27:**
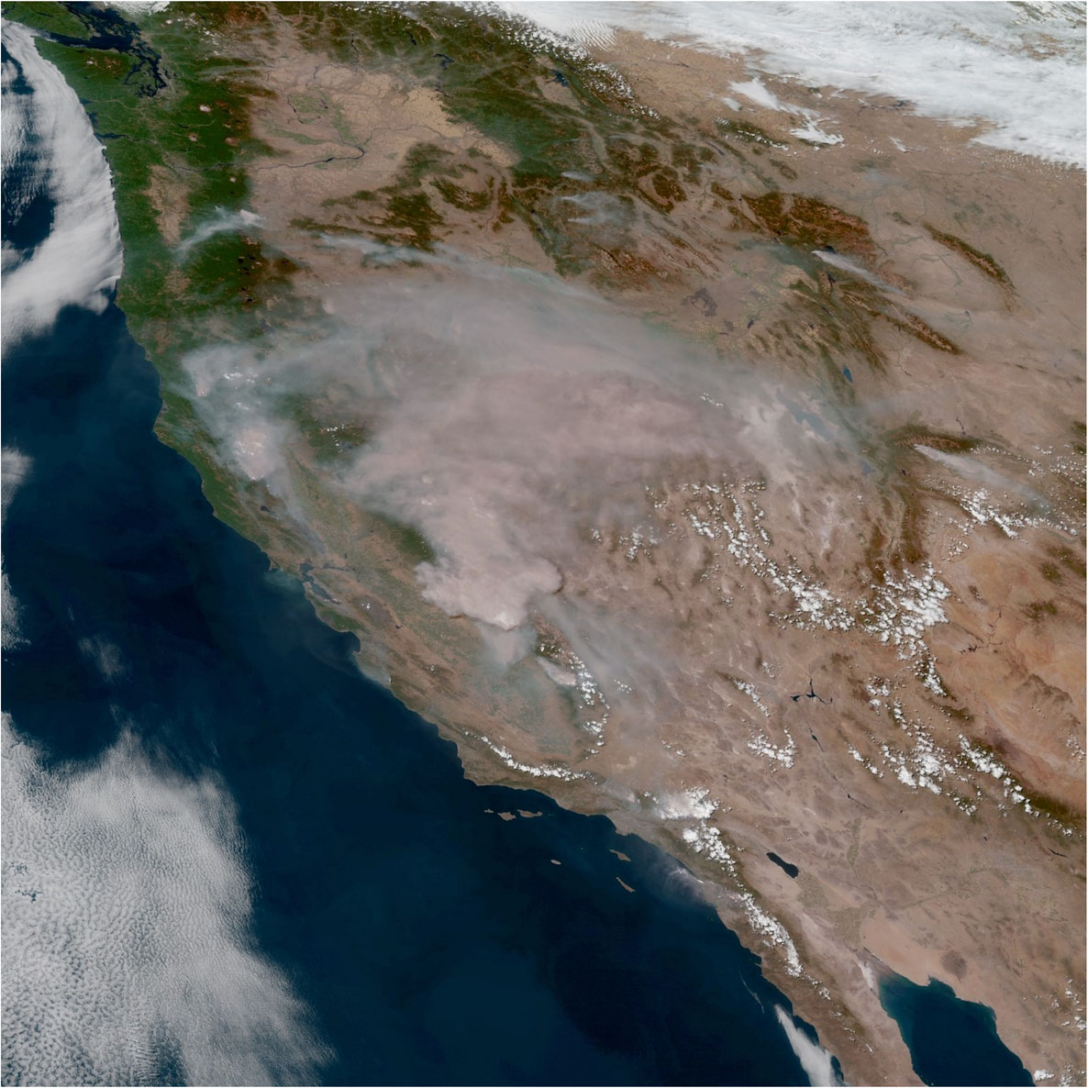
This zoom image from the GOES image Fig. 26 shows the western United States in more detail. The image covers from the Pacific Ocean to the edge of the Great Plains, east of the Rocky Mountains. There are numerous fires at this time in the west

Figure 27 provides an enlargement of the data in Fig. 26 to see more clearly the smoke plumes from fires at this time, which are tan colored, compared to clouds that appear white in this image. The area near the top of the image includes most of Washington and extends south into northern Mexico, including Baja, at the bottom of the image. All but the smallest of the Channel Islands are seen off the coast of southern California. The semicircle of low white clouds, slightly northeast of the Channel Islands, highlights the location of the southern end of the Sierra Nevada Mountains where they curve around (Tehachapi Mts.) and meet the southern Coast Ranges. The dark blue elongated lake, north of the Gulf of California is the Salton Sea, a saline rift lake on the San Andreas Fault.

The great conifer forests of the Pacific northwest are dark green with two arms running south along the Coast Ranges and along the Cascade Mountains in Washington and Oregon and the Sierra Nevada Mountains in California. These contrast with the sparser vegetation seen over much of the semiarid region that have a strong spectral component coming from exposed soil and geologic minerals. The smoke plumes from these larger fires are being blown east and south, toward the southern end of the Rocky Mountains. The largest and densest smoke plume is from the Creek Fire in the Sierra Nevada Mountains and it is being blown in two directions, one arm toward the south along the San Joaquin Valley, and the other toward the southern Rocky Mountains. This fire erupted on September 4 and by October 8 it was only 49% contained and had burned 1339.1 km2 area. There are numeroussmaller fire plumes visible throughout the image.

The ESA GEO Sentinels 4 and 6 (described earlier) will fly with the next-generation EUMETSAT GEO weather satellites; the Meteosat Third Generation GEO (MTG-I (imaging) series) is planned to fly for 20 years, starting in 2021 (Table 5). Their proposed configuration is to fly two MTG-I satellites together, one collecting more rapid data (2.5 min) over Europe and North Africa and the other the full disk (10 min) of the Earth centered over Europe and Africa, with a third MTG-S (sounding) satellite (Table 5). The imaging sensor on MTG-I will be the 16 band Flexible Combined Imager (FCI) along with a lightning imager (LI). The VSWIR bands will record 1 km resolution and the remaining 8 TIR bands will be recorded at 2 km. The MTG-S, to be launched in 2023, will include an interferometer, InfraRed Sounder (IRS), and the Copernicus Sentinel-4 Ultraviolet Visible Near-Infrared (UVN) sounder.

## Summary and relevance to the ecology community

Dr. Michael Freilich stated in his final presentation to the global environmental community for the 50th anniversary of Earth Day in April 2020 (https://www.youtube.com/watch?v=QdSCkLrC4Fg): “…Earth observations are made with more detail and looking at more processes and variables than ever before…Our scientific satellites are taking the pulse of the planet everywhere and all the time.” We have compiled and described a number of spaceborne instruments and satellites with (mostly) open and free data policies that can be utilized in many productive ways by the environmental/ecological communities. Table 1 provides a list of satellite instruments organized by type of spectral measurements—panchromatic, multispectral, hyperspectral, thermal, LiDAR, and radar—which are ordered from finer scale to coarse scale. Tables 2, 3, 4, and 5 provide descriptions of each of the satellites discussed whether operations are historic, current/near past, current, or forward looking into the 2030s: for NASA (Table 2); ESA (Table 3); prototype instruments on the International Space Station (Table 4); and geostationary and coarse scale polar satellites (Table 5). All of these collect data relevant to topics of interest to different groups of ecological and environmental users. We provided exemplar publications that show typical uses of these data in the ecological and environmental communities. Table 6 organizes these missions according to type of measurement made (scale of data from fine resolution (left) to coarse (right) and by column for polar oriented sun-synchronous satellites and those in geostationary or other low-inclination angle orbits (e.g., 51° or 66°), and by launch dates and operation period, moving down the page. Information about these many satellites will help readers find all instruments with similar spectral/spatial bands or those in different time periods that potentially could be used to provide alternate or harmonized analyses by combining sources of data for studies that fill in missing time series data or extend data backwards to earlier dates.

Table 6 organizes the satellites by date of launch and operations. The first time-interval presented is referred to as the “Archive” period covering the most recent two decades (2000–2020) and describes game-changing instruments that acquired measurements during that period. Whether or not each is still active in the current half decade (2021–2025) is noted (* = expected to be available; # = continuation uncertain) and whether data collections are limited to a sampling design ($) or are measured everywhere. This Archive category provides current users of remote sensing data with past instruments because of many requirements for time series analyses.

Note that at the plot level (≤ 500 km^2^), the archived global datasets are available from Landsats 7 and 8 and Sentinel-1 and Sentinel-2. Archives for data collections in “sampling mode” include the EO-1 (ALI and Hyperion), Landsats 1–5, ASTER, CHRIS-PROBA, and VENμS. At the Local scale (~ 1 km^2^), these instruments were joined by the LEO operations mission Sentinel-3, the sampling mission PRISMA, and by five non-sun-synchronous (low-inclination angle) missions hosted on the ISS (DESIS, ECOSTRESS, GEDI, OCO-3, and HISUI). When moving up to the Landscape scale, two of the sampling missions drop out (ASTER and CHRIS-PROBA), but new global datasets available at this scale are Terra, Aqua, Suomi NPP VIIRS, Sentinel-3, and MERIS. At the regional scale (100 km^2^), TROPOMI is added to global observations but the EO-1/Hyperion drops out due to its narrow 7 km swath, as does VENμS, but the two US NOAA geostationary weather satellites (GOES-East, GOES-West) are added. At the next level, for national scale or for large regional studies (1000 km^2^), the remaining sampling missions drop out (Landsats 1–5, EO-1/ALI, and PRISMA), as do the ISS-hosted observations. For large-scale global observations (≥ 0.25°), only Terra, Aqua, VIIRS, and MERIS remain, as well as GOES-East and GOES-West, and Himawari, for Asia and the western hemisphere.

Moving to the current half decade (2021–2025), we list what is available at these same study scales. First, we note that plot level observations continue with a smaller satellite set: Sentinel-1 and Sentinel-2 and Landsat-8, now joined by Landsat-9; however, L-8 has already exceeded its expected 7-year mission life, so its continuation is uncertain. Also uncertain at this time period for plot-level measurements is the continuation of ASTER. At local scales (1 km^2^), PRISMA, EnMAP, Biomass, and FLEX join this small set of satellites, and the set of ISS-hosted instruments changes with the addition of EMIT and the completion of ECOSTRESS’s mission. At the landscape scale (10 km^2^), EnMAP and Biomass join PRISMA to provide sampled datasets whereas global observations continue with Landsat-8 (if operating), L-9, and Sentinel-3 and Sentinel-5; these are now joined by FLEX, and new global observation capabilities provided by NISAR and PACE and the polar weather satellites NOAA 20 and 21 and the METOP-SG-A1. Note that four missions cannot be counted on to continue during this time period: Landsat-8, Terra, Aqua, and Suomi NPP. Also, geostationary measurements are added by the Sentinel-4 atmospheric mission and Sentinel-6 ocean topography mission. This same group of satellites provides measurements suitable for Regional and National studies (100–1000 km^2^), joined now by TROPOMI (global) and WildFireSat (Canadian region). And this group also provides large regional or national coverage (1000 km^2^), with the two GOES satellites joined by geostationary satellites Sentinel-4 and Sentinel-6, but the ISS instruments drop off as do the sampling instruments PRISMA and EnMAP. At the continental to global scale, only three satellite missions can be expected (PACE, Sentinel-3, and Sentinel-6) plus the METOP-SG LEO weather series, in addition to the GOES weather satellites, since Aqua, Terra, and Suomi NPP may no longer be operational at that time.

Things change rapidly in the second half of this decade (2026–2030). NISAR joins Landsat-9 and probably Landsat-10 (currently designated as Landsat-Next), and the Sentinel-2 series to support plot-scale studies. At the local scale, notice that the ISS missions are now completed, so this group includes the satellites in the plot-scale group joined by the new missions, Sentinel-8 (LSTM) and SBG and potentially CHIME. This satellite set can also address landscape observations, along with the Sentinel-3 series, and most likely the two Earth observers (Biomass and FLEX), at least for part of this time period. For regional observations, this group of polar LEO satellites is complemented by TROPOMI (if continued), the geostationary satellites, GEOCARB and NOAA’s GOES-East and GOES-West (GOES-16 and GOES-17). This group also serves national or large regional observational needs, in addition to geostationary atmospheric satellites, Sentinel-4 and Sentinel-7. Continental scale observations are possible with mosaics built from SBG and Sentinel-3 series, in addition to the geostationary satellites (for their large view).

Beyond this 2020 decade, few satellites are confirmed to be in orbit, although we expect continuation of the Landsat program with Landsat-10 and beyond, given its core role in the National Land Imaging Program (https://www.usgs.gov/land-resources/national-land-imaging-program/). The Sentinels are operational satellites for the European Union and there is an assumption that most or all will be continued. Specifically, Sentinel-1–3 have commitments to build copies out to versions E and F, until the mid-2030s. Sentinel-4 and Sentinel-5 are part of the weather satellites and consequently are likely to be continued as is the ocean topography mission Sentinel-6, which is extending more than 20 years of sea level height observations. Sentinel-7, the CO_2_ monitoring mission, is expected to last at least to 2038 before a replacement is likely to be necessary. NASA’s SBG hyperspectral satellite should be flying into the 2030s and supplemented by the Sentinel-10 which, based on today’s priorities, is likely to be CHIME. In fact, NASA and ESA are in discussions on how SBG and CHIME can be used together to reduce the latency between measurements and other synergies, such as with LSTM (if selected for Sentinel-8) and Sentinel-2 (and with SBG and CHIME). Harmonization of Landsat-8 and Sentinel-2 data are already available. When L-9 joins L-8 and Sentinel-2A and Sentinel-2B, it is likely to have ~ 2-day repeat coverage of the Earth at mid-northern (and -southern) latitudes, focused on land areas, potentially facilitating 20 to 30 m spatial resolution coverage of crop phenology and thus supporting global food security concerns. PACE is expected to be operating in the 2030s.

### Data synergies

Many combinations of data greatly enhance the information that can be extracted relative to single sensor’s data (Shiklomanov et al., 2019). One example is combining various multitemporal data from multispectral Landsat or Sentinel-2 data, and thermal imagery from ECOSTRESS or LSTM data, or hyperspectral (PRISMA, EnMAP, HISUI, EMIT, SBG, or CHIME), and 3-D structural information from GEDI LiDAR or Biomass radar sensor into a supervised plant community/species level classifier using the R-program “Random Forest” (https://cran.r-project.org/web/packages/randomForest/index.html). An example of synergistic uses of different data combinations is shown in Fig. 18. Classifications can generally be improved by combining different types of data together (e.g., calendar dates that span a growing season; Tang et al., 2016), or combining spectral data (multi- or hyper-) with structural information, such as an active LiDAR or radar sensor, or considering information in different spectral regions such as UV, VIS, SWIR, and TIR imagery or imagery from different view angles. With the large number of new and continuing spaceborne instruments combined with today’s computing environments, it is becoming far easier to consider using data from multiple instruments together.

Landsat and Sentinel data can be used to “sharpen” MODIS or VIIRS data to get higher spatial resolution global coverage for vegetation or land use maps. Landsat and Sentinel data can be used to fill in a time series with less frequently available hyperspectral data such as PRISMA, EnMap, DESIS, or HISUI to map a larger area. Or the hyperspectral data can be used to train the more frequent multispectral imagery to retrieve some biochemical information. Or Landsat and Sentinel-2 can be linked to GEDI LiDAR or Sentinel-1 radar to improve estimates of biomass or disturbance dynamics. Since all of these instruments have pixels in the 20–30 m range, they should provide a more complete understanding of biochemical and physiological traits over landscapes, along with their 3-D structural properties. Hyperspectral data can retrieve quantitative estimates of photosynthetically important pigments, including chlorophylls *a* and *b*, caroteins, and anthocyanins (Eli et al., 2019). Datasets from various satellites can be combined using radiative transfer models (RTMs) or RTMs with empirical regression models, to enable retrieval of quantified measures of leaf and canopy water content, which are useful in determining crop stress or to monitor regional drought conditions. Measures of leaf mass/area (LMA) can be correlated with plant productivity rates. For example, low LMA values are generally correlated with high plant productivity, along a continuum that extends to high values of LMA, which is correlated with plant/crop/ecosystem stress conditions and stress tolerance. Another synergistic use of some multispectral or hyperspectral image data is to sharpen geostationary weather satellite data (e.g., NOAA GOES, JAXA Himawari, or EUMETSAT MTG, with visible bands at 500 m pixel resolution) to create higher spatial resolution data products that can be used to measure and characterize ecophysiological processes that change over diurnal time scales, particularly more accurate estimates of evapotranspiration.

When considering synergies among available satellite datasets, the first priority is to define the region of study and its location boundaries, so that a search can be performed on appropriate websites. In the first half of the 2020 decade, ecologists interested in contemporary, space-based data to support their land surface-based studies, may be interested in acquiring similar data types from domestic and international sources to obtain sufficient data for a time series analysis (e.g., growing season, annual, or multi-year). Or, they may be interested in obtaining the largest number of data types (VNIR, VSWIR, LiDAR, radar, thermal) to complete a more complex analysis like a plant classification study at their study site(s) using machine learning or other AI techniques.

Table 6 provides a way to think about available sensors at different scales from the plot and local to the global. It provides sensors in both LEO orbits and those in GEO or other non-sun-synchronous orbit, e.g., from the International Space Station. Many sampling missions have opportunities to request data collections at specific sites and times that would enable coordination with ground measurements if time is available on the satellite. Announcements are generally available at the program’s web site. Project data for these instruments are available on open web sites for downloading, thus data with specific measurement characteristics, acquisition time (day, day of year), and land cover type can be identified and used to test analytical methods or for exploration of ideas. Commercial satellite data are often available (at cost) to provide a high spatial resolution baseline data (< 10 m). Or other sources of regional data may be available, for example, the US Dept. of Agriculture collects high spatial resolution summer data in three- and four-band (VNIR) digital camera data for the National Agriculture Imagery Program (NAIP), at high spatial resolution, up to 25 cm pixels. Heritage data may be useful for the purpose of establishing a baseline or for spatial sharpening, e.g., Sentinel-1, Landsat-7, Terra ASTER, MODIS, and others (e.g., CHRIS-PROBA and EO-1’s Hyperion and ALI data), and their archives, especially when multiple instruments are combined, can provide a multi-decadal time series.

For regional contexts, higher spatial resolution satellite types (with 10–30 m pixels) can be paired with wider swath imagery such as VIIRS on Suomi NPP and NOAA-20, Sentinel-3’s Ocean and Land Colour Instrument (OLCI), the anticipated EE-8-FLEX synthesis products, and PACE’s Ocean Color Imager (OCI). Terra or Aqua MODIS instruments may still be operating through 2020–2025 (they are near end-of-life) or their heritage products combined with either of the current VIIRS instruments or Sentinel-1A and Sentinel-1B for surface topography. Satellite products are acquired at different repeat schedules, some within a few days and most within a month, and most have midmorning acquisitions, except as noted in sections above. All of those instruments provide spectral information that can be used for generalized land cover descriptors (e.g., land cover/land use category, LAI, and vegetation indices), and some provide more specific vegetation descriptors, e.g., plant traits (evergreen or deciduous, needle leaf or broadleaf, LMA), phenologic timing, and biochemical constituents (pigments, water, cellulose/lignin, nitrogen, etc.). With detailed data on plant traits and structure, various biodiversity indices (alpha, beta, gamma diversity indicators) can be derived from several current LEO spectrometers, including PRISMA, EnMAP, and three instruments on the ISS (DESIS, HISUI, and EMIT) for select sites. Vegetation and topography structure information can be integrated with the spectral information using GEDI on the ISS, Sentinel-1, or the future BIOMASS mission. All of these can be utilized in their native resolutions or aggregated at landscape (10 km^2^) and regional (100 km^2^) scales. Also expected to be available are ESA’s TROPOMI atmospheric products (on Sentinel-5P), including far-red SIF retrievals (FLEX and GeoCARB), and forest fire emissions, temperature, and fire perimeter data, data from Canada’s WildFireSat. At coarse spatial scales, polar weather satellites like NOAA-20 and the METOP-SG series, including the geostationary satellites (Sentinel-4, Sentinel-5, Sentinel-6; NOAA’s GOES-16 and GOES-17; Japan’s Himawari 8 and 9; and ESA’s future METOP-MTG and Sentinel-7 satellite), can be drawn upon to address global land, atmosphere, and ocean processes.

As planning extends into the next decade, there are fewer historic and current satellites still operating, and even currently planned satellites may be past expected life (Table 6), but there will be new satellite instruments that continue data collections deemed societally important by governments and user communities. It is likely that Landsat-10, SBG, and Sentinel-10 (if CHIME) will offer unprecedented opportunities for frequent coverage of global “wall-to-wall” hyperspectral measurements over land areas and coastal environments (waters < 50 m deep) that support quantifying both critical climate change impacts and threats to sustaining natural environments.

### What analytical tools are available?

Table 7 provides several open source packages and programs for analyzing remote sensing data today and more are being added all the time. This list is not exhaustive, and more closely represents examples that the authors know. Of course, more fully functional commercial packages are available but often expensive. Most web sites for the satellite instruments cite software that the science team has developed for analysis of that instrument.

**Table 7:**
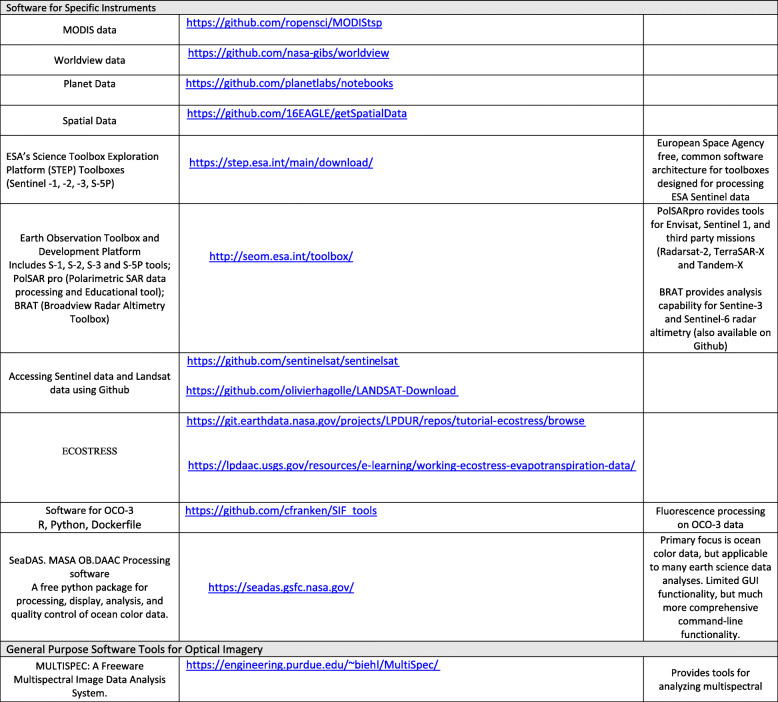

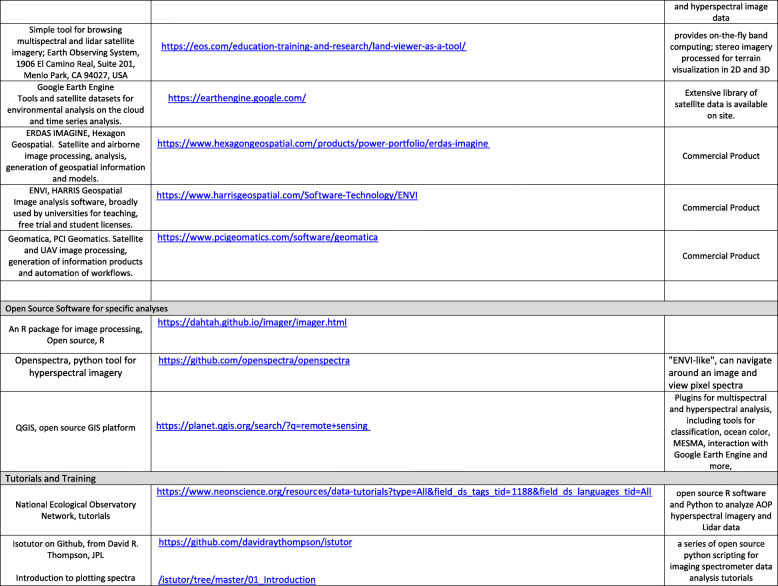

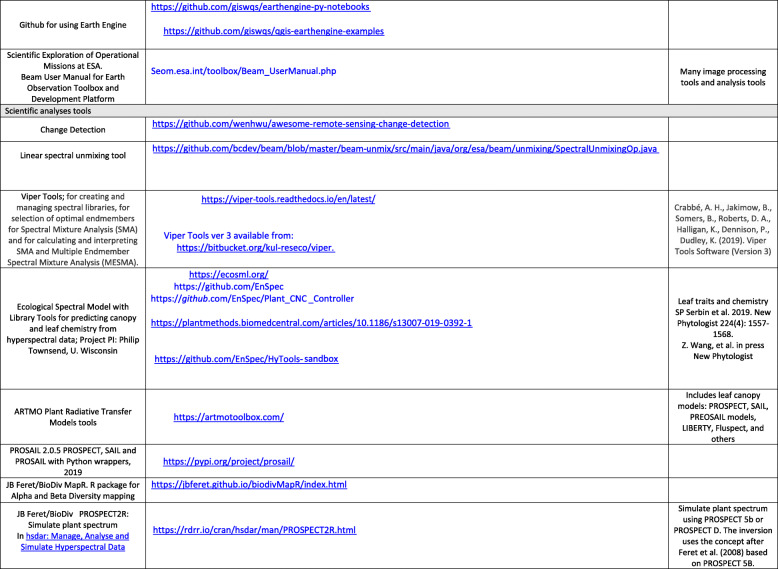

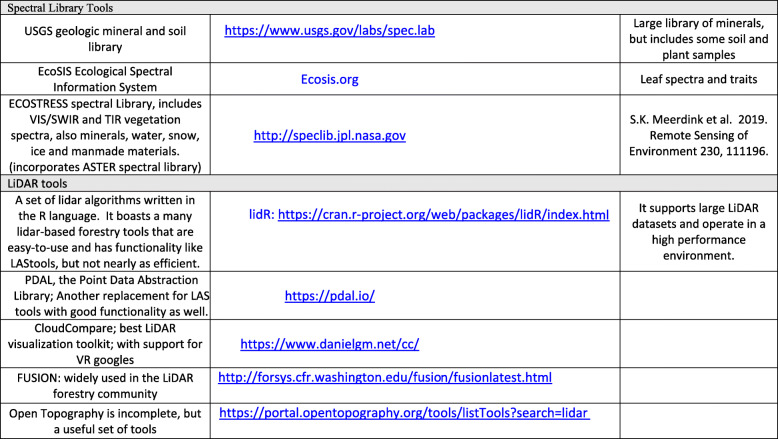
Examples of Open Source Programs for Analyzing Satellite Data

## Conclusions

The current expectation for ecological/environmental data is promising for an abundance of space-based open access global observations over the next two decades. This relies on an unprecedented array of Earth observing platforms and instruments that represent significant new technical capabilities in terms of the frequency of overpasses, number of wavelengths, the parts of the electromagnetic spectrum measured, the increased spatial resolutions, and the sensitivity and accuracy of the derived data products. At present, there are more satellites whose products are pledged to be available from public sites following a policy of “free and open” data than has ever happened before. The suite of technologies reviewed in this paper encompass a wide range of Earth observing data, with new information that is relevant toward understanding widespread changes in Earth conditions due to warmer temperatures, global changes in precipitation patterns, continued conversion of earth resources to human uses, and invasive species and biodiversity loss, including a long list of natural disasters (wildfires, landslides, earthquakes, tsunamis, floods, hurricanes, volcanic eruptions, drought, crop failures, etc.). The technical capabilities are moving analysis from inference of most processes based on a single set of measurements (e.g., one Landsat scene) made from one type of instrument (e.g., OLI or TIRS), to quantification and assessment of multiple data sources that address problems from different perspectives, concerns, and directions, and thus provide a wider range of information that construct a more holistic view of a problem.

We do not cover all or even most of the Earth observing instruments that will be flown in the coming decade (Figs. 4 and 7), as we limited this review to instruments of greatest interest for ecological studies, primarily for land terrestrial applications. The optical imagers are of primary interest in this review, because they provide land cover mapping at new spectral-spatial resolutions, and the widespread use of such data for process-based information primarily photosynthesis, evapotranspiration, and derived estimates of Gross Primary and Net Primary Productivity, GPP and NPP, respectively. We provide examples of several new hyperspectral imagers that will be able to obtain more detailed spectral information for identifying surface chemistry than the previous generation of multispectral instruments, thus detecting and discriminating different photosynthetic pigments at 30 m pixels, and detecting other soil and canopy components that influence the C, N, and water cycles. New technologies are enabling new measurements, such as SIF from vegetation, a very exciting development. Thermal imagers provide information on surface temperatures and emissivity but are also available for probing temperature-dependent processes. New multiband TIR imagers can obtain better calibration for increased accuracy at higher spatial resolutions than in the past. We included some radar imagers that will provide finely resolved topography and information about the three-dimensional structure of the surface, from which aboveground biomass is calculated. We also included the GEDI LiDAR sensor, as it is the first spaceborne instrument focused on ecological structures for terrestrial applications, collecting vertical distributions of forest structure at 30 m pixels. These LiDAR and radar products will provide detailed information to produce more accurate three-dimensional maps of the Earth’s land and ice surfaces, leading to better understanding of the rates of change in the terrestrial surface and in the cryosphere.

In the coming decade, we will retrieve a new level of quantitative information about chemical composition and concentrations in the air, land, and water of the Earth’s surface that will be collected concurrently with detailed optical, thermal, radar, and LiDAR imagery. This data will fill gaps in understanding, reduce uncertainties in Earth system models, and undoubtedly lead to significant advances and unexpected breakthroughs in understanding the Earth as a system and its processes. This decade will provide unparalleled opportunities to monitor landscapes and determine their state of recovery after disturbance or state of health, providing opportunities to better understand deficiencies in current management and aid design of new management practices. Instead of analyzing an “image,” users will be analyzing “data cubes” that are composited together for a region and time period from multiple different imaging instruments, such as hyperspectral imagery, LiDAR, and multiband TIR and atmospheric sensors adding SIF, carbon gases, water vapor, and other measurements. Most of these space-based data types will be delivered in terms of standardized products, greatly simplifying their usability and enabling more rapid processing and interpretation of critical information not possible from traditional methods. And making these data open to a wider range of users should facilitate studies across the globe and allow more synthetic studies to better understand how solutions to environmental and ecological questions vary with ecosystems, culture, climate, geography, and history.

## Data Availability

Since this is a review article, no original data were used.

## Abbreviations

ABI: Advanced Baseline Imager on the GOES-R satellite series for geostationary operational environmental monitoring
AHI: Advanced Himawari Imager, flown on Japan’s Meteorological Agency’s geostationary satellite, Himawari-8
ALEXI: Atmosphere–Land Exchange Inverse, an evapotranspiration model
ALI: Advanced Land Imager flown on EO-1, prototype for Landsat 8
Aqua: Afternoon (~13:30) satellite, leading the “A-Train” constellation in the NASA Earth Observing Satellite (EOS) program with six instruments, including MODIS
ASTER: Advanced Spaceborne Thermal Emission and Reflection Radiometer on the Terra platform
ATLAS: Advanced Topographic Laser Altimeter System on ICESat-2 satellite
A-Train: “A-Train” constellation (Aqua, CloudSat, CALIPSO, PARASOL, and Aura) flying in close formation with each other to provide near simultaneous atmospheric measurements, improving quality of measurements.
AVIRIS: Advanced Visible InfraREd Imaging Spectrometer, an airborne hyperspectral imager
AVHRR: Advanced Very High Resolution Radiometer flown on NOAA satellites
BIOMASS: Biomass monitoring mission for Carbon Assessment, 7th of the European Union’s Earth Explorer (EE) missions
BOREAS: BOreal Ecosystem Atmsophere Study
C-band: Radar designation for wavelengths of 4–8-cm length and frequencies of 4–8 GHz
CFS: Canadian Forest Service
CGM: Climate Modeling Grid, a global 5.5-km grid
CHIME: Copernicus Hyperspectral Imaging Mission; proposed (S-19) high-priority mission for Copernicus-2.0
CHRIS: Compact High Resolution Imaging Spectrometer onboard the CubeSat, PROBA-1 (PRoject for On-Board Autonomy-1)
CIMR: Copernicus Imaging Microwave Radiometer mission; proposed high-priority mission for Copernicus-2.0
CNES: Centre National d’Etudes Spatiales, French Space Agency
CO_2_M: Copernicus Anthropogenic Carbon Dioxide Monitoring; proposed (S-7) high-priority mission for Copernicus-2.0
CONUS: Contiguous continental US, applies to satellites that cover the contiguous states of the USA
COST: European Cooperation in Science and Technology
CRISTAL: Copernicus Polar Ice and Snow Topography Altimeter mission; proposed high-priority mission for Copernicus-2.0
CSA: Canadian Space Agency
CZCS: Coastal Zone Color Scanner, airborne prototype for the SeaWifs, the first ocean color satellite
DEM: Digital Elevation Model
DESIS: DLR Earth Sensing Imaging Spectrometer flying on the ISS
DLR: German Aerospace Center (Deutches Zentrum für Luft-und Raumfahrt)
DMB: Day-Night Band on the VIIRS satellite
DMSP: U.S. Defense Meteorological Satellite Program
ECCC: Environment and Climate Change Canada
ECOSTRESS: ECOsystem Spaceborne Thermal Radiometer Experiment on the International Space Station
EE: Earth Explorer program for the European Union
EMIT: Earth Surface Mineral Dust Source Investigation, a future mission for the International Space Station
EnMAP: Environmental Monitoring and Analysis Program, an upcoming hyperspectral satellite sponsored by Germany
EnviSat: Environmental Satellite of the European Space Agency
EO-1: Earth Observer-1 demonstration satellite from NASA
EOS: Earth observing system
EOSAT: Earth Observation Satellite Company
EROS: Earth Resources Observation and Science (EROS) Center, USGS in Sioux Falls, SD
ERS: European Remote Sensing, Radar Satellites (ERS-1 and ERS-2)
ERTS: NASA’s Earth Resources Technology Satellite, later renamed Landsat-1
ESA: European Space Agency of the European Union
ET: Evapotranspiration, water lost to the atmosphere from plants and soil
ETM+: Enhanced Thematic Mapper for Landsat 7
EU: European Union
EUMETSAT: European Organization for the Exploration of Meteorological Satellites
EVI: Enhanced Vegetation Index
EnviSat: ESA’s Environmental satellite with 9 instruments for Earth observation, including MERIS, operated 2002–2012; replaced by Sentinel program
FCI: Flexible Combined Imager, on the MTG-I GEO stationary satellites
FIFE: First International Field Experiment
FIR: Far infrared, wavelength longer than 15 μm and up to 1000 μm
FLEX: Fluorescence Explorer mission, 8th of the European Union’s Earth Explorer (EE) missions (EE-8)
FLORIS: FLuORescence Imaging Spectrometer for the Fluorescence Explorer mission
FPAR: Fraction of Photosynthetically Active Radiation
GEDI: NASA’s Global Ecosystems Dynamics Investigation LiDAR on the International Space Station
GEO: Satellite in geostationary orbit
GEOCARB: NASA’s future Carbon Cycle Observatory in GEO orbit
GHz: GigaHertz, equals 1 billion Hz (1,000,000,000 Hz), expresses wave frequency.
GLM: Geostationary Lightning Mapper, a GOES-R instrument
GMES: Global Monitoring for Environment and Security program, managed by European Space Agency (ESA) and European Environment Agency (EEA), includes the Sentinel program and “Contributing Missions” that provide complementary observation capacity.
GOES: NOAA’s Geostationary Operational Environmental Satellites. GOES-16 and GOES-17 are first of the 3rd-generation geostationary weather satellites (coordination between Japan and Europe makes all of these instruments closely similar)
GPM: Global Precipitation Measurement satellite
GPP: Gross Primary Productivity
HH, HV: Emitting or Receiving polarized energy in horizontal (H) or vertical (V) orientations; Designates emitting orientation first and receiving orientation second
HICO: Hyperspectral Imager for the Coastal Ocean previously flown on the ISS
Himawari: 3rd-generation geostationary weather satellite operated by Japan’s Meteorological Agency
HISUI: JAXA Hyperspectral Imager SUIte for the ISS
Hyperion: NASA demonstration hyperspectral imager that flew on EO-1
HyspIRI: Hyperspectral and Infrared Imager, a NASA prototype concept for the future SBG mission
I-bands: Imaging bands on the VIIRS satellite
ICESat: Cloud and land Elevation Satellite mission, with ICEsat-1, and ICEsat-2
IR: Wavelengths longer than visible energy in the solar infrared spectrum (between 700 and 2500 nm)
ISRO: Indian Space Research Organization, within the Department of Space
ISS: International Space Station
JAXA: Japan Aerospace Exploration Agency
JEM: Japanese Experiment Module on the ISS
JPL: Jet Propulsion Laboratory
JPSS: Joint Polar Satellite Program (NOAA and NASA)
L-1 to L-9: Designation number for each Landsat satellite
L-band: Radar designation for wavelengths of 20–30-cm length and frequencies of 2–4 GHz
LAI: Leaf area index
Landsat: Series of U.S. multispectral satellites; longest record of moderate resolution Earth observation satellites
LE: Latent heat of vaporization
LEO: Low Earth orbit for satellites; usually around 700 to 900 km above surface
LI: Lightning imager on geostationary satellites
LP DAAC: NASA Land Processes Data Archive And Distribution Center
LSTM: Copernicus Land Surface Temperature Monitoring mission; proposed (S-8) high-priority mission for Copernicus-2.0
LUE: Light Use Efficiency
M-bands: Moderate spatial resolution bands on the VIIRS instrument
MERIS: European Space Agency’s Medium Resolution Imaging Spectrometer flown on ESA's Envisat-1
METOP: Series of polar LEO orbiting weather satellites operated by EUMETSAT
Meteosat: 3rd-generation GEO weather satellites (MTG) from EUMETSAT, starting in 2021
METI: Japan’s Ministry of International Trade and Industry
MEXT: Japanese Ministry of Education, Culture, Sports, Science and Technology
MHz: MegaHertz, equals 1 million Hz (1,000,000 Hz), expresses wave frequency
MLT: Mean Local Time
MODIS: Moderate Resolution Imaging Spectroradiometer, flown on both Terra and Aqua satellites
μm: Micrometer, unit of length, 1 millionth of a meter (1 × 10^−6^)
MSI: Multispectral imager on Sentinel-2
MSS: MultiSpectral Scanner on Landsats-1–3
MTG-I: Meteosat (METOP-MTG-I A,B,C) 3rd-generation GEO, weather satellites from EUMETSAT
MTG-S: Sounding instrument on the European Meteosat (MTG) satellite
MUSES: A commercial Multi-User System for Earth Sensing on the ISS
MWIR: Mid-wavelength Infrared, in the 3- to 8-μm region of the spectrum
NASA: (U.S.) National Aeronautical and Space Administration
NDVI: Normalized Difference Vegetation Index
NEON: (U.S.) National Ecological Observatory Network
NIR: Near-infrared wavelengths in the range of 700 to 1500 nm
NISAR: NASA and ISRO (Indian Space Agency) SAR Mission
NLI: National Land Imaging program USGS at Earth Resources Observation and Science (EROS) Center, Sioux Falls, SD
nm: Nanometer, unit of length, 1 billionth of a meter (1 × 10^−9^)
NOAA: (U.S.) National Oceanic and Atmospheric Administration
NOAA 16/17: Third-generation NOAA GOES weather satellites (numbers 16 and 17 of the GOES series)
NOAA 20: Polar orbiting LEO weather satellite with 5 instruments, first of the JPSS series
NPOESS: National Polar Orbiting Environmental Satellite System
NPP: Net Primary Productivity (ecological definition)
NPP: National Polar-Orbiting Partnership renamed Suomi NPP
NRCan: Natural Resources Canada
NSF: (U.S.) National Science Foundation
OCI: Ocean Color Instrument, forthcoming on NASA's PACE satellite
OCO-2: NASA Orbiting Carbon Observatory-2, a free-flying satellite
OCO-3: NASA Orbiting Carbon Observatory-3, flown on the ISS
OLCI: Ocean and Land Colour Instrument on Sentinel-3 satellite series
OLI: Operational Line Imager, the multispectral imager on Landsats-8 and 9
OLS: Operational Line Scanner on the DMSP satellite
ONR: Office of Naval Research (USA)
PACE: Plankton, Aerosol, Cloud, ocean Ecosystem, a forthcoming NASA polar/LEO mission to monitor ocean biology, aerosols, and clouds
PACUS: Pacific region and contiguous U.S., applies to NOAA GOES-17 satellite covering eastern North America to the western Pacific Ocean
PAN band: Broadband panchromatic imaging band sensitive to light across all or most of the visible spectrum
PAR: Photosynthetically active radiation
P-band: Radar designation for wavelengths of 60 cm to more than 1-m length and frequencies of 500–250 MHz
PET: Potential evapotranspiration; based on physical driving variables
PHyTIR: Prototype HyspIRI Thermal Infrared Radiometer, airborne hyperspectral sensor that was a prototype for ECOSTRESS
PLE: Potential latent heat of vaporization, based on physical variables to change from liquid to vapor
PRISMA: PRecursore IperSpecttrale della Missione Applicativa (Precursor Imaging Spectrometer for Mission Applications), an Italian spectroscopy satellite mission
PROBA-1: Project for On-Board Autonomy-1, a small ESA CubeSat carrying the CHRIS imager
RapidEye: Constellation of five high spatial resolution commercial satellites
RFP: Fire Radiative Power
ROSE-L: L-band Synthetic Aperture Radar mission, proposed high-priority mission for Copernicus-2.0
S-1 to S-n: Sentinel satellites, currently S-1 to S-6, ESA's operational satellites
SAR: Synthetic Aperture Radar
S band: Radar designation for wavelengths of 8–15-cm length and frequencies of 2–4 GHz
SBG: Surface Biology and Geology mission, a high-priority NASA satellite under development
SCIAMACHY: SCanning Imaging Absorption spectroMeter for Atmospheric CHartographY
SeaWIFS: Sea-viewing Wide Field-of-view Sensor, the first global satellite mission for ocean color
Sentinel: Series of operational satellites (1–6 +5P) flown by the European Union as part of their initial Copernicus (1.0) program; 2.0 includes plans for 7–10 additional satellites
SIF: Solar-induced chlorophyll fluorescence
SkySat: Planet’s commercial CubeSat constellation 1–7, formerly SkyBox Imaging owned by Terra Bella
SLC: Scan Line Corrector, a critical failure in Landsat 7
SLI: Sustainable Land Imaging for Landsat programmatic development
SLSTR: Sea and Land Surface Temperature Radiometer, one of two imagers on S-3
SMAP: Soil Moisture Active Passive satellite
SNR: Signal to noise ratio
SPOT: Satellite Pour l’Observation de la Terre, translated as Satellite for Observation of the Earth (CNES), now run by Spot Image and Airbus, 1–7 commercial satellites
Suomi NPP: The Suomi National Polar-orbiting Partnership, previously known as the National Polar-orbiting Operational Environmental Satellite System Preparatory Project (NPP) and as NPP-Bridge. It is named for Verner E. Suomi, an influential meteorologist at the University of Wisconsin that helped start the US weather satellite porgram
SWIR: Wavelengths (between 1500 and 2500 nm) in the long wavelength part of the solar electromagnetic spectrum
TanDEM-X: Digital Elevation Measurements (DEM) add on satellite to TerraSAR-X, an X-band SAR radar mission
Terra: EOS flagship satellite launched in 1999 with five instruments in the midmorning equatorial crossing time, including MODIS and ASTER
TerraSAR-X: High spatial resolution 3m X-band SAR radar mission
TIR: Thermal Infrared wavelengths between 8 and 15 μm, also called long-wavelength infrared
TIRS (1, 2): Thermal InfraRed Sensor, two-band thermal sensor on Landsats L-8 and L-9
TM: Thematic Mapper multispectral imager and TIR imaging band on Landsats L4–L6
TOA: Top of Atmosphere (in reference to reflectance)
TOPSAR: Terrain Observation with Progressive Scans SAR
TROPOMI: TROPOspheric Monitoring Instrument on Sentinel-5P a polar/LEO satellite
USGS: (U.S.) Geological Survey
VENμS: Vegetation and ENvironment monitoring on a Micro-Satellite (France and Israel)
VIIRS: Visible Infrared Imaging Radiometer Suite on the Suomi NPP satellite and on the JPSS series, first being the NOAA 20 satellite
VIS: Wavelengths (between 400 and 70 nm) in the visible part of the solar electromagnetic spectrum
VV, VH: Emitting or Receiving polarized energy in vertical (V) or horizontal (H) orientations, designated emitting orientation first and receiving orientation second
WildFireSat: Canadian Space Agency’s proposed satellite to monitor wildfires. Includes bands from VIS to TIR
X-band: Radar designation for wavelengths of 2–5-cm length and frequencies of 8–12 GHz
